# Expression of the Retrotransposon *Helena* Reveals a Complex Pattern of TE Deregulation in *Drosophila* Hybrids

**DOI:** 10.1371/journal.pone.0147903

**Published:** 2016-01-26

**Authors:** Valèria Romero-Soriano, Maria Pilar Garcia Guerreiro

**Affiliations:** Grup de Genòmica, Bioinformàtica i Biologia Evolutiva, Departament de Genètica i Microbiologia (Edifici C), Universitat Autònoma de Barcelona, 08193, Bellaterra, Barcelona, Spain; Virginia Tech Virginia, UNITED STATES

## Abstract

Transposable elements (TEs), repeated mobile sequences, are ubiquitous in the eukaryotic kingdom. Their mobilizing capacity confers on them a high mutagenic potential, which must be strongly regulated to guarantee genome stability. In the *Drosophila* germline, a small RNA-mediated silencing system, the piRNA (Piwi-interacting RNA) pathway, is the main responsible TE regulating mechanism, but some stressful conditions can destabilize it. For instance, during interspecific hybridization, genomic stress caused by the shock of two different genomes can lead, in both animals and plants, to higher transposition rates. A recent study in *D*. *buzatii—D*. *koepferae* hybrids detected mobilization of 28 TEs, yet little is known about the molecular mechanisms explaining this transposition release. We have characterized one of the mobilized TEs, the retrotransposon *Helena*, and used quantitative expression to assess whether its high transposition rates in hybrids are preceded by increased expression. We have also localized *Helena* expression in the gonads to see if cellular expression patterns have changed in the hybrids. To give more insight into changes in TE regulation in hybrids, we analysed *Helena*-specific piRNA populations of hybrids and parental species. *Helena* expression is not globally altered in somatic tissues, but male and female gonads have different patterns of deregulation. In testes, *Helena* is repressed in F1, increasing then its expression up to parental values. This is linked with a mislocation of *Helena* transcripts along with an increase of their specific piRNA levels. Ovaries have additive levels of *Helena* expression, but the ping-pong cycle efficiency seems to be reduced in F1 hybrids. This could be at the origin of new *Helena* insertions in hybrids, which would be transmitted to F1 hybrid female progeny.

## Introduction

Hybridization between species is well-known to cause genomic stress that leads to genetic instability in offspring. Hybrids show several features, including polyploidy (common in plants), high rates of chromosomal rearrangements, increased mutation rates, and high transpositional activity [1]. These genome reorganizations are often considered *dysfunctions*, but several cases of hybrid speciation show their evolutionary potential (reviewed in [1]). Furthermore, interspecific hybridization, previously considered a very rare phenomenon in nature, is now estimated to occur in 25% of plants and 10% of animal species, suggesting that its potential has been largely underestimated [2].

Transposable elements (TEs) are dispersed repeated sequences found in the vast majority of the genomes of sequenced species. They have been proposed as major drivers of the genome reorganization occurring during hybridization. Both in animals [3,4] and plants [5–7], examples of TE mobilization due to interspecific crosses have been reported. In *Drosophila*, the pDv111 element transposes in *D*. *virilis—D*. *littoralis* hybrids [8], as does the retrotransposon *Osvaldo* in *D*. *buzzatii—D*. *koepferae* hybrids [9]. A whole-genome study of the latter hybrids using AFLP markers [10] found 70% of the hybrid instability to be caused by transposition events [11]. Increase of *Osvaldo* expression also occurred in hybrid testes [12]. In the same way, a widespread derepression of TEs at the expression level was noted in hybrids of *D*. *melanogaster* and *D*. *simulans* [13].

TEs can be divided in two classes, according to their transposition mechanism [14]. DNA transposons (or class II transposons) are TEs that do not require an RNA intermediate to mobilize: they are excised from their insertion site and inserted in a new position in the genome. Elements of class I, called retrotransposons (or RNA transposons), need a retrotranscription step to transpose: the TE is transcribed, its mRNA reverse transcribed and the resulting cDNA integrated in a new site. A recent classification divides each class into orders and superfamilies according to a more detailed description of their replication strategy and structural features [15]. Long Insterspersed Nuclear Elements (LINEs) are present throughout the eukaryotic kingdom, constituting one of the five distinct orders of class I elements included in this classification, characterized by the production of 5’-end truncated copies.

*Helena* is a LINE-like retrotransposon first described in *D*. *virilis*, as being responsible for one of the isolated mutations in the offspring of hybrid dysgenic crosses [16]. More recently, it has been found at different stages of its life cycle across the *Drosophila* genus [17]: absent or present, autonomous or not, and expressed or silenced. Although it is present in 8 out of 12 *Drosophila* sequenced genomes, its expression has only been detected in *D*. *mojavensis* [17] and some strains of *D*. *simulans* [18]. Our latest results showed that this element is also expressed in *D*. *koepferae* and *D*. *buzzatii*, with an increase in its transposition rate occurring in their hybrids (10^−2^) compared to parental species (0-10^-3^) [11].

On the other hand, TE expression in *Drosophila* is regulated through two small RNA-mediated silencing pathways. In somatic cells, the endogenous small interference RNA (endo-siRNA) pathway is the main TE silencing mechanism [19–22]. In gonads, the Piwi-interacting RNA (piRNA) pathway is important in TE repression at both transcriptional and post-transcriptional level [23]. The primary piRNA biogenesis involves the cleavage of long piRNA precursors, transcribed from specific genomic piRNA clusters [24]. These are loaded into a piRNA amplification loop, called the ping-pong cycle, that gives rise to secondary piRNAs [24].

**Fig 1:**
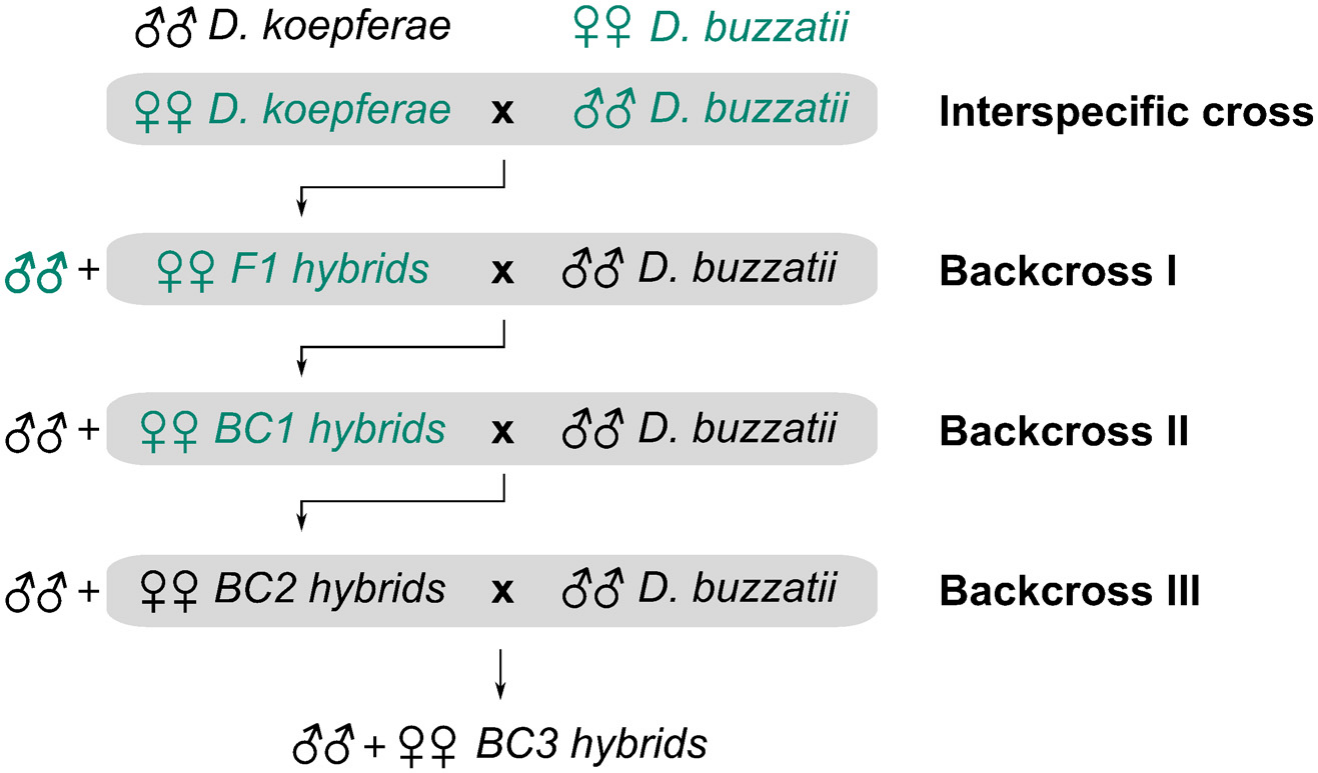
Crosses diagram. A first interspecific cross of 10 *D*. *koepferae* females with 10 *D*. *buzzatii* males was followed by three successive backcrosses of hybrid females with *D*. *buzzatii* males. Samples whose piRNA populations have been analysed are marked in green.

Our aim has been to disentangle the molecular mechanisms responsible for transposition bursts during hybridization events. Initially we molecularly characterized the retrotransposon *Helena* in our target species, *D*. *buzzatii* and *D*. *koepferae*. Subsequently, analyses were run at three levels:

1. Quantification of *Helena* expression by quantitative real time PCR (qRT-PCR) in the offspring of crosses between *D*. *koepferae* females and *D*. *buzzatii* males (the reciprocal cross being unsuccessful), as well as in three subsequent generations of backcrossing hybrid females with *D*. *buzzatii* males (Fig 1). The effects of *D*. *buzzatii* introgression in our hybrid genomes are particularly interesting since F1 males are sterile and the genetic variability created through interspecific hybridization can only be maintained by mating F1 females with parental males. Furthermore, increase of *Helena* transposition seen previously took place mainly in BC2 [11].
2. Localization of *Helena* transcripts in testes and ovaries by fluorescent *in situ* hybridization (FISH), to see if qualitative changes in *Helena* cellular expression patterns occurred after interspecific hybridization.
3. Analysis of *Helena* piRNA populations in germinal tissues of parents and hybrids, because breakdown of the TE silencing mechanisms could be responsible for their derepression in hybrids.

Unexpectedly, *Helena* expression tended to decrease in F1 hybrid testes compared to parental species. This repression might be explained by the high levels of *Helena*-specific piRNAs in hybrid testes, which seem to be mainly produced by the primary piRNA biogenesis pathway. The abundance of *Helena* transcripts in ovaries was significantly different between *D*. *koepferae* and *D*. *buzzatii*, but all hybrids have intermediate values. However, the ping-pong signature decreased, especially in F1 hybrid ovaries. Thus, a partial failure of the ping-pong amplification loop seems to be responsible for derepression of *Helena* in hybrid ovaries, which may sometimes be at the origin of transposition events. However, this activation seems to be compensated in some way by the production of *Helena*-specific primary piRNAs.

## Results

### *Helena* characterization in *D*. *buzzatii* and *D*. *koepferae* species

To characterize *Helena* and analyse its expression, the preliminary goal was to determine the sequence of this TE, which has not previously been done in our target species. From the *Helena* sequence of *D*. *mojavensis* [17], the most closely related sequenced species, we amplified a fragment of the TE in *D*. *buzzatii* (one copy) and *D*. *koepferae* (three copies: Table 1). For *D*. *buzzatii*, a 3840 bp sequence was obtained that covers 85% of the *D*. *mojavensis* consensus copy with 88% of identity, including almost the entire coding region of *Helena*. Even if the sequence is not a complete copy, two overlapping ORFs were identified; the first (*gag-like* protein) harbours a conserved PRE_C2HC domain (upstream of Cys-Cys-His-Cys Zn finger domain), and the second (*pol-like* protein) contains an exonuclease-endonuclease-phosphatase (EEP) as well as a reverse transcriptase domain. No premature stop codons were present, suggesting that the cloned amplicon could be an active *Helena* copy, although the complete sequence of this insertion is needed for confirmation. For *D*. *koepferae*, three different copies were sequenced, two of them (called *35–1* and *35–2*, with 3222 and 3247 bp, respectively) using a long template PCR system, and the other one (called *28*, with 2806 bp) using a different pair of primers. These sequences cover 62–72% of the *D*. *mojavensis* consensus copy with an identity of 86–88%. ORF1 (*gag*) seemed to be complete in two of the copies (*35–2* and *28*), but ORF2 (*pol*) carried deletions and was interrupted by premature stop codons in all three copies. However, all the described conserved domains could be identified and the two ORFs also overlapped in the three sequences.

**Table 1:** Characterization of *Helena* sequenced copies in *D*. *buzzatii* and *D*. *koepferae*.

Species	Length (bp)	Alignments vs. *D mojavensis* consensus [17]			Conserved domains			ORFs	
		% coverage	% identity	E-value	PRE_C2HC	EEP	RT	ORF1	ORF2
***D*. *buzzatii***	3840	85	88	0	+	+	+	+	+
***D*. *koepferae* 28**	2806	62	87	0	+	+	+	+	stop
***D*. *koepferae* 35–1**	3222	71	88	0	+	+	+	stop	stop
***D*. *koepferae* 35–2**	3247	72	86	0	+	+	-	+	stop
***D*. *mojavensis***	4502	-	-	-	+	+	+	+	+

PRE_C2HC: upstream to Cys-Cys-His-Cys (Zn finger motif) domain, EEP: Endonuclease, Exonuclease, Phosphatase; RT: Reverse Transcriptase. For conserved domain analysis [25]: “+” indicates domain presence and “–” indicates domain absence; for ORF analysis: “+” indicates untruncated gene and “stop” indicates that the ORF is interrupted by a stop codon.

Alignments of the *Helena* sequenced copies showed a high degree of sequence identity between *D*. *buzzatii* and *D*. *koepferae*, from 89 to 98% (S1 Table). Interestingly, the closest match was the unique copy of *D*. *buzzatii* and *D*. *koepferae*-28, being *D*.*koepferae*-35-1 the most divergent sequence. *D*. *koepferae*-35-1 and 35–2 share several internal deletions (two short deletions of 12 and 17 bp and a long one of 557 bp) compared to *D*. *buzzatii*. *D*. *koepferae*-35-1 also carries another 43 bp deletion and two short insertions of 9 and 6 bp. Although *D*. *koepferae*-28 does not seem to have any deletion compared to *D*. *buzzatii*, it is noteworthy that a different reverse primer was used to amplify this copy of *Helena*, and the presence of mutations after the primer region cannot be discarded.

**Fig 2:**
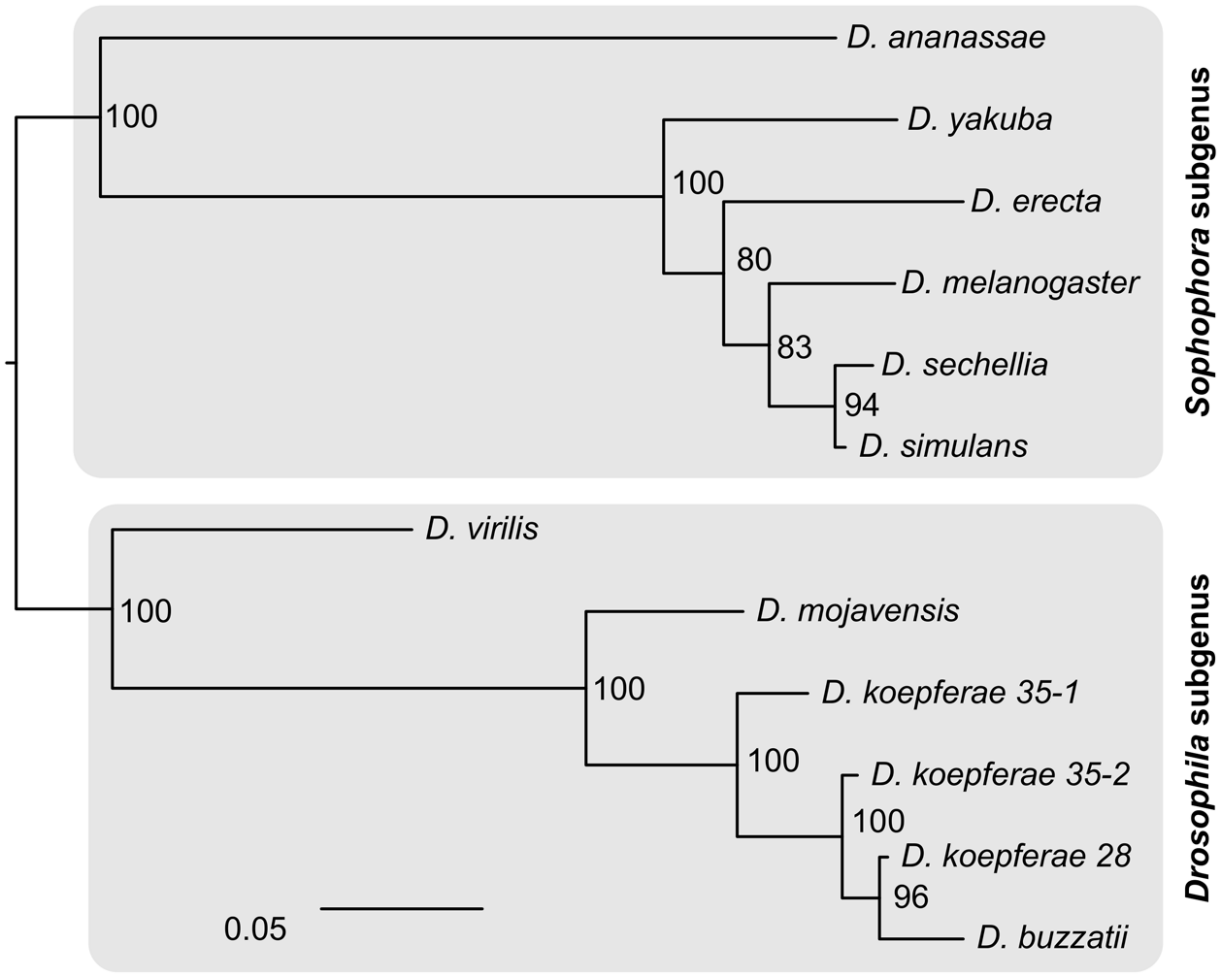
Maximum likelihood phylogenetic tree of *Helena* in the *Drosophila* genus, rooted using the midpoint-root option. Sequences are identified by the host species name. Numbers indicate nodal support, calculated using RAxML with 100 bootstrap replicates.

A phylogenetic analysis of the *Helena* consensus copy identified in all *Drosophila* sequenced genomes [17,18] and other *Helena* characterized sequences [16,26] was made, together with our four sequences. The phylogenetic tree (Fig 2) divides the sequences in two clades that correspond to the *Drosophila* and *Sophophora* subgenera. The *Sophophora* clade is in concordance with its species phylogeny, except for *D*. *erecta*, which is actually grouped with *D*. *yakuba*. Within the *Drosophila* clade, *D*. *buzzatii* and *D*. *koepferae* form a monophyletic cluster, which is a sister group to *D*. *mojavensis*, expected in accordance with a vertical transmission scenario. According to this analysis, *D*. *buzzatii* and *D-koepferae*-28 have the closest related sequences, whereas *D*. *koepferae*-35-1, as previously seen in the alignments, is the most divergent copy.

The *Helena* copy number in the parental species was estimated by Southern blot (S1 Fig), a technique that allows the detection of both euchromatic and heterochromatic insertions. *D*. *buzzatii* has a higher *Helena* copy number (12–15 copies) than *D*. *koepferae* (6–12 copies). These results are in agreement with previous studies by FISH on polytene chromosomes [11], where 12 *Helena* euchromatic copies were detected in *D*. *koepferae* and 5 in *D*. *buzzatii*.

### Overview of *Helena* expression in parental species

*Helena* expression has first been quantified in parental species by quantitative reverse transcription PCR (qRT-PCR), to evaluate differences in expression rates between *D*. *koepferae* (female parental species, Fig 1) and *D*. *buzzatii* (male parental species). Expression rates (ERs) were estimated using the comparative C_T_ method [27]. No introns have been described in the *Helena* sequence of any of the species in which this TE has been characterized [16–18,26,28]. Furthermore, the amplified fragment had the same length in both parental species, whether we used DNA or cDNA as a template [11], showing that our analyses concerned the only *Helena* splicing variant.

Germinal (testes or ovaries) and somatic tissues (*i*.*e*. male or female carcasses lacking testes or ovaries) were investigated separately. The results (summarized in S1 File) showed that ERs in somatic tissues are similar between sexes and species (ER≈10^−4^, Fig A in S1 File). Indeed, neither the Wilcoxon rank sum test (which compares pairs of samples) nor the Kruskal-Wallis test (which compares multiple samples at once, χ^2^ = 2.7139; p-value = 0.4379) show significant differences between somatic tissue samples (Fig C in S1 File). For gonadal samples, the ERs were higher in the testes than the ovaries (Fig B in S1 File); in this case differences between sexes were statistically significant (Wilcoxon’s W = 5, p-value = 4.9×10^−7^, considering all parental samples). Concerning differences between species, *Helena* ERs in ovaries were significantly higher for *D*.*buzzatii* than *D*. *koepferae* (Fig D in S1 File, p-value = 6.66×10^−4^). However, expression in the testes of the parental species was not significantly different. Contrary to the results in somatic tissue, the Kruskal-Wallis test indicated significant differences in gonads (χ^2^ = 22.7049; p-value = 4.653×10^−5^). It is worth noting the presence of some outlier replicates with particularly high ER values, which occurs mostly in *D*. *buzzatii*, the parental species with the highest transposition rate (8.2×10^−3^ vs. 0 in *D*. *koepferae* [11]). However, variances between parental species ERs were only statistically different in the ovaries (Levene’s test, S2 Table).

### *Helena* expression in hybrid somatic tissue

**Fig 3:**
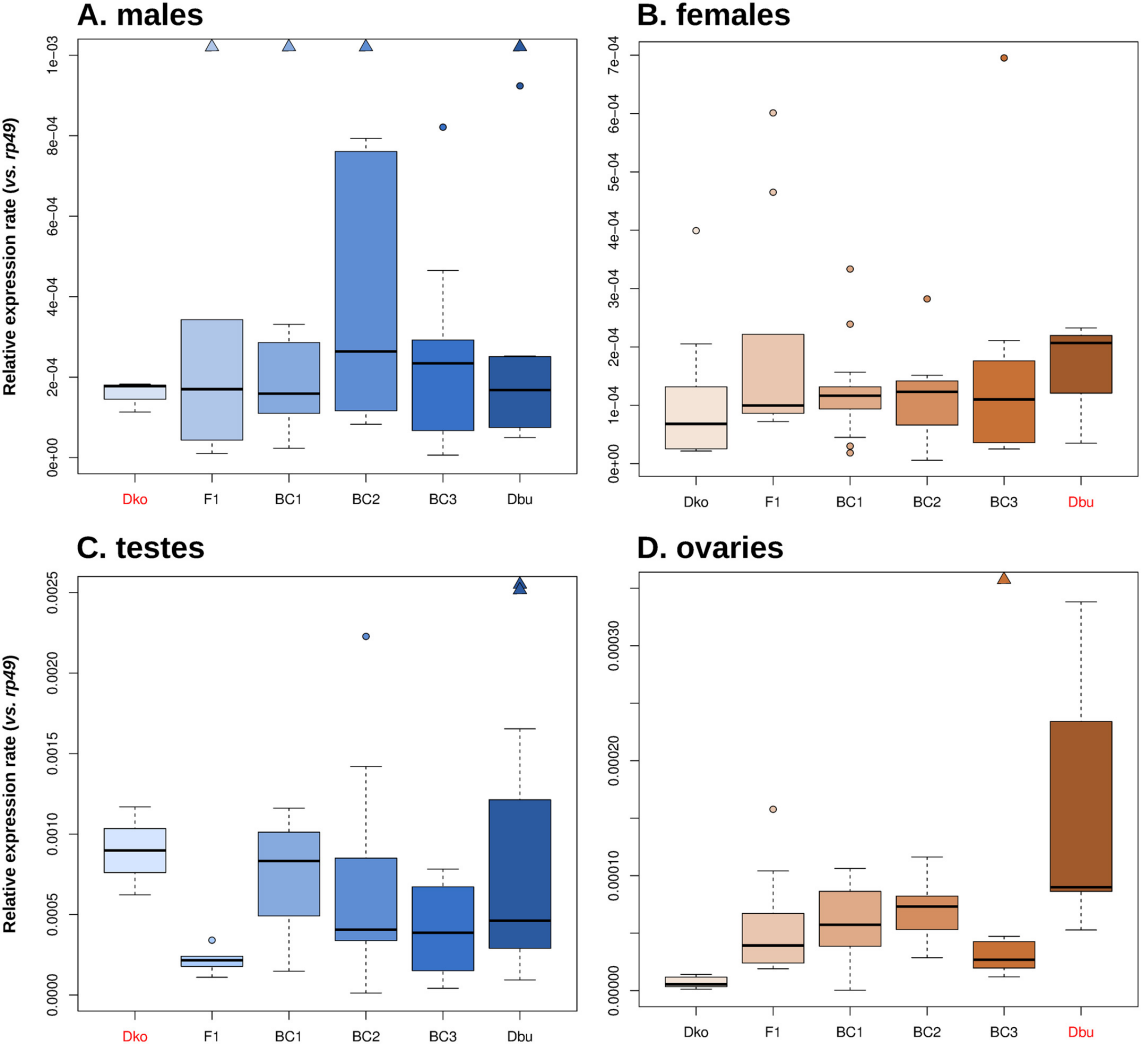
*Helena* expression rates relative to *rp49* housekeeping gene in parental species (*Dko* and *Dbu*) and hybrids. Boxes are determined by the first and third quartile values, with an intermediate deep line corresponding to the median value. Circles correspond to outliers (above or below 1.5-fold the interquartile range), and triangles represent those outliers whose ERs are extremely outranged and cannot be represented in the same scale. Male samples are represented in blue and female samples are represented in brown: the darker the colour, the higher the *D*. *buzzatii* genome fraction. Parental species which are not part of the interspecific crosses (*i*.*e*., *Dko* for male tissues and *Dbu* for female tissues) are marked in red. **A)** results of male somatic tissues (outranged values represented by triangles are: ER = 4.5×10^−2^ for F1, ER = 1.7×10^−3^ for BC1, ER = 4.3×10^−3^ for BC2. ER = 2.9×10^−3^ for *Dbu*), **B)** results of female somatic tissues, **C)** results of testes (*Dbu* outranged values represented by triangles are: ER = 6.2×10^−3^ and ER = 3.6×10^−3^), **D)** results of ovaries (BC3 outranged value represented by a triangle: ER = 8.5×10^−3^).

The ERs of *Helena* retrotransposon were investigated across four sequential generations of *D*.*buzzatii*-*D*.*koepferae* hybrids, a first interspecific cross and three subsequent backcrosses of hybrid females with *D*. *buzzatii* males (Fig 1). Our aim was to compare the ERs of each hybrid generation (F1, BC1, BC2 and BC3) with both parental species values. It is noteworthy that expression values of *D*. *koepferae* female samples (female parental species) and *D*. *buzzatii* male samples (male parental species) were those of the individuals involved in crosses to obtain F1 hybrids (each replicate belonging to a different cross). However, because *D*. *buzzatii* females and *D*. *koepferae* males are not involved in hybrid crosses, several individuals from the same laboratory stocks used in hybrid crosses were analyzed (Fig 3, in red).

In the case of males (Fig 3A), there were no obvious differences between parental and hybrid values for most of the replicates (average median of generations: ER = 2×10^−4^), although BC2 samples seem to have slightly higher expression rates compared to the other generations. However, neither the Wilcoxon rank sum test (Table 2) nor the Kruskal-Wallis test (χ^2^ = 1.931; p-value = 0.8586) showed significant differences between hybrids and parental species. There were a few outranged values (considering ER≥10^−3^, represented by triangles in Fig 3), which might have been due to occasional transcription bursts taking place in F1, BC1 and BC2 (ER = 4.5×10^−2^, 1.7×10^−3^ and 4.3×10^−3^, respectively). Indeed, Levene’s test for equality of variances shows that there were significant differences between generations (taking into account all samples: W = 1.45, p-value = 6.73E-06) and, in particular, F1 males had increased variance compared to *D*. *buzzatii* (S2 Table).

**Table 2:** Comparisons of *Helena* expression rates between each hybrid generation and both parental species (*D*. *buzzatii* and *D*. *koepferae*).

		N	median	SD	vs.*D*. *buzzatii*		vs. *D*. *koepferae*	
					W	p-value	W	p-value
**males**	**F1**	6	1.70E-04	1.81E-02	35	8.84E-01	9	1.00E+00
	**BC1**	11	1.59E-04	4.76E-04	59	9.49E-01	15	8.85E-01
	**BC2**	10	2.64E-04	1.29E-03	39	2.82E-01	8	2.87E-01
	**BC3**	9	2,34E-04	2.54E-04	48	9.41E-01	11	7.27E-01
**females**	**F1**	9	9.98E-05	1.92E-04	13	1.00E+00	23	1.36E-01
	**BC1**	13	1.16E-04	8.48E-05	24	6.11E-01	47.5	4.83E-01
	**BC2**	12	1.23E-04	7.31E-05	24	4.48E-01	47	6.51E-01
	**BC3**	10	1.10E-04	2.00E-04	19	5.54E-01	37	5.40E-01
**testes**	**F1**	5	2.16E-04	8.49E-05	47	2.75E-02*	15	3.57E-02*
	**BC1**	11	8.33E-04	3.52E-04	53	6.52E-01	21	5.55E-01
	**BC2**	14	4.06E-04	5.95E-04	83.5	7.43E-01	32	1.86E-01
	**BC3**	10	3.86E-04	2.56E-04	67	4.26E-01	27	4.90E-02*
**ovaries**	**F1**	12	3.93E-05	4.13E-05	52	1.94E-02*	0	3.09E-06***^,^^a^
	**BC1**	13	5.73E-05	3.23E-05	54.5	3.40E-02*	10	2.43E-04***^,^^a^
	**BC2**	14	7.31E-05	2.65E-05	55	9.45E-02	0	1.75E-06***^,^^a^
	**BC3**	12	2.68E-05	2.44E-03	0	6.14E-03**^,^^a^	2	1.24E-05***^,^^a^

N = number of replicates analyzed, SD = standard deviation, W = Wilcoxon rank sum test statistic, p-value = probability.

*: p-value < 0.05,

**: p-value < 0.01,

***: p-value < 0.001,

^a^: p-values that are significant after Bonferroni correction (p-value<0.0125). Each kind of sample (males, females, testes, ovaries) has been compared to the same tissue of both parental species (see S1 File for N, median and SD values of parental species groups).

For females (Fig 3B), similar *Helena* ERs between generations were detected (average median of generations: ER = 10^−4^). The highest expression rates belonged to one BC3 and some F1 biological replicates, but none of them reached the ER = 10^−3^ threshold. At a statistical level, there were no significant differences between parents and hybrids (Table 2), the groups not being distinguishable (Kruskal-Wallis test, χ^2^ = 2.6058; p-value = 0.7605), nor were their variances statistically different (Levene’s test, W = 1.56, p-value = 0.1893 and S2 Table).In conclusion, *Helena* expression rates in somatic tissue do not change significantly after interspecific hybridization. However, in males, a few exceptional crosses gave outranged ER values responsible for the increase of variance between F1 hybrids and parents.

### *Helena* expression in hybrid germinal tissue

Analogous experiments and analyses to those on somatic tissues were carried out on gonads of both males and females. ERs in testes (Fig 3C) were globally higher than in somatic tissues (average median between generations: ER = 4×10^−4^). Comparing ERs between different generations, F1 testes seem to have the lowest transcript levels of the retrotransposon *Helena*; in fact, the Wilcoxon rank sum test indicated significant differences between F1 and both parental species expression rates (Table 2). No statistically significant differences were found between the other hybrid generations and parental species, except for BC3 and *D*. *koepferae* (the parental species showing the highest expression rates), these being at the boundaries of significance (p-value = 0.049, Table 2). The Kruskal-Wallis test also showed significant differences (χ^2^ = 11.2107; p-value = 0.04736), and in this case, the single outlier value seen in hybrids (BC2) proved to be lower than those in *D*. *buzzatii*. Levene’s test indicated that variances were significantly different between generations (including all samples: W = 5.58, p-value = 3.934E-04), mainly due to the higher variance of *D*. *buzzatii* samples compared to hybrids (S2 Table). Thus, *Helena* expression in hybrid testes tends to be similar or lower than in parental species, with lower variances compared to *D*. *buzzatii*.

Ovaries, where TEs are strongly regulated [29], had the lowest *Helena* expression rates (average median between generations of 5×10^−5^). *Helena* expression in *D*. *buzzatii* was significantly higher than in *D*. *koepferae*, and the vast majority of hybrid replicates had intermediate values (Fig 3D). Expression differences between *D*. *koepferae* and each hybrid generation are highly significant (Table 2): expression was higher in hybrids for almost all the replicates of every generation. Furthermore, differences in *Helena* expression rates between *D*. *buzzatii* and hybrids were also significant for F1, BC1 and BC3 (Table 2). *Helena* expression gradually increases across generations F1 to BC2, and then unexpectedly decreases in BC3. Yet, the highest ER (ER = 8.5×10^−3^, in red in Fig 3D) belonged to a BC3 replicate, which might be due to a sporadic transcription burst. However, variance was unchanged in BC3 compared to any of the parental species (S2 Table), although Levene’s test show that there were significant differences between generations (taking into account all samples: W = 4.05, p-value = 3.138E-03). In particular, *D*. *buzzatii* had a more variable *Helena* expression than hybrids, whereas *D*. *koepferae* had the lowest variance (in both cases, results are significant for F1, BC1 and BC2 –S2 Table). Hence, *Helena* expression in ovaries can be considered to be additive between parental species, but the hybrids had higher *Helena* ERs and variances than the maternal species (*D*. *koepferae)*.

### Tissue expression patterns in hybrids and parental species

To see whether the quantitative differences in *Helena* expression between hybrids and parents involved changes in patterns of expression in tissues, FISH was used on male and female gonads (see [30] and [31] for annotated schemes of these tissues).

**Fig 4:**
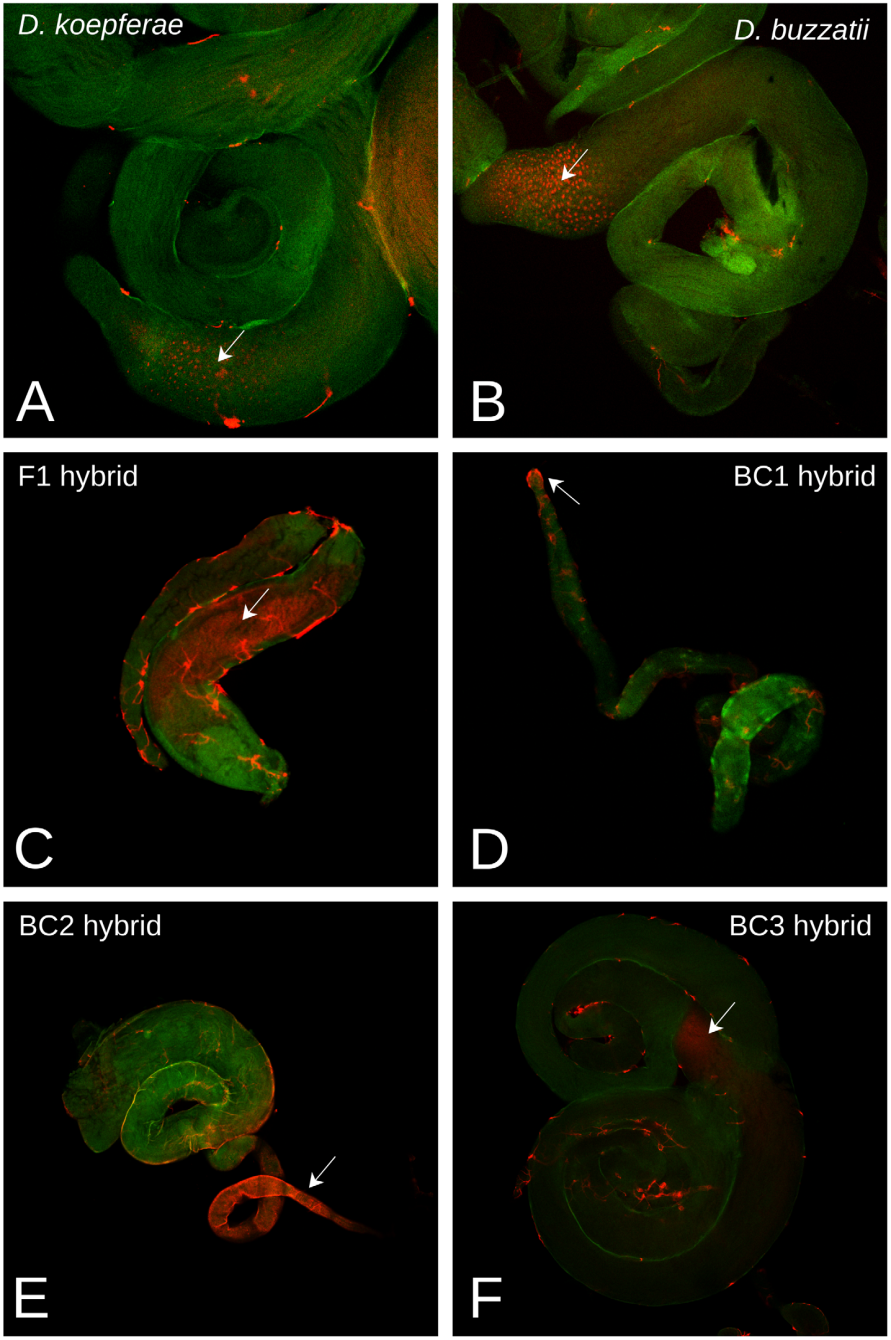
FISH of *Helena* RNA expression in testes. Red staining are *Helena* transcripts, green staining is tissue autofluorescence. Arrows mark the presence of *Helena* transcripts. **A)** *D*. *koepferae*, **B)** *D*. *buzzatii*, **C)** F1 hybrid, **D)** BC1 hybrid, **E)** BC2 hybrid, **F)** BC3 hybrid.

Hybridized testes had *Helena* expression signals in both parental and hybrid germinal tissue (Fig 4), with different patterns between generations. In *D*. *buzzatii* (male parental species), as well as in *D*. *koepferae* (female parental species), *Helena* transcripts were specifically localized in the mitotic spermatogonia region (Fig 4A and 4B). Hybrid expression presented a high variability between generations, as well as differences among individuals from the same generation. For example, in F1, transcripts were mostly detected in the elongating spermatids region (Fig 4C); but some testes had additional expression in the primary spermatocytes (mitotic spermatogonia, as in the parents: Figs A-C in S2 File) or a generalized expression from the apical zone to the elongation area (Figs D and F in S2 File), and only in one case, no detectable expression (Fig E in S2 File). In BC1, no transcript signals were detected in most cases (S3 File), with a few exceptions that had signals at the end of the ejaculatory duct (Fig 4D and Figs B and D in S3 File), where individualized mature sperm can be found [30]. Expression in BC2 testes has also been detected in the basal zone near to the ejaculatory duct (Fig 4E and Figs B, D and E in S4 File), and also in the apical end (Figs A-D and F in S4 File), including in some cases the stem cell area (Figs A and B in S4 File). In BC3, two different patterns were seen (Fig 4F); transcripts were localized in primary spermatocytes (as in parents), or there were no evident hybridization signals.

**Fig 5:**
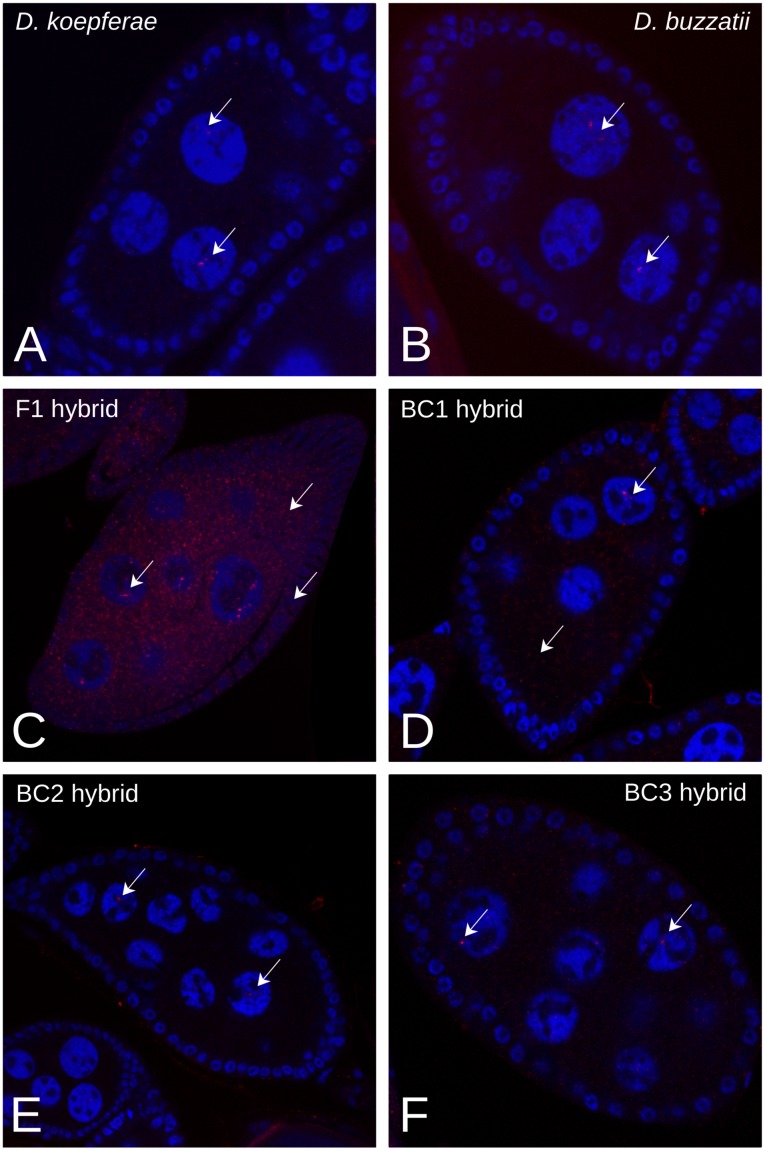
FISH of *Helena* RNA expression in ovaries. Red staining are *Helena* transcripts, blue staining is DAPI (cells nuclei). Arrows mark the presence of *Helena* transcripts. **A)** *D*. *koepferae*, **B)** *D*. *buzzatii*, **C)** F1 hybrid, **D)** BC1 hybrid, **E)** BC2 hybrid, **F)** BC3 hybrid.

In ovaries, *Helena* expression was also detected in all hybrid and parental samples (Fig 5). In both parental species, transcripts were specifically detected in the nurse cell nucleus (Fig 5A and 5B). In F1 hybrid females, there was widespread expression not only in the nucleus but also in the nurse cell cytoplasm; and interestingly, transcripts are also found in follicle cells, which are somatic cells of the germinal tissue (Fig 5C). In the three backcrosses (Fig 5D–5F), *Helena* expression was restricted to nurse cells, and only in BC1 was expression also seen in the cytoplasm of these cells (Fig 5D).

### Comparative analysis of *Helena* piRNA populations in interspecific hybrids

**Fig 6:**
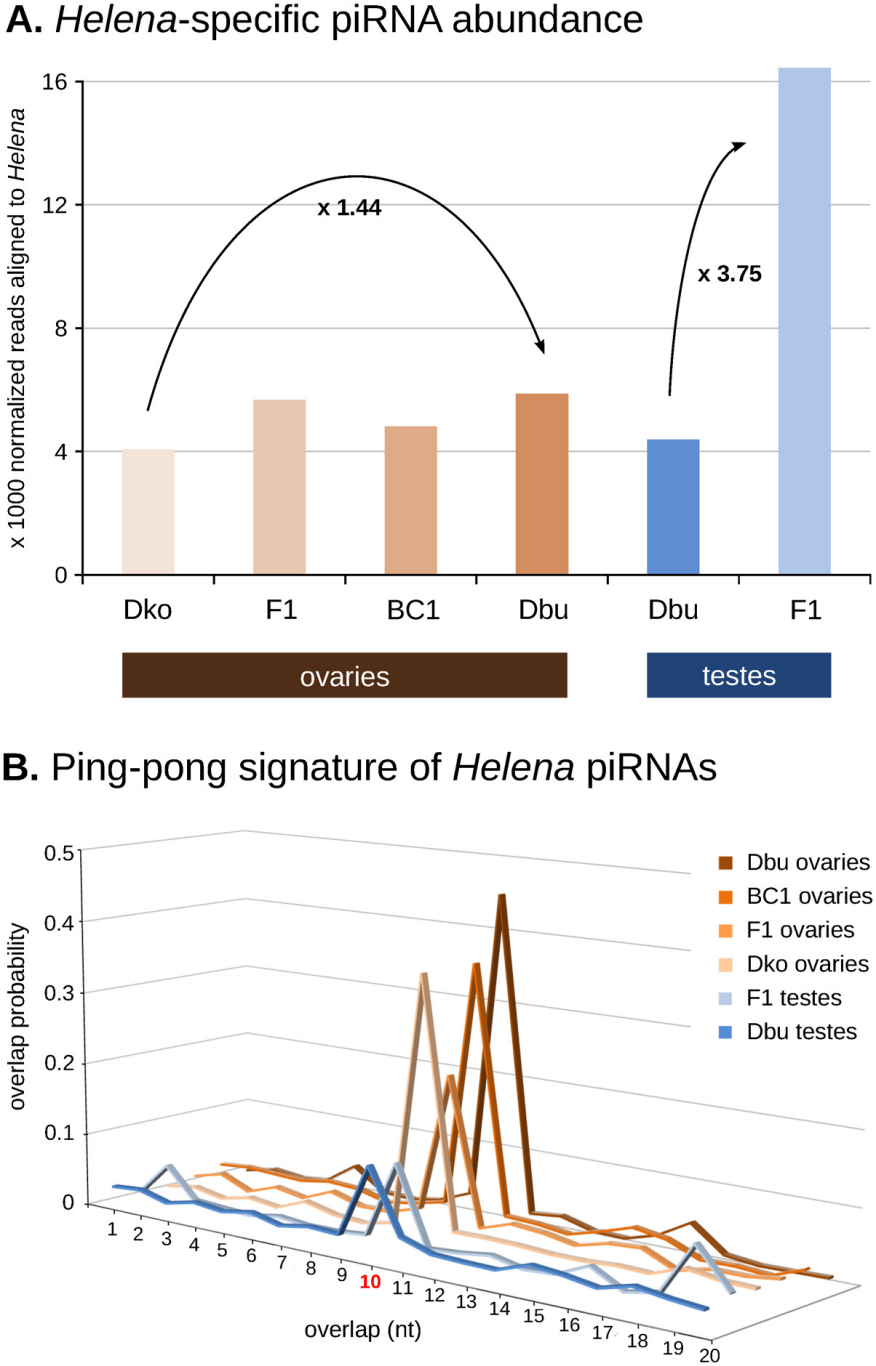
piRNA-mediated regulation of the retrotransposon *Helena*. **A)** Quantification of *Helena* piRNA populations: normalized read count of *Helena*-specific piRNAs in all sequenced samples, **B)** Ping-pong signature of *Helena*-specific piRNAs samples: probability of finding sense-antisense read pairs aligned to *Helena* sequences overlapping by 1 to 20 nucleotides; 10 nt overlap corresponds to ping-pong signal.

For greater insight of *Helena* regulation by the piRNA pathway, we sequenced the gonadal small RNA populations of some of the samples analysed by qRT-PCR (in green, Fig 1). We analysed the alignment of 23–32 nucleotides reads (corresponding to piRNAs length) to all the *Helena* copies described in the *D*. *buzzatii* genome [32], with two main objectives: (i) quantifying the amount of *Helena*-specific piRNAs (Fig 6A), and (ii) detecting the ping-pong signature levels for each sample (Fig 6B).

In the testes, differences in *Helena*-specific piRNA abundance between *D*. *buzzatii* and F1 hybrids were striking; F1 hybrids had a 3.75 fold higher expression of *Helena* piRNAs than their parents (Fig 6A). However, the amounts of ping-pong signature detected in *Helena* piRNAs (Fig 6B) were similar in both samples. Consequently, we hypothesized that an activation of the primary piRNA biogenesis pathway—which acts independently of the ping-pong cycle—might be occurring in hybrid testes. This activation could be at the origin of the repression of the retrotransposon *Helena* found by qRT-PCR (Fig 3C).

We also analysed the piRNA populations of ovaries of both parental species, as well as F1 and BC1 hybrids. *Helena* piRNAs were slightly more abundant in *D*. *buzzatii* than in *D*. *koepferae* (×1.44, Fig 6A), and the ping-pong signal was also higher in *D*. *buzzatii* (10 nt-overlap probability of 45.2 vs. 36.4%, Fig 6B). In hybrid ovaries, *Helena*-specific piRNA amounts were at intermediate values between parental species (Fig 6A). F1 amounts were similar to *D*. *buzzatii*, but decreased after a generation of backcrossing. Curiously, F1 ovaries had a lower ping-pong signal than both parental species (×1.7–2.1, Fig 6B), while the BC1 signal was higher and very similar to *D*. *koepferae*. Thus, the results seem to indicate less efficient ping-pong cycle in hybrids than in parental species. However, this hypothetical partial failure of the ping-pong amplification loop does not seem to alter substantially global piRNA production. As suggested for the testes, the primary pathway of piRNA production might also be responsible for maintaining the levels of *Helena* piRNAs.

## Discussion

### *Helena* is a well-conserved element in *D*. *buzzatii* and *D*. *koepferae* genomes

Our four *Helena* sequenced copies (*Dbu*, *Dko-28*, *Dko-35-1* and *Dko-35-2*) show high intraspecific and interspecific sequence identity levels (93–98%). This degree of conservation is remarkably high for a couple of species whose divergence time has been estimated at ~5 Myr [33], with a dS mode estimated in 0.85 (Romero-Soriano *et al*., in prep). This could mean that *Helena* is under purifiying selection in our model species, but the fact that LINE-like elements transposition mechanism produces non-functional 5’-truncated new insertions, which most probably display neutral evolution patterns [26], makes this hypothesis unlikely. Knowing that *D*. *mojavensis*, the closest sequenced species from *D*. *buzzatii* and *D*. *koepferae*, is one of the few species where a potentially active copy and expression of *Helena* has been detected [17], it is possible that our sequenced copies come from a recent invasion, escaping the genomic control, as in *D*. *mojavensis*. Internal deletions in non-LTR retrotransposons might act as a prevention mechanism against genome invasions by TEs [26,28]. In effect, the three *D*. *koepferae* sequenced copies present truncated *pol* ORFs, rendering them inefficient for transposition. Granzotto *et al*. [17] suggested that the case of *Helena* might offer a unique opportunity to real-time track a TE *life cycle* in *Drosophila*: its study in the few species where it remains active may lead us to understand the molecular mechanisms involved in TE neutralization, from internal deletions to expression repression.

Our phylogenetic analysis shows that the most closely related *Helena* sequences are, unexpectedly, *Dbu* and *Dko-28*, which might be explained by interspecific gene flow between *D*. *buzzatii* and *D*. *koepferae*, since they are sibling species sharing the same habitat in some arid areas of Argentina and Bolivia [34]. Although they are reproductively isolated by hybrid male sterility, introgression is possible through backcrosses of parental males with hybrid females. In fact, interspecific hybridization, eased in nature by sympatry, has been proposed between these species [33,35,36]. Another explanation could be horizontal transfer of *Helena* between these species, given that many examples of genetic horizontal transmission in eukaryotes involving transposable elements have been reported (reviewed in [37]).

### *Helena* somatic expression remains globally unchanged after interspecific hybridization

Quantitative expression results show that the abundance of *Helena* transcripts in somatic tissues is not significantly different from hybrids and parental species (Table 2). However, a few replicates have extremely high ERs, especially in males (Fig 3), which might be due to exceptional transcription bursts. In F1, the presence of outranged values leads to a significant increase in variance (S2 Table), which could also be the consequence of experimental errors. However, we believe our careful controls and technical replicates were sufficiently accurate to rule out this hypothesis.

A small RNA-mediated silencing pathway, the endogenous siRNA (endo-siRNA) pathway, is responsible for TE silencing in *Drosophila* soma [19–22]. Failure of this post-transcriptional silencing system would result in a higher expression of *Helena* (and other TEs), as occasionally noted in our hybrid males. This punctual deregulation can be explained in two ways: (i) by the unique genetic background of each backcrossed hybrid, determined by the introgressed fragments of *D*. *buzzatii* genome. Different genetic backgrounds can result in significant differences in somatic transposition rates [38], which is also concordant with the different transposition rates found between hybrid individuals [11]. Furthermore, the endo-siRNA pathway components have quantitative [38] and overlapping [39] effects, which can lead to a wide range of consequences in case of partial failure. And (ii) because somatic transposition events are cell-specific and can take place at different stages of development [38], leading to different kinds of insertion mosaicisms. The earlier the silencing failure, the more *Helena* expression is expected both in F1 and backcrossed hybrids. So far, we ignore the ultimate cause of the occasional somatic deregulation occurring in a few samples, but it could be caused by either the divergence of the endo-siRNA pathway effector proteins between parental species or by the absence/presence of *Helena*-specific endo-siRNAs in the genomic clusters responsible for their production.

### *Helena* expression is repressed and mislocalized in F1 testes

In the male germline, *Helena* expression is first repressed in F1 hybrids, then restored to approximately the parental levels in subsequent generations (Fig 3C). These results are in contrast with those obtained for another mobilized retrotransposon, *Osvaldo*, in our hybrids in which significantly enhanced expression occurs in hybrid testes [12]. However, these differences are not rare because different classes of TEs can undergo differential regulation [24,40]. In the case of *Helena*, regulation studies provided us with a compelling molecular explanation for *Helena* low transcript abundances in F1; indeed, F1 testes have almost four times more *Helena*-specific piRNAs than the parental species, *D*. *buzzatii*, which might make its silencing more efficient (Fig 6A). It is noteworthy that the ping-pong cycle signature is maintained between F1 hybrids and *D*. *buzzatii* (Fig 6B), showing that the greater abundance of *Helena*-specific piRNA populations is not due to increased efficiency of secondary piRNA biogenesis. Therefore, we can hypothesize that the primary piRNA biogenesis pathway is enhanced in F1 interspecific hybrid testes in order to counterpart a putative TE activation. This was proposed in a recent study on wheat [41], where TE repression mechanisms were activated in F1 hybrids.

On the other hand, changes have also been detected in *Helena* cellular expression patterns after interspecific hybridization. In parental species, *Helena* transcripts have been detected in mitotic spermatogonia, a stage characterized by a general high level of gene expression [42], where other TEs such as *copia* [43] and *412* [44] are expressed. In hybrids, however, we have detected *Helena* expression in later stages of spermatogenesis, including elongating spermatids (in F1) and mature sperm (in BC1 and BC2) cells, which are considered far less active transcriptionally. These results are in agreement with previous studies in hybrids of the same species, where *Osvaldo* expression was also found in the basal region of the testes [12]. This could suggest that transcriptional misregulation of *Helena* and other TEs occurs in hybrids, a phenomenon that has been described for some genes, and linked to hybrid sterility and other hybrid incompatibilities [45]. Concordantly, fertility recovery in hybrid males of *D*. *buzzatii* and *D*. *koepferae* takes place in some individuals from BC3 [46], where tissue expression patterns are very similar to the parents. Thus, incorrect localization of *Helena* expression might be involved in hybrid male sterility, since we know that sterility in our hybrids is caused by the additive effect of several minor loci [46]. However, our FISH results can only be interpreted qualitatively; *Helena* is mislocalized in hybrid testes, but its expression at a quantitative level decreases (in F1) or is maintained (BC1, BC2 and BC3) after interspecific hybridization (Fig 3C).

### Ovaries have additive values of *Helena* expression after interspecific hybridization

Ovaries are the only tissue where parental species expression differs significantly. *D*. *buzzatii* has higher *Helena* transcript levels than *D*. *koepferae* (Fig D in S1 File). The *Helena* copy number detected by Southern blot (S1 Fig) is higher in *D*. *buzzatii* (12–15) than in *D*. *koepferae* (6–12), but only 5 of the *D*. *buzzatii* copies are localized in the chromosome arms [11], less than in *D*. *koepferae* (12 copies). However, differences between their ERs are >10-fold (Fig 3D), which can only be explained if many *D*. *koepferae* copies are inactive. This hypothesis is in concordance with our *Helena* sequencing data, where the three sequenced *Helena* copies have truncated *pol* ORFs (Table 1).

In hybrid ovaries, *Helena* expression is at intermediate levels between *D*. *koepferae* and *D*. *buzzatii* for all generations (Fig 3D), but the amounts are always significantly higher than in *D*. *koepferae*. Since TE silencing in ovaries is crucial to maintain the genome integrity [29], organisms develop different strategies to efficiently control TE invasions. Thus, *Helena* ongoing neutralization might have followed different ways and reached different stages between our parental species. To explain *Helena* expression values, we focused on its regulation by piRNAs, which is the most important TE silencing mechanism in *Drosophila*.

We found that *D*. *buzzatii* has a larger *Helena*-specific population of piRNAs, whose ping-pong signature is higher than in *D*. *koepferae* (Fig 6). In F1 and BC1, the *Helena*-specific piRNA amounts are intermediate between those in the parental species (Fig 6A). However, secondary piRNA biogenesis seems to be less efficient in hybrid ovaries than in the parental species (Fig 6B), especially in F1, where there is a lower ping-pong signal in comparison to both *D*. *buzzatii* and *D*. *koepferae*. This reduced efficiency of ping-pong amplification may be due to a certain hybrid incompatibility in this pathway; indeed, even if our parental species are closely related, piRNA-mediated silencing effectors seem to be codified by rapidly evolving genes with positive selection marks [47], and whose expression varies widely between different populations of the same *Drosophila* species [48]. As suggested by results from testes, this malfunction might be compensated by the activation of the primary biogenesis pathway in order to maintain piRNA levels and preserve germline integrity.

At a cellular level, expression has been detected in nurse cells in all samples, but some generations of hybrid females (F1 and BC1) have a more widespread pattern of expression that also affects F1 follicular cells. Absence of a TE-specific piRNA in the mother cytoplasm can cause a transcriptional burst of this transposon in germ cells because maternally inherited piRNAs are responsible for TE silencing initiation at the first stages of development [49]. However, this putative increase of *Helena* expression in F1 hybrids compared with both parental species is not evident in our qRT-PCR results, which could be explained by the age of females, i.e. 3-day old for FISH and 10 for qRT-PCR. *Helena* expression rates might vary within a fly’s lifetime, as noted in P-M dysgenic crosses where fertility recovery in old females has been attributed to the regulation of P elements by paternally inherited piRNA clusters [50]. In our case, although *D*. *koepferae* cytoplasm contains *Helena*-specific piRNAs, their levels are lower than in *D*. *buzzatii* (Fig 6A). Thus, the maternal cytoplasm could indeed be less efficient in silencing *Helena* expression and might cause the extensive presence of *Helena* transcripts noted in F1 and BC1 ovaries of young females (Fig 5) [51]. Interestingly, there was an atypical *Helena* expression pattern including ovary follicular cells in F1 hybrids. A similar phenomenon has been described in *D*. *simulans*, where the failure of the maternal cytotype to repress the transposon *tirant* also involved its unusual expression in follicular cells [52], which could explain the presence of *Helena* transcripts in F1 follicle cells (Fig 5C). On the other hand, the lower efficiency of the ping-pong cycle in F1 hybrid ovaries (Fig 6B) could also be at the origin of widespread *Helena* expression. However, it is important to emphasise that FISH results are only qualitative, and that the generalized localization of *Helena* transcripts might not be linked to a real increase of expression.

We propose the following landscape in ovaries: a first stage of *Helena* enhanced expression would occur in young flies, because the ping-pong cycle seems to be less productive (especially in F1) than in parental species (Fig 6B), and also probably because *D*. *koepferae* cytoplasm is unable to efficiently silence *Helena* expression. Eventually, new *Helena* transposition events could take place at this time. This derepression step would be followed by the activation of other TE regulation mechanisms, such as primary piRNA biogenesis. This would allow *Helena*-specific piRNA levels to be maintained and would consequently decrease *Helena* expression. Therefore, our FISH experiments may be detecting transcripts that will later be post-transcriptionally silenced.

In conclusion, we have shown that interspecific hybridization modifies the expression of *Helena* retrotransposon in gonads. However, we demonstrate that *Helena* can be either transcriptionally repressed (as in F1 testes) or enhanced (as in F1 and BC1 young ovaries) in hybrids. Therefore, our study underlines the complexity of TE deregulation in hybrids, which not only differs between sexes but also presents different patterns between transposons [12]. The molecular understanding of the intricate mechanisms involved in TE silencing in hybrids might be crucial to cast light on the evolutionary role of TEs in phenomena such as hybrid sterility and speciation.

## Material and Methods

### *Drosophila* stocks and crosses

The strains used for interspecific crosses were (i) the Bu28 strain of *D*. *buzzatii*, an inbred line originated by the union of different populations (LN13, 19, 31 and 33) collected in 1982 in Los Negros (Bolivia); and (ii) the Ko2 strain of *D*. *koepferae*, an inbred line originated from a population collected in 1979 in San Luis (Argentina). Both lines were maintained by brother-sister mating for over a decade and are now kept by mass culturing.

Because hybrid egg viability between *D*. *buzzatii* and *D*. *koepferae* is low [53] and the hybrid offspring scarce, we performed 14 different crosses for qPCR experiments, denoted as families A through N. For each family, 10–15 Ko2 virgin females were crossed with 10–15 Bu28 males of the same age. This cross was followed by three generations of backcrossing of 10–15 hybrid females (whenever possible) with the same number of *D*. *buzzatii* males. Five additional crosses (as just described) were used for FISH analyses. All stocks and crosses were reared at 25°C in a standard *Drosophila* medium supplemented with yeast.

### *Helena* molecular characterization

PCR reactions were carried out in a final volume of 50 μl, including 1× High Yield Reaction Buffer with Mg^2+^ (Kapa Biosystems), 0.2 mM of each dNTP (Roche), 0.4 μM of each primer (Sigma-Aldrich), template DNA (≈10–20 ng) and 0.04 U/μl of Taq polymerase (KapaTaq from Kapa Biosystems). A MJ Research Inc. thermocycler was used, with the following program: 5 min at 94°C (preliminary denaturation); 30 cycles of 45 s at 94°C (denaturation), 45 s at specific PCR annealing temperatures and 1 min 30 s at 72°C (extension); and 10 min at 72°C (final extension). The two longest copies of *Helena* from *D*. *koepferae* were amplified using Roche’s Expand Long Template PCR system. Amplified samples were stored at 4°C, gel purified with the Nucleospin Gel and PCR Clean-Up kit (Macherey-Nagel), and cloned with the pGEM-T Easy Vector System I (Promega).

Primers were designed from the longest copy of *Helena* from the *D*. *mojavensis* genome [17], the closest sequenced species to *D*. *buzzatii* and *D*. *koepferae*: HelMojF2A (5’-AGCAGCCCAGAAAATGCTTA-3’) and HelMojR2B (5’-TCTCAGCGGTAAGGTGCTCT-3’). For the *Helena* shortest copy from *D*. *koepferae*, HelMojR1A (5’-GTCCACAACCACAACCACAG-3’) was used instead of HelMojR2B.

*Helena* isolated clones were sequenced using capillary sequencing technique (Macrogen Inc); they were analyzed using different NCBI tools and databases, such as nucleotide BLAST [54] (megablast algorithm, for highly similar sequences), ORF Finder and Conserved Domain Search [25]. Sequence data from this article have been deposited in GenBank repository (accession numbers KF280391, KP115213, KP115214 and KP115215).

For the phylogenetic analysis, we used some of the *Helena* consensus sequences identified in the 12 *Drosophila* genomes: from *D*. *sechellia*, *D*. *yakuba*, *D*. *erecta*, *D*. *ananassae*, *D*. *mojavensis* [17] and *D*. *simulans* [18], as well as other *Helena* characterized sequences from *D*. *virilis* (Repbase ID = HELENA) [16] and *D*. *melanogaster* (accession number AF012030) [28]. The retrieved sequences, together with *D*. *buzzatii* and *D*. *koepferae* obtained copies, were aligned using MAFFT 7.123b E-INS-i algorithm, optimized for sequences with multiple conserved domains and long gaps [55]. The alignment was automatically cleaned using Gblocks [56] web server, allowing smaller final blocks, gap positions within the final blocks and less strict flanking positions (S1 Text for the fasta alignment). A graphical representation of the final alignment using ESPript [57] (http://espript.ibcp.fr) is also available (S2 Text). Maximum likelihood (ML) phylogenetic trees were estimated by RaxML 7.2.8 [58], using the GTRCAT model. Statistical support for bipartitions was estimated from 100-bootstrap replicates with RaxML (same model).

### Southern blot

Genomic DNA from *D*. *buzzatii* and *D*. *koepferae* (Bu28 and Ko2 strains, respectively) was extracted from a pool of 30 flies according to [59]. The DNA was digested with *AatII* (Roche), which has no restriction sites within the *Helena* sequence, allowing us to estimate the number of complete copies. After agarose gel electrophoresis and denaturing steps, the DNA was transferred to a positively charged nylon membrane (Roche). Pre-hybridization and hybridization steps were carried out at 42°C in a solution containing 5x SSC, 50% of formamide, 0.1% of *N*-laurylsarcosine, 0.02% of SDS and 5% of blocking reagent (Roche). The membrane was hybridized with a dig-labelled DNA probe of ≈3.8 kb, corresponding to the longest sequenced fragment of *Helena* in *D*. *buzzatii*.

### Quantification of *Helena* transcripts by RT-PCR

Four types of samples were analyzed for each generation: ovaries, testes, female somatic carcasses and male somatic carcasses. Flies were dissected in PBT (1× phosphate-buffered saline [PBS], 0.2% Tween 20) 10 days after their birth, to ensure a sufficient number of offspring for analysis. Total RNA was purified from >10 ovaries, 14 testes or 10 carcasses per sample with the RNeasy kit (Qiagen) and then treated with DNaseI (Ambion). cDNA synthesis was carried out with anchored-oligo(dT)_18_ primers using Roche’s Transcriptor First Strand cDNA Synthesis Kit. Transcript abundance was estimated by fluorescence intensity using Biorad’s iQ SYBR Green Supermix on a CFX96 BioRad Real-Time lightcycler with primers specific to the *Helena* endonuclease region. Relative quantification was performed using the ribosomal *rp49* housekeeping gene, which is equally expressed in *D*.*buzzatii* and *D*. *koepferae*, as an endogenous control for the standard curve method. Two technical replicates were run for each sample. ΔC_T_ values for all samples are summarized in S3 Text and have been used to calculate expression rates as in [27].

Primers used to amplify *Helena* were designed from a fragment of *Helena* characterized earlier from the same hybrids [11]. The qPCR fragment corresponds to 200 bp of the endonuclease region amplified with the following primers: HelenaF1 (5’-CGACATACTCGCTTCCTGTG-3’) and HelenaR1 (5’-TCACACTCCCTCTTGCATTG-3’). For *rp49*, the published primers [60] designed from *D*. *mojavensis* genome were used, that give a qPCR amplicon of 196 bp. The primer efficiencies were 96.6 and 99% for *Helena* and *rp49* respectively.

### Fluorescent *in situ* hybridization in ovaries and testes

We dissected the ovaries and testes of 3-days old flies in PBT, which is the ideal age for optimal visualization of the different cells from ovaries. We followed the protocol described in [61]. The *Helena* antisense RNA probe was a 984-pb fragment corresponding to the *pol-like* gene (primers HelenaF1 and HelMojR1A), which included T7 and SP6 promoter sites. It was labelled by *in vitro* transcription of SP6/T7 using DIG RNA Labelling Kit (Roche). Labelled probes were detected using anti-DIG POD antibody (Roche) and fluorescence amplification (TSA PLUS Cyanine3 kit, PerkinElmer), visualized with a TCS-SP5 Leica confocal scanning laser microscope.

### piRNA analyses: small RNA extraction, library preparation, sequencing and alignment

We dissected 5 to 6-days old flies as described above. Small RNA was purified from ovaries (n = 70 pairs for all samples) and testes (n = 96 pairs for *D*. *buzzatii* and n = 333 pairs for F1 sterile males), following the manual small RNA purifying protocol described of Grentzinger *et al*. [62]. After small RNA isolation, samples were gel-purified and precipitated. A single Illumina library was prepared for each sample and sequenced on an Illumina Hiseq 2500 platform by FASTERIS SA (Switzerland). Reads of 23–32 nucleotides were selected as piRNAs and trimmed using UrQt [63] to remove low-quality nucleotides. The trimmed reads were aligned to the *D*. *buzzatii* genome TE library [32] using Bowtie1 v1.1.1 [64] (the most sensitive option and keeping a single alignment for reads mapping to multiple positions). The read count step (built in TE tools: https://github.com/l-modolo/TEtools) was computed per TE family by adding all reads mapped on copies from the same family. Finally, read counts were normalized using the R Bioconductor package DESeq2 [65]. Only the results for *Helena* retrotransposon were used for this study.

Ping-pong signature was analyzed by checking the presence of sense-antisense read pairs overlapping by 10 nucleotides, using Antoniewski's signature.py pipeline [66]. For this analysis, we used the raw 23–32 nucleotide reads since a trimming step would bias the real small RNA length aligned to the *Helena* sequences of the same TE library (as described above).

### Statistical methods

R software was used for statistical analyses. Because the assumptions of Gaussian distribution and equal variances are not valid in qRT-PCR experiments with small sample sizes, the most suitable test is the robust non-parametric Wilcoxon rank sum test (also called the Mann-Whitney test [67]), which was used to compare expression rates of hybrids and parental species at each generation. Kruskal-Wallis test [68] was used to determine whether differences between all groups (including all parents and hybrids) were significant. Finally, Levene’s test for equality of variances was used to assess changes in variance between groups.

## Supporting Information

S1 Fig Southern blot analysis of *Helena* in parental species, *D*. *buzzatii* (left) and *D*. *koepferae* (right). No restriction sites for *AatII* are present in *Helena*’s probe sequence. Thus, digestions with this enzyme allow us to distinguish different *Helena* copies. Arrows in red indicate strong-signaled bands; arrows in black indicate faint bands.
(TIFF)

S1 File *Helena* expression results in parental species. **(Fig A and B)** *Helena* expression rates relative to *rp49* housekeeping gene in *D*. *koepferae* (Dko) and *D*. *buzzatii* (Dbu) somatic tissues **(A)** and gonads **(B)**. Male samples are represented in blue and female samples are represented in brown. Boxes are determined by the first and third quartile values, with an intermediate deep line corresponding to the median value. Circles correspond to outliers (above or below 1.5-fold the interquartile range), and triangles represent those outliers whose ERs are extremely outranged and cannot be represented in the same scale (triangle in A: ER = 2.9×10^−3^, in B: ER = 3.6×10^−3^ and 6.2×10^−3^). **(Fig C and D)** Comparison of *Helena* expression rates between all different parental samples for somatic tissues **(C)** and gonads **(D)**. N = number of replicates analyzed, SD = standard deviation, W = Wilcoxon rank sum test statistic, p-value = probability. *: p-value < 0.05, **: p-value < 0.01, ***: p-value < 0.001. In red, p-values that are significant after Bonferoni correction (p-value<0.008).
(PDF)

S2 File FISH of *Helena* RNA expression in different F1 hybrid testes. Red staining are *Helena* transcripts, green staining is tissue autofluorescence. Arrows mark the presence of *Helena* transcripts.
(TIFF)

S3 File FISH of *Helena* RNA expression in different BC1 hybrid testes. Red staining are *Helena* transcripts, green staining is tissue autofluorescence. Arrows mark the presence of *Helena* transcripts.
(TIFF)

S4 File FISH of *Helena* RNA expression in different BC2 hybrid testes. Red staining are *Helena* transcripts, green staining is tissue autofluorescence. Arrows mark the presence of *Helena* transcripts.
(TIFF)

S1 Table Summary of BLAST alignment results between *Helena* sequenced copies. *Dbu* = *D*. *buzzatii*, *Dko28 = D*. *koepferae*-28, *Dko35-1* = *D*. *koepferae*-35-1, *Dko35-2* = *D*. *koepferae*-35-2.
(PDF)

S2 Table Variance comparisons of *Helena* expression rates between each hybrid generation and parental species. W = Levene’s test for equality of variances satistic, p-value = probability. *: p-value<0.05, **: p-value<0.01, ***: p-value<0.001. In red, p-values that are significant after Bonferoni correction (p-value<0.01). Each kind of sample (males, females, testes, ovaries) has been compared to the same tissue of both parental species.
(PDF)

S1 Text Alignment of *Helena* sequences (in fasta format) obtained with MAFFT E-INS-i algorithm and cleaned using Gblocks. This alignment was used to construct the phylogenetic tree on Fig 2.
(PDF)

S2 Text Graphical representation of the *Helena* alignment obtained with MAFFT E-INS-i algorithm and cleaned using Gblocks. Highly conserved residues (similarity score per position > 0.5) are framed in blue and used to build the consensus sequence. Each nitrogenous base in a conserved position is represented in a different colour.
(PDF)

S3 Text Summary of ΔC_T_ values for all studied replicates (from different crosses) of each kind of sample for all generations. (PDF)
