# Supplementary figures and images for: Expression of the Retrotransposon Helena Reveals a Complex Pattern of TE Deregulation in Drosophila Hybrids
Source: PLoS One. 2016 Jan 26;11(1):e0147903. doi: 10.1371/journal.pone.0147903 (PMC4728067; doi:10.1371/journal.pone.0147903)

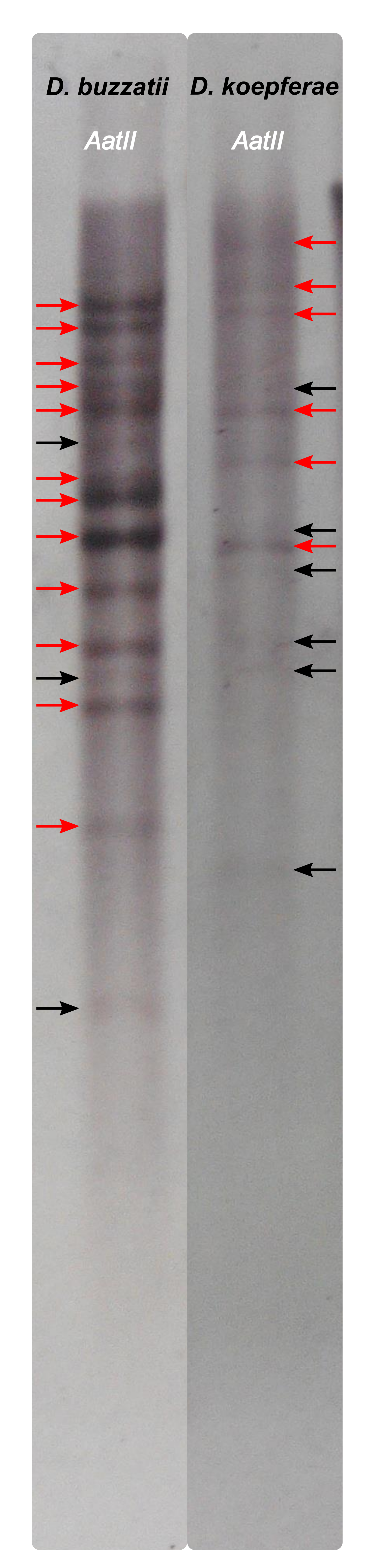

Supplement: S1 Fig — No restriction sites for AatII are present in Helena’s probe sequence. Thus, digestions with this enzyme allow us to distinguish different Helena copies. Arrows in red indicate strong-signaled bands; arrows in black indicate faint bands. (TIFF) [file pone.0147903.s001.tiff]

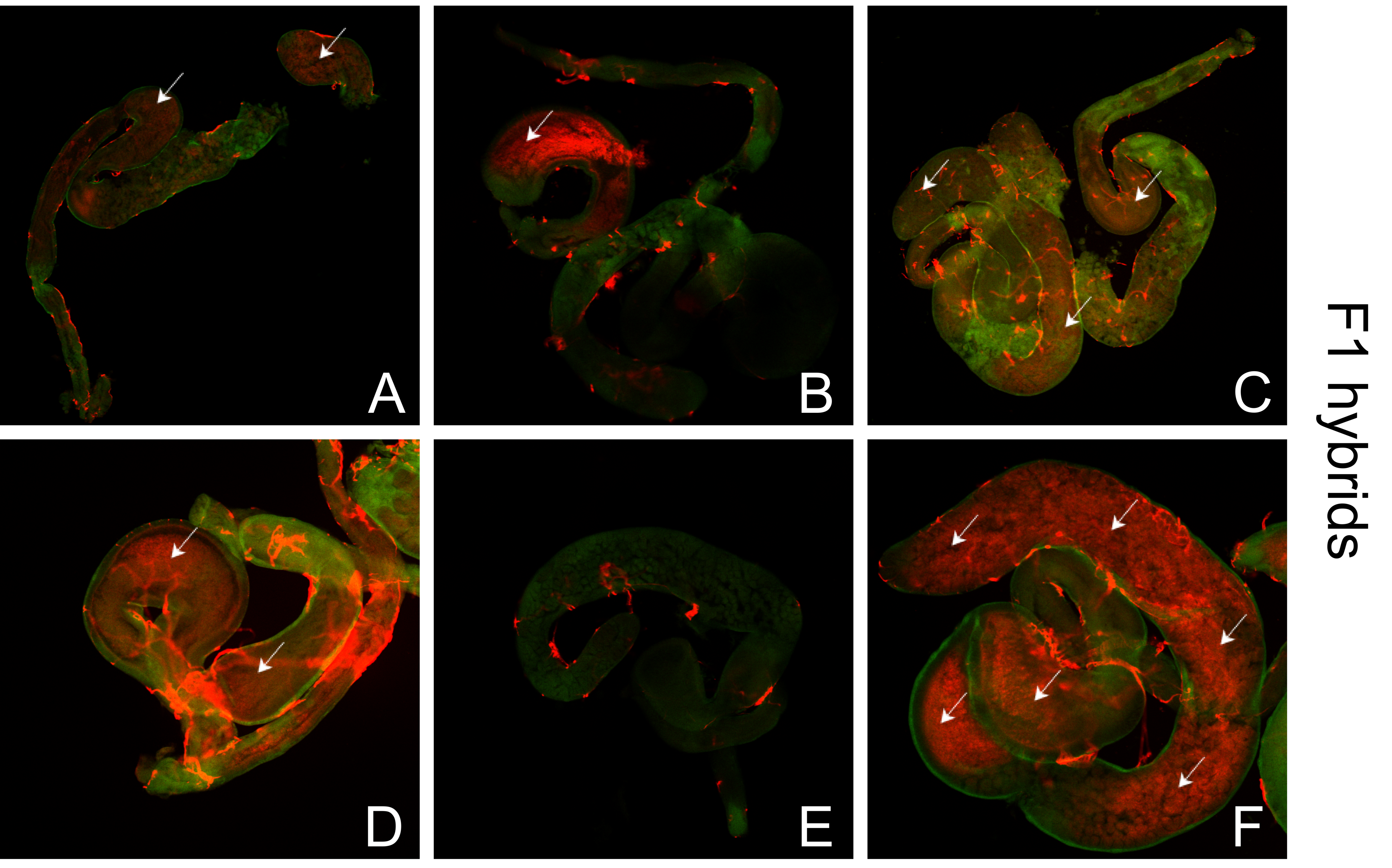

Supplement: S2 File — Red staining are Helena transcripts, green staining is tissue autofluorescence. Arrows mark the presence of Helena transcripts. (TIFF) [file pone.0147903.s003.tiff]

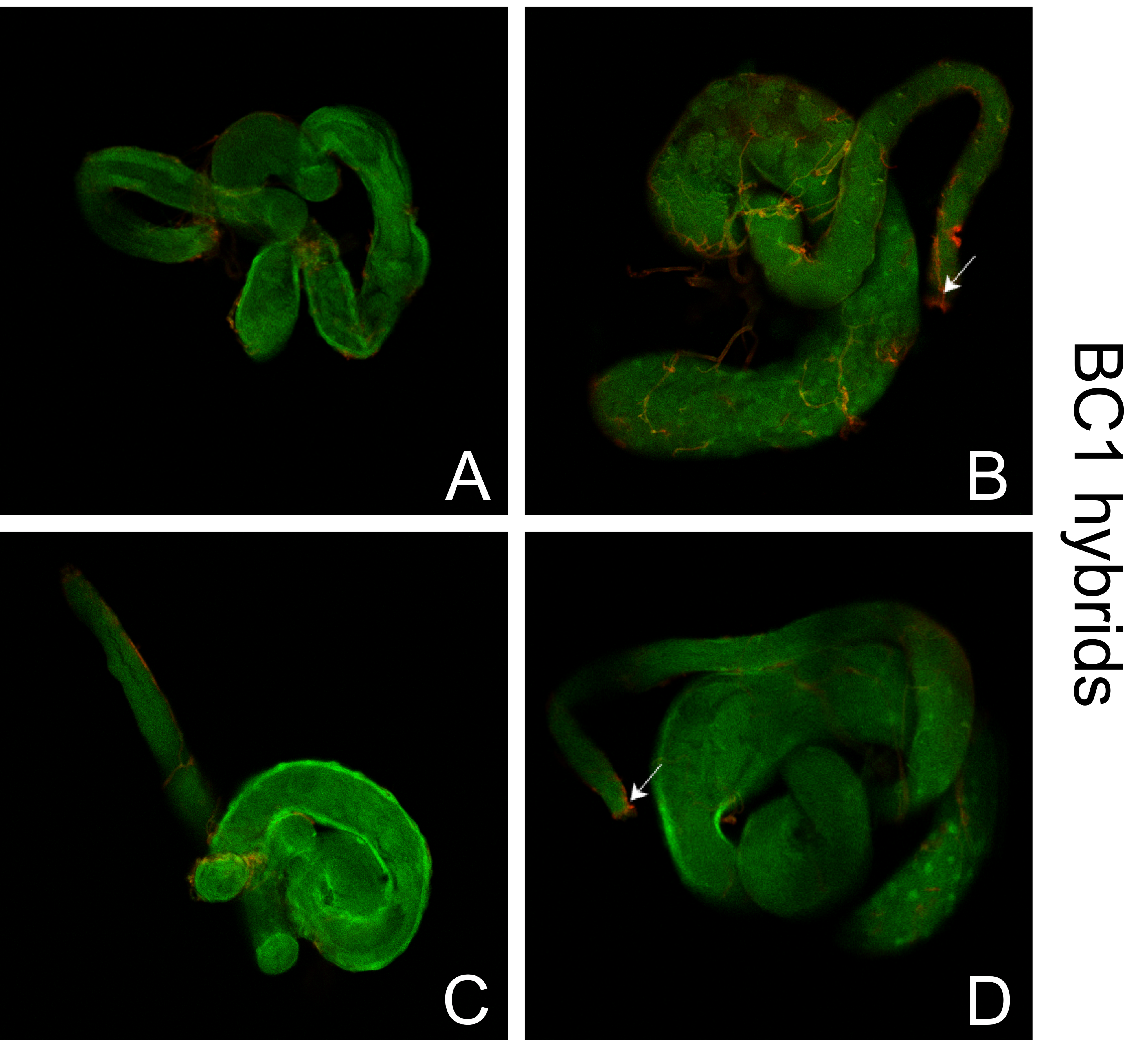

Supplement: S3 File — Red staining are Helena transcripts, green staining is tissue autofluorescence. Arrows mark the presence of Helena transcripts. (TIFF) [file pone.0147903.s004.tiff]

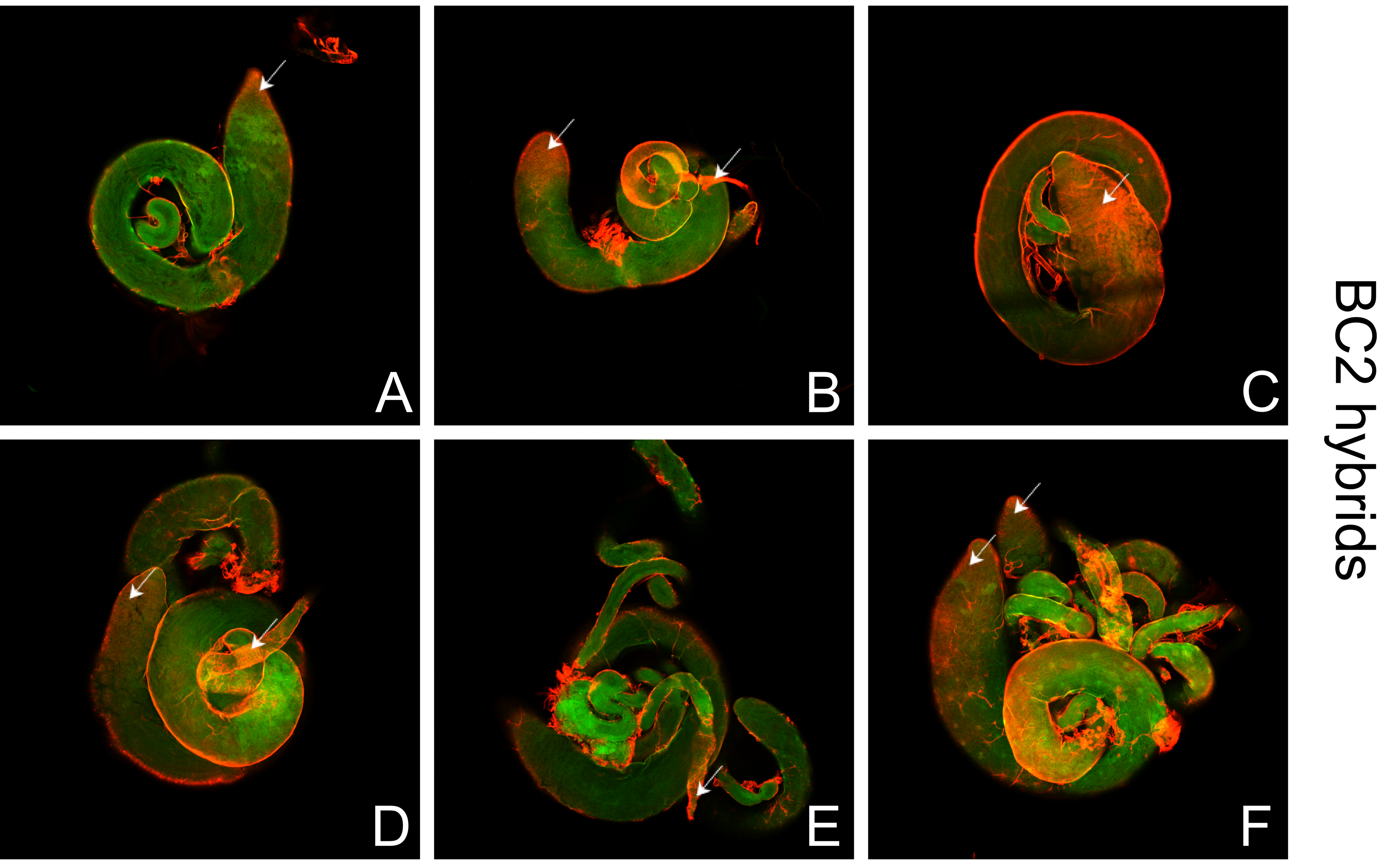

Supplement: S4 File — Red staining are Helena transcripts, green staining is tissue autofluorescence. Arrows mark the presence of Helena transcripts. (TIFF) [file pone.0147903.s005.tiff]
