# Supplementary material for: Expression of the Retrotransposon Helena Reveals a Complex Pattern of TE Deregulation in Drosophila Hybrids
Source: PLoS One. 2016 Jan 26;11(1):e0147903. doi: 10.1371/journal.pone.0147903 (PMC4728067; doi:10.1371/journal.pone.0147903)
Supplement: S1 File — (Fig A and B) Helena expression rates relative to rp49 housekeeping gene in D. koepferae (Dko) and D. buzzatii (Dbu) somatic tissues (A) and gonads (B). Male samples are represented in blue and female samples are represented in brown. Boxes are determined by the first and third quartile values, with an intermediate deep line corresponding to the median value. Circles correspond to outliers (above or below 1.5-fold the interquartile range), and triangles represent those outliers whose ERs are extremely outranged and cannot be represented in the same scale (triangle in A: ER = 2.9×10^−3^, in B: ER = 3.6×10^−3^ and 6.2×10^−3^). (Fig C and D) Comparison of Helena expression rates between all different parental samples for somatic tissues (C) and gonads (D). N = number of replicates analyzed, SD = standard deviation, W = Wilcoxon rank sum test statistic, p-value = probability. *: p-value < 0.05, **: p-value < 0.01, ***: p-value < 0.001. In red, p-values that are significant after Bonferoni correction (p-value<0.008). (PDF) [file pone.0147903.s002.pdf]

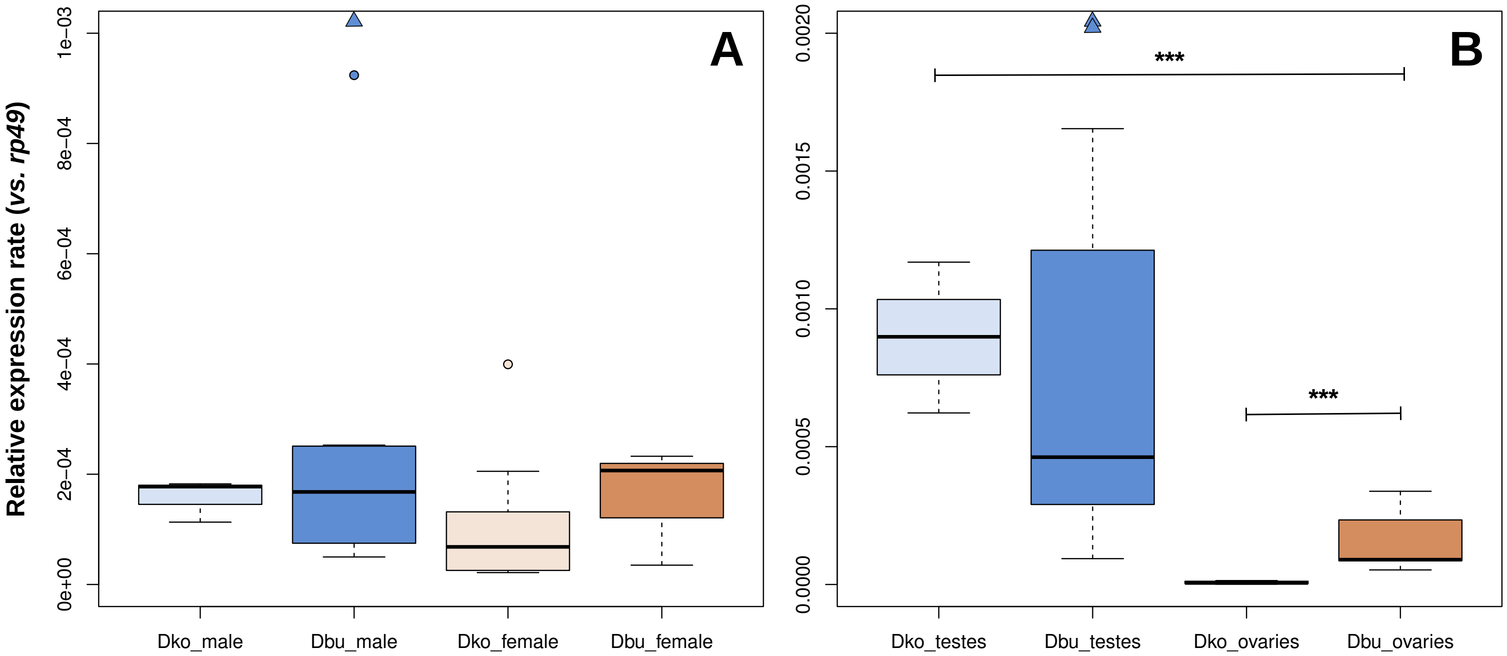

C

					males				females			
					D. buzzatii		***D. koepferae***		***D. buzzatii***		***D. koepferae***	
		**N**	**median**	**SD**	**W**	**p-value**	**W**	**p-value**	**W**	**p-value**	**W**	**p-value**
**males**	***D. buzzatii***	11	1.68E-04	8.40E-04			18	8.85E-01	14	7.69E-01	31	1.75E-01
	D. koepferae	3	1.77E-04	3.87E-05					6	7.00E-01	7	2.82E-01
**females**	***D. buzzatii***	3	2.07E-04	1.07E-04							8	3.73E-01
	D. koepferae	9	6.82E-05	1.22E-04								

D

					testes				ovaries			
					D. buzzatii		***D. koepferae***		***D. buzzatii***		***D. koepferae***	
		**N**	**median**	**SD**	**W**	**p-value**	**W**	**p-value**	**W**	**p-value**	**W**	**p-value**
**testes**	***D. buzzatii***	11	4.62E-04	1.91E-03			23	3.68E-01	5	8.70E-03**	0	5.67e-06***
	D. koepferae	3	8.99E-04	2.74E-04					0	3.57E-02*	0	6.99E-03**
**ovaries**	***D. buzzatii***	5	9.00E-05	1.22E-04							0	6.66E-04***
	D. koepferae	10	5.50E-06	4.80E-06								
