# Supplementary material for: Expression of the Retrotransposon Helena Reveals a Complex Pattern of TE Deregulation in Drosophila Hybrids
Source: PLoS One. 2016 Jan 26;11(1):e0147903. doi: 10.1371/journal.pone.0147903 (PMC4728067; doi:10.1371/journal.pone.0147903)
Supplement: S1 Table — Dbu = D. buzzatii, Dko28 = D. koepferae-28, Dko35-1 = D. koepferae-35-1, Dko35-2 = D. koepferae-35-2. (PDF) [file pone.0147903.s006.pdf]

Table S1: **Summary of BLAST alignment results between *Helena* sequenced copies.** *Dbu* = *D. buzzatii*, *Dko28* = *D. koepferae*-28, *Dko35-1* = *D. koepferae*-35-1, *Dko35-2* = *D. koepferae*-35-2.

Species	Sequence Identity	E-value	Max Score
Dbu* vs. *Dko28	98%	0.0	4823
Dbu* vs. *Dko35-1	89%	0.0	3472
Dbu* vs. *Dko35-2	97%	0.0	4275
Dko28* vs. *Dko35-1	90%	0.0	3517
Dko28* vs. *Dko35-2	98%	0.0	4405
Dko35-1* vs. *Dko35-2	93%	0.0	4848
