# Supplementary material for: Expression of the Retrotransposon Helena Reveals a Complex Pattern of TE Deregulation in Drosophila Hybrids
Source: PLoS One. 2016 Jan 26;11(1):e0147903. doi: 10.1371/journal.pone.0147903 (PMC4728067; doi:10.1371/journal.pone.0147903)
Supplement: S2 Table — W = Levene’s test for equality of variances satistic, p-value = probability. *: p-value<0.05, **: p-value<0.01, ***: p-value<0.001. In red, p-values that are significant after Bonferoni correction (p-value<0.01). Each kind of sample (males, females, testes, ovaries) has been compared to the same tissue of both parental species. (PDF) [file pone.0147903.s007.pdf]

S2 Table: Variance comparisons of *Helena* expression rates between each hybrid generation and parental species. W= Levene's test for equality of variances statistic, p-value= probability. *: p-value < 0.05, **: p-value < 0.01, ***: p-value < 0.001. In red, p-values that are significant after adjustment by Bonferroni correction (p-value<0.01). Each kind of sample (males, females, testes, ovaries) has been compared to the same tissue of both parental species.

		variance	vs. *D. buzzatii*		vs. *D. koepferae*	
			W	p-value	W	p-value
males	***D. koepferae***	1.50E-09	1.72	2.14E-01	-	-
	D. buzzatii	7.06E-07	-	-	1.72	2.14E-01
	**F1**	3.28E-04	11.06	**4.61E-03****	2.90	1.32E-01
	**BC1**	2.26E-07	1.41	2.50E-01	0.97	3.44E-01
	**BC2**	1.66E-06	0.27	6.06E-01	1.24	2.89E-01
	**BC3**	6.47E-08	2.52	1.30E-01	2.12	1.76E-01
females	***D. koepferae***	1.49E-08	1.56	1.89E-01	-	-
	D. buzzatii	1.15E-08	-	-	1.56	1.89E-01
	**F1**	3.70E-08	0.80	3.91E-01	1.53	2.33E-01
	**BC1**	7.20E-09	0.47	5.05E-01	0.99	3.32E-01
	**BC2**	5.34E-09	0.98	3.41E-01	1.51	2.33E-01
	**BC3**	4.01E-08	0.19	6.73E-01	0.38	5.44E-01
testes	***D. koepferae***	7.48E-08	2.43	1.45E-01	-	-
	D. buzzatii	3.64E-06	-	-	2.43	1.45E-01
	**F1**	7.21E-09	5.06	**4.11E-02***	2.85	1.42E-01
	**BC1**	1.24E-07	7.64	**1.20E-02***	0.99	3.40E-01
	**BC2**	3.54E-07	6.71	**1.63E-02***	1.17	2.96E-01
	**BC3**	6.53E-08	8.13	**1.02E-02***	0.08	7.89E-01
ovaries	***D. koepferae***	2.30E-11	48.20	**1.02E-05*****	-	-
	D. buzzatii	1.48E-08	-	-	48.20	**1.02E-05*****
	**F1**	1.70E-09	23.45	**2.26E-06*****	9.83	**5.21E-03****
	**BC1**	1.04E-09	27.03	**8.79E-05*****	17.18	**4.60E-04*****
	**BC2**	7.02E-10	33.03	**3.00E-05*****	11.52	**2.73E-03****
	**BC3**	5.97E-06	1.65	2.18E-01	3.98	6.00E-02
