# Supplementary material for: Expression of the Retrotransposon Helena Reveals a Complex Pattern of TE Deregulation in Drosophila Hybrids
Source: PLoS One. 2016 Jan 26;11(1):e0147903. doi: 10.1371/journal.pone.0147903 (PMC4728067; doi:10.1371/journal.pone.0147903)
Supplement: S1 Text — This alignment was used to construct the phylogenetic tree on Fig 2. (PDF) [file pone.0147903.s008.pdf]

>Dsechellia
gatgcgcaaa aaacgaaaga gaggaagagg aagaccaatt taacacccaa ccatagctct
aaacgagctg tcagagacgc gccatatcca gccataaata gtaattcaaa ctcaaacaat
aggtttgcca tgctagacat ggaattggac gaaaccagtg atggcataga ttgtcatacc
tcgtgtgcat cggctgctaa tgatgactgt gtaccaaatg tgacatccaa tagcccacaa
cagtatac-- ----tgataa gcagaattca aaaccaccgc aaatagtact gagccttacc
aatcttaatg atctctatga gctcattacg gaggtcacta gcctagataa tttaacagtt
aaagtcaatc aaggggaaac agtgagaata ttacccaaag actctgatac ttacagagcc
attattaata tttttgataa ttcgggaatt gaattccata cgtaccaaat gaaggaagag
aagcctcaca gaatagttgt ta-------- ---------a ccctaaccta cgaaattatc
gacaacttta aaaatatggc tttgatgttc tacaagtaca caacccaaga tccaggagaa
atagagaaga aaaacttaat atattcttca ttaatataaa cccttgtgca aaaattaatg
acatatacga tattaacaca gtatgccgac agaaagtgcg gatagaaaga atgcgtaaat
catctgaaat tgctcaatgc atacgttgtc aggaattcgg ccacacagct aaatactgtc
gtcgtcatcc caactgtgct cgatgtggtg aaaatcactt aacaagctat gcgtacttcc
caatgatcaa cagcctatct gtatacactg tggaggaaat cacacggcaa gttacaaggg
ttgccagttt taccaggagt atcttcgacg atcattgggc actgtaaaga caggtttgat
aaacaaacca acaacaaccc cagcaaaaac ---------- ---agcagca tcaatagcta
gcactcccaa aggcttgtcc tacgcagata ttgcaaaaaa tggcaataca acagcccagc
ctcgtctaca taatgtac-- ---------- ---------a attaaaggga actaatatta
agcagcaaca cccgcttgac gttcagtcaa tattggcaca gcaacaggaa caatttatga
agtggcagca acagcttcaa cagcaacaac agcagcaatt cctatcgtgg ctactacagc
agcaacagga gcaacaacaa cacaacaagt tgaatagtca acgactcgaa aggctggaaa
aaattgtttt tgaaatggcc aatatgctga agcaacggac tggggataca tcggctcccc
aactccatag taacgattca ccatcgcaat taaccctctg aagattctta tctggaatgt
aaatggtttt tcaggtaaag ccagagaagt cgagctcttc gcacacaaca acggcattga
cattcttctc ctaaacgaga tcagactcaa cagagggaac acagttaaga tatatgggta
tagcttttat cccgcataca aaccttcaag ccataatcac ggaatgggag gagcagcagt
actggtgaga agctctcttc gtcatttccc gcaaagagtt attgaaacga gaactattca
gatgtcttca gtcaaggtct ccaccgggct gggagatatg gaatttagcg cgatttactg
tccaccaaca aatagaattg aggaaaggca cttcagtgac atacttgtct cttgtggaca
aaggtatttc gttggtgggg actgaaacgc ccgatattgg ctatggggtg atacgtacaa
ttaacccaga ggtcgagaac tagcagaagc catttcagcc agaggtgctt atatccttgc
aacaggttca ccaactagat acccacatgt gcccagtcac agacctacct gcattgattt
tgctgtgtac catgggataa acttagacag aactagtatt tctgaaaatt gggatctaga
ctccgatcat gtagcccttg tggctactgt acaaacagaa ggtgcctatg ttagaccatg
ctctcggtta ataaacagcc gaactgatct ccttgttttc agacaacatc tggaaaactc
tctccaatta aatacggttc tgagctctaa ggaagatatc gagaacgcag tcgcagtcta
acgcaaaata tatatatagc cgcttctgct tctac----- ----gcccga gatacgcccc
aagttatggt attgttctaa caagagaggc agataactta tcagaactaa gagacgcctt
cgaagaagag caattcgaac tcaggatcca tgggacagaa tcttgtggaa ccgagcagca
aagcaactca aagtcatctt aagggaactc agaagtgatt tctttgagca aaaattatcc
tccatggact acaccgttga tgcaaactat tcgctgtgga agtgcacaaa agcgcttaag
cgacaaccac ttcgatgggt acccgtacgc tgtccaggtg gggaatttgc aaaaagaagt
ggaacaggct aatgcattcg gcttccacct agagtatcgc ttcactcctt acgacttcgc
cacgacagaa caaataagag agactcacca gtacctacaa atgccattgc agatgtcttg
gcctattaag ccaataagga tagaagaaat ccttgaaata attaaattac tgccgaagca
taaagcatct ttaatgctat tttaaggatc caagtgttcc caagacagtg gaaaatgcct
gctattttga tgatccacaa gcctggaaag ccggaagatg atccagagtc gtatcggcct
ataagcctct taccctcact ttctaaatta tgggagagac ttattgccaa tcggataaac
gacattataa gacaaggcaa tatcttgccg gatcatcaat ttggatttcg aaagggacac
ggaactattg aacaggtcca cagactggtg aaacacatac tacaggcttt tgacgactgc
gagtactcca acgccgtctt tatagatatg caacaagcct tcgacaaagt atggcatgtt
ggattattat gcaagataaa gacccttcta cctgcgccct acttctgtat tttaaagtca
tatctagaag aaagagaatt taaaatcacg gtgagaaata gctactcctc tatataccca
atgagagctg gagtccctca gggcagtgtt ctcggaccgc tactgtattc cttgtacact
gctgatatcc cttgcccgag tttcgaacac atggcagcac cgaacaggac tcttattgca
acctatgcag atgacatcgc agttgtatat aactctaggg acagcagaga gacagctaac
ggactacaag aatatattaa tgatctggca gcctggtgta aacggtggaa cctaaaaata
aacccactga aaacaacaaa cccgtgcttc acgttaaaaa cgcttatccc gaacacccct
ccaattcggc tagaaggagt taccctgaat cagaccctgc aagcaacaca tctaggtatc
accctggatt aacggctcac ctttgggccg catctaaaaa aaacagtaaa aaaatgtggc
cacagattac aacagctgag atggcacatg aatagaagga gcactctttc gatgaggtgc
aaaagagctg tgtatgcgca ctgtatcgta ccgatatggt tatacgggat ccagatttga

```
ggaattgcag ccaaatcgaa ttataatcgt atccaggtct gcccctggta tgtacctaac
tctacactcc ataaagacct caatattcac acagttgagt cacaaattgg gagacataca
tgtcgatata gtgacagatt actgagccat agcagtcttc ttgcaagacg tctcatcccc
gctcgacctc t
>Dyakuba
---------- ---------- ---------- ---------- ---------- ----------
---------- ---------- ---------- ---------- ---------- ----------
---------- ---------- ---------- ---------- ---------- ----------
---------- ---------- ---------- ---------- ---------- ----------
---------- ---------- ---------- ---------- ---------- ----------
---------- ---------- ---------- ---------- ---------- ----------
---------- ---------- ---------- ---------- ---------- ----------
---------- ---------- ---------- ---------- ---------- ----------
---------- ---------- ---------- ---------- ---------- ----------
---------- ---------- ---------- ---------- ---------- ----------
---------- ---------- ---------- ---------- ---------- ----------
---------- ---------- ---------- ---------- ---------- ----------
---------- ---------- ---------- ---------- ---------- ----------
---------- ---------- ---------- ---------- ---------- ----------
---------- ---------- ---------- ---------- ---------- ----------
---------- ---------- ---------- ---------- ---------- ----------
---------- ---------- ---------- ---------- ---------- ----------
---------- ---------- ---------- ---------- ---------- ----------
---------- ---------- ---------- ---------- ---------- ----------
---------- ---------- ---------- ---------- ---------- ----------
---------- ---------- ---------- ---------- ---------- ----------
---------- ---------- ---------- ---------- ---------- ----------
---------- ---------- ---------- ---------- ---------- ----------
---------- ---------- ---------- ---------- ---------- ----------
---------- ---------- ---------- ---------- ---------- ----------
---------- ---------- ---------- ---------- ---------- ----------
---------- ---------- ---------- ---------- ---------- ----------
---------- ---------- ---------- ---------- ---------- ----------
---------- ---------- ---------- ---------- ---------- ----------
---------- ---------- ---------- ---------- ---------- ----------
---------- ---------- ---------- ---------- ---------- ----------
---------- ---------- ---------- ---------- ---------- ----------
---------- ---------- ---------- ---------- ---------- ----------
---------- ---------- ---------- ---------- ---------- ----------
---------- ---------- ---------- ---------- ---------- ----------
---------- ---------- ---------- ---------- ---------- ----------
---------- ---------- ---------- ---------- ---------- ----------
---------- ---------- ---------- ---------- ---------- ----------
---------- ---------- ---------- ---------- ---------- ----------
---------- ---------- -------cca tgggacagaa tcttgtggaa ccgagcagca
aagcaactga aaggcattct aagggagctc agaaatgatt tctttgagca aaaattatct
tccatggact acacagttga tgcaaactat tcgctgtgga agtgcacaaa agcgctcaag
agacaaccat ttcgatgggt accagtacga tgtcctggcg ggcaaattgc aaaaagaagt
ggaacaggct aatgcattcg gctaccacct tgaggatcat ttcactcctt atgacttcgc
cacgacagag cagataagtg agactcacca gtacctacaa atgccattgc agatgtcttt
gcctattaag ccaataagaa tagaagaaat atctgaagta attaaatcac tgccgaaaca
taaagcatct ttaacgctat tttaagggtc caagtattcc caagacagtg gaaaatggct
gttatcatga tgatccacaa acccgggaaa ccagaagctg atcctgagtc ttatcggcct
ataagcctgt taccctccct ctctaaatta tgggaaagac ttcttgtcaa aaggatcaat
gacattgtaa gacaaggtta tatcttgccg gaccatcaat ttggatttcg gaagggacac
ggcactgtag aacaggtcca cagactggtg aagcacatat tacaggcttt tgacgactgc
gaatacgcaa acgctgtctt tatagatatg caacaagcct tcgacaaagt atggcatgtt
ggattattat gcaagataaa gaccatgctt cctgcgccct acttctgtat tttaaagtca
tatctagagg gacgagaatt taaaatcaca gttaggaata actactctac cgtatatcca
atgagagctg gagtcccaca gggcagcgtc cttggaccac tactatactc cttgtacact
gcagatatcc cttgcccgaa cttcgatcac atggaagcac cctacaaggc tcttatggca
acctatgcag atgacatcgc agtggtgtat aactctgggg acagcggaga ggcagcgaaa
ggactgcaag aatacattaa tgctctagca gcctggtgta aacggtggaa tctaaaaata
aatgcaacga aaaccacaaa tctatgtttc acattgaaaa cgctcatcaa aaaaacaccc
```

ccactccagc tagaaggagt tacactagac cagccgctgc aagcaacata tctaggtata
acactggata aacgcctcac ctttgggccg catctcaaaa atgcagtaaa gaaatgtggt
cgcagatcac aacaactgag atggctcatg aacagaagga acaccctctc gctgagatgc
aaaagagctg tgtatgcgca ctgtatccta ccgatctggt tatacgggat ccagatttgg
ggaatcgcag ccaaatcaaa ttacaaacga atccaggtct gcccctggta cgtacggaac
tctacactcc ataaggacct caatctctac acagttgagg aacaaattgg gagacacaca
agtcgataca gtgacagact acagagtcat agtagccttc ttgcaagacg tcttatcccc
gctcgacctc t
>Derecta
---------- ---------- ---------- ---------- ---------- ----------
---------- ---------- ---------- ---------- ---------- ----------
---------- ---------- ---------- ---------- ---------- ----------
---------- ---------- ---------- ---------- ---------- ----------
---------- ---------- ---------- ---------- ---------- ----------
---------- ---------- ---------- ---------- ---------- ----------
---------- ---------- ---------- ---------- ---------- ----------
---------- ---------- ---------- ---------- ---------- ----------
---------- ---------- ---------- ---------- ---------- ----------
---------- ---------- ---------- ---------- ---------- ----------
---------- ---------- ---------- ---------- ---------- ----------
---------- ---------- ---------- ---------- ---------a atgcgtacat
catcggaaat tgctgaatgc atacgttgtc aggaattcgc ccacacatct aaatactgtc
gaagtcatcc aaattgtgct cgctgtggtg aaaatcactt aaccagttat gcgtgcgttc
caatggtcaa cagcctatct gtatacacta tggaggaaat cacacggcaa gttacaacgg
tttccagtgg taacaggagt atcttcgacg atcaatgggc actacaaaaa caggttagat
taacaaacca gcaataacct cagcaaaaac agcatcatgt a-aagcagca tcaatagcaa
gcactcccaa aggtttttcc tacgcagcta ttgcaagaaa tggcaataca acagacaagc
ctcgtctaca taacgtacat gccc------ ---------a attaaaggaa tataatatta
aacagcaacg cccgattaac gttcagtcaa tattggcaca gcagcaggaa caatttatga
attggc---- ---------- ----agcaac agcagcaatt cctatcgtga ct--------
----acagga gcaacaacaa caaaacaagc tgaatagcca acgactcgaa cggttggaaa
aactggtttt tgaaatggcc aatatgctga agcaatggac tggggatgca tcggctctcc
aactccattg taacgcttta ccatcgcaat gaaccctctg aagatactta tctggaatgt
aaatagtatt tcaggtaaag ccagagaagt tgagctcttc acacacaaca actgcgttga
cattattctc ctaaacgaga tcagactcaa cagagggaac accgttaaga tacatggata
tagcttttat cccgcataca aaccatcaag ccataatcat ggaatggggg gagcagcagt
actggtgagg agctctcttc gtcatttccc gcaaagagtt attgaaacga gaactattca
gatgtcttca gttaaggttt ccaccgggct gggagatatg gaatttagcg cgatatactg
tccaccaaca aatagaattg aagaaaggca cttcagtgac atacttgtct cttgtggcca
aaggtattta gttggtaagg actggaacat tagctatgat gtgacgggtg atacgtacaa
atcacccacg ggtccagaac tagcagaagc catttcagcc agaggtgctt atatccttgc
aacaagttca ccaacttgat acccacatgt gcccagtcat agacctacct gcattgattt
tgctgtgtac catgggataa acgtagacag aacaagtatt tgcgaaattt gggatctaaa
ctccgatcat gtagcccgtg ttgctactct acaaacagaa ggtgtctatg ttacgccatg
ttctcggtta aaaaa----- aaaccgattt ttttgttttc ag---acatc ttgaaaactc
ttttcaatta aatacggttc tcagctctaa ggaagagatc aagaacccag tgacatccga
acgcaaaata tacatagagc cgcttctgtt tccacgccgt ctgagcctga gacatgcccc
atgttatggc attgtactaa caagagaggc agagaactaa tcagaactaa aagacgtctt
cgaagaagag caattcgaac tcgggatcca tgggacagaa tcttgtggaa ccgagcagca
aagcaactca aaaccatctt aagggaactc agaagttatt tctttgagaa aaaagtatct
tccatggact acactgttga tgcaaactat tcgctgtgga agtgcacaaa agcgcttaaa
catcaaccac ttagatgggt acccg----- ---------- ---------- ----------
---------- -------tcg gctaccacct agaggatccc ttcactcctt atgactacgc
cacga----- cagacaagag agaatcacca gtacctacaa atgccattgc atttgtcttg
gcctattaag ccaataacga tagaagaaat atctgaaata atcaaattaa tgccgaaaca
caaagc---- ---------- ---------- ---------- ---------- ----------
---------- ---------- ---------- ---------- -tcctgggtc gtatcggcct
ataagcctct taccgtccct ttctaaatta tgggagagac tttttgccaa ttggatcaac
gacattataa gacaaggtaa tatattgccg gatcctcaat gtggatttcg aaagagacac
ggcacaatag aacaggtcca cagactggta aaacatatat tacaggcttt tgacgactgc
gagt------ ----cgtctt tatagacatg caacaagcct tcgacaatgt atggcatgtt
tgattattat gctagatgaa gaccctccta cctgcgccct acttctgtat tttgaagtca
tatct----- -----gaatt taaaatcacg gtgagaaata actactcttc tatatatcca
atgagaactg gagtcccaca gggcagtgtt ctcgaaccgt tactgtattc cttgtacact

gctgatatcg cttgcctgaa tttcgaacac atggtagcac ccaacaaggc tcttatggca
acctatgcag atgacattgc agttgtgtac aactctaggg acagtagaga gacagctaca
aggatacatt aa-------- ---------- ---------- ---------- ----------
---------- ---------- ---------- ---------- ---------- ----------
---------- ---------- ---------- ---------- ---------- ----------
---------- ---------- ---------- ---------- ---------- ----------
---------- ---------- ---------- ---------- ---------- ----------
---------- ---------- ---------- ---------- ---------- ----------
---------- ---------- ---------- ---------- ---------- ----------
---------- ---------- ---------- ---------- ---------- ----------
---------- ---------- ---------- ---------- ---------- ----------
---------- -
>Dananassae
---------- ---------- ---------- ---------- ---------- tatgaactct
aaaaaggtgc atagagaact gtcaccctca gtatcaatca gccaggaaag ctccagcaat
agatttgctt tactagacat ggaagtggac ctaaacagta ttgctgagga tgtagagaga
tctcctatgg ctgttcatga cgataatgta gacacagatg atttaggcaa tagatcccaa
cagaatgatt ctaatagaaa ttattatcca aaaccaccgc aaatagtttt aagcattgct
aacctaaatg atctatttga cctcatttcg gaggtcacta gcttggataa cgtaacagtt
aaagtcaatc aaggagttac ggctagaata tttcccaagg actctacaac ttatagagct
attgtcgacc attttgatag aatggaaatt gaattccata cataccaaat gaaggaagag
aagcctcaca gaatcgtagt taaaggactt catcatagca cactaactac tgaaattgtt
gccaatttta aaaatttggc ttcgatgctc ttcaggtgca taacccaaga tcaaggtcga
accatgatgt aaaattaaac atattttta ttaatattaa gccttgcgct agaattaatg
ctatatatga tatagtaaca ttgt------ ---------- ---------- ----------
---------- ---------- ---------- ---------- ---------- ----------
gtcgacaccc aaattgtgct cgatgtggcg aagaccattt tacttaccat gtgttcgacc
ccaagatcaa ccgccaatct gcatgcactg tggaggcaat cattcggcaa gctacaaggg
ttgtcagtcc tatcaaaatt acctcaggcg atctatgggt tctgcaaaga ----------
caacaacgcg acaacaacca caacaacgtc atcagcaaca accgacaact aatactccac
atatttctgc aagtttatct tatgcttcaa tagtaagaaa tggaaatgaa ccagctcaac
gccgtctaca tgatctccaa gt-----cac aagtacacaa atcgaaggaa aatgt---gc
aacaacaata tgcgactgat gttcaggcaa ttttagagca acagcaacag ctatttatga
aatggcaaca acaactccaa gaaaagcagc agcagcaatt ccttatgtgg ctaaggcagc
agcatcaaga acaacaaatg caaaacaagc tgaacagtca acgacttgaa cgtctttaaa
atatggtttt tgaaatggca aactcgatca agcaatggac tggagataaa tcacttcttc
agcttcccaa caacgcttca gcctcacaat gaacccactc aaaattctta tttggaacgt
caatggcatt ttaggtaaag ccagagaaat tgaattcttc gcgcacacca acgaagttga
tatcctactc ctaagtgaat taaggctcaa tcgaggagaa attgtcaaaa tttatggata
ttccttttat ccagcataca aaccggcaag acacaatcac ggcaccggag gagcggcggt
attggtaagg agctctctta gtcattttcc acaaagtgtt attgaaacac aaaccattca
aatgtcctct atcaaagttg acacaggttt gggtatcatg gtagtatgcg caatatactg
tcctccaaga aacagaattg aagaaaggca tttcactgac atactcgctt cttgtggtca
gaggtatctg gtcggtggtg actggaatgc tagacattgg atgtggggcg acacttacaa
ttcaccaaga ggacgcgagc tagctgaatc cactgtagta acaggggcta aaattcttgc
cactggttca ccaactagat acccttatgt acctggccac acagcttctt gcatagactt
tgcattatac catgggatac tggattctca aataagtata ggccaaaact gggatttgga
ctcggatcac atagctctcg ttgttaatct gcaaacaaat ggtgtacaga tcagaccaag
ccctcgttta attacaagcc gaactgatct caatactttc aaacaacatc ttgagacctc
ctttcaacta aattctgttc tgaactctag agaagacatc gagaatgcag tgacattctt
actgataata tatatagagc tgctactgtt tcaacgccac cagaacctgt tcttcgtcct
aagctatggc attgttctta caagggaggc agagagttaa tcaagagaaa aaggcgcctt
cgaagaagag caattcgttc tcaagatcct ttggaccgaa atagatggca ccaggctgag
aggcaactac gaactgtttt agatgaactc cgaagcgatt gtttcgagca aaagttatcc
tctatggaca ataccgttga cgcaaactac tcactatgga agtgcacaaa atcgcttaaa
cgacaacctt ttagacaagt tccagtccga tgtcctgatg gtgaactggc aaaaagaatt
ggagcaggct aatgcttttg gccagcatct agaggatcgc tttactccct ttaactttgc
atcggtagaa cagactagag agacctatca gagtttggag actccattgc agatgtcact
gcctattaag cctataagag tcgaggaaat atctgatgtc atacaatctt tgcccaaaaa
caaagcattt ttcatgcaat aattaggatc caagtgttcc caaaacagtg gaaattggct
gcgattatga tgatccataa gccagggaaa ccagaggttg atcctgagtc atatcgtcca
attagtctcc taccctgttt gtctaaactg tgggagagac ttattgcaaa ccgagttaaa
aggattatga ctgaaaacaa tatcttgcca gatcatcaat ttggctttcg aggaggacac
ggcaccgtgg aacaggttca cagactggtg caacatatct tgcaggcctt tgacgatcag

gagtattcca acgcagtttt tatcgatatg cagcaagctt ttgataaggt gtggcatgat
ggcttattgc tcaaaattaa aaatctttta cctgcgccgt actatggtct gtttagatca
tatttagaag tacgagaatt caaggtgaaa gtaaaagatt catactcgga caactttttg
atgagagcag gagttccaca gggaagtgtg cttggaccgt tgttgtactc actgtatacg
gcagatatac ctatcccgag cagccaacat atggtagccc cctctaaagc acttattgcg
acctatgcag atgacattgc agtcatttac aactacagat gtcacagaga agcttccaaa
ggattacagg agtacctatt cactcttgca gcttggtgca aaagatggaa cctgaagatc
aatccgtaga aaacaacgaa cgtctgcttt acactgaaga ggctcataat taatacccct
caaatccaac ttgagggagt taccctagaa cagcaaaacg aagcaaaata tcttggtatt
actctggaca agcgacttac tttcgggcca catctgaagt caacaactaa aaaatgtaat
atgagggttc agcagctgcg ttggatgata agcaaaagaa gtaccatgcc gcttaggtgc
aaaagggcag tatatgttca ttgcatcctg ccaatgtggc tttatggtgt acaaatttgg
gggatcgccg ccaaatcgaa ctataagcga atccaggtct gtccctggta cgtgcgtggc
tccactcttc atagagacct taacatacat actgtggaag aacagataaa tagacacaca
agccgttaca gcgacagact tctaagacat cgcagcctac tggctagaaa tttacttcca
gctagaccat t
>Dmojavensi
gcagcccaga aaatgcttaa aactaaaagg aagatacctc tatctcccaa taacaataac
aaaagagcaa acagagttga gtcacactcc gcttcaacta atatggcaaa atccagtaac
agctttgctt tactggacat ggatatggac gcaaccagtg atgatgtgaa c------act
gcttctattg ttgatcatag caacactgct gatgcaattg atacagtcaa cagccatcag
aataaagaac ccagcaacaa ccaacagact aagccaccgc aaatcgttgt gagtatcact
gatcttaatg atctatttga aatcattagt gaagtcacca gtattgataa cgtatcagtc
aagatcaacc aaggagtgac ggctagaata tttcccaaag atagcgacac ttacagagct
atcgtaagcc attttgatgc gataggtatt gagttccata cgtatcagat gaaggaagaa
aaaccttaca gaattgtagt aagagggcta caccatagta ccttgaacaa tgaaataatt
gccaatttta aaaatatggc ttcgatgctc tgcaaattca caacccaagg tcacggtcaa
acagggatgc aaaattaaat atattttttg taaacataaa accttgtaaa aacattaacg
aagtttacaa tataaaaaca ctttgccgcc aaatggtgag agttgaaagg atgcgtaaaa
cttctgaaat cgtcatatgt acccgatgcc aagaatatgg ccatacagcc aaatactgcc
gtcgccatcc taactgtgca cgctgtggcg aggaccatcc cacaatcttt gcgcacgttc
acaagatgca ccgcctacct gccttcactg tggaggtaac cacatggcaa gctacaaagg
ctgcccgtgg taccaggagt tctatcgacg ttcactgggc tcctctacga caagaagaat
caacaagacc acaacagcca caacggaagc accagcatca accaaccaca aagctatcaa
atacttctgg aggaatatcc tatgccgcgg tagcaagaaa tggaagcgca tctgctcaaa
gccgcataca taatattcaa gcgcaagccc agttagctaa acctatagga catatcgcac
agcagcagca tcaagttgat gtccaatctt tactggaaca acaacaacaa caatttttga
aatggcaaaa agacttgcaa gtgcagcaac agcagcaatt tctttcatgg cttcaagcac
aacaacgcga acagcaacaa caaaacaaga gaaacagtga acgactagaa cgtctagaaa
aaatggtcca cgaaatggcc agcatgctca agcaatggac tggggattca tcgactcacc
agcttcttaa caacgcctca gcctcacaat gaacccactc aagattctca tctggaatgt
caatggcatt gcaggaaaag ccagagatgt ggagctattc gcgcacaaca acaacatcga
cattctacta ttaaatgaaa tcagactgaa tcgaggagat acagtcaaaa tatatggcta
caccttctat ccagcttaca agccatcaag ccacaaccat ggtatgggag gagcggctat
attcgttaga agttcactgc gccacttccc acaaagggtt attgaaactc aaaatataca
aatgtctgca atcaaagttg ccactggatt gggagacatc gaattttgcg ccatatactg
cccaccaagg aacagaatag aagaaagaca attcagcgac atactcgctt cttgcggcca
aaggtatctt attggtggtg actggaatgc acgacactgg ctgtggggag attcatacaa
ttcacccagg ggaagagaac tagcagaagc catcacggcc agaggagcta atattcttgc
aacaggttct cctactaggt acccatatat atccagtcat actccctctt gcattgattt
tgcattatac catgggatac agcattatca agcaaatatt taccaaagct gggacttaga
ctctgaccat ttagccctta ttgccgagtt acacatcgat ggtcattata tcaggccaag
cccaaggcta ataaccagcc gtactgacat cgaggctttc agacaacagc tagatgactc
cattcatttg aatttgtgtc tgaactccgg cgaagacatc gagaatgcgg tgataatctc
tcggagaaca tatatagagc tgctgctgct acaacgccgc ttaatcctgc gcctcgtccc
aaattatggc attgttctaa cgagagaagc aaagaactca tcagaacaaa aaggcgcctt
cgtagaagag caattcgatc ccaagatcct tgggaccgcc ttttgtggaa ccgggtagca
agacagctac gtaacctttt aagggaactc agaagcgatt tcttcgagca aaaactggct
tccatggact acactattga tgcaaactat tcgttatgga aatgcacaaa atcactcaaa
cgacaacctt ttagacaggt acctgtccga tgccctaacg gcgaagttgc taaatgaatt
ggaacaggct aacgcattcg gatgtcatct agaagaacgt tttactccat ttagttatgc
tacgacggag cagaccatgg agatacagct atatttgcag accccccttgc agatgtctct
gcctattgag cctataagga ttgaagaaat taccgaagcg atccaaatat tgccgaaaaa
taaagcatct tcaatgccat catcaggctc caagtgttcc caaggcaatg gaaattagct

gctatcttga tgatccacaa gcctggcaag ccggaagagg atccagagtc atatcggcct
ataagtctac tgccctcctt gtctaaacta tgggagagga ccattgccaa ccgcattaac
gcaatattta gacaatccaa tatcctaccg gatcaccaat ttggattccg ggagggacac
agcacagtgg aacaggtcca tagactggtg aaacacatct tgcaggcttt tgatgactct
gaatactcca atgctgtctt tatcgacatg cagcaagcgt ttgataaggt gtggcacgat
ggattactgt acaaaataaa aaaccttcta cctgctccgt actatggcct cttaagatct
tatctagaag accgtgtgtt ccaagtcaag gtaaaagata cactctcgtc cacataccct
atgagagcag gagtgccgca gggcagtgtt cttggtccgc tgctgttctc cttgtacacc
tctgatatac ctagcccgtc ctctcaacat atggatgctc cctcgaaagc tgtcattgcc
acatatgctg atgatattgc catcatctat aactcaaaaa acgtggttga agcaagtaca
ggactacaga gatatctgga tactctcgct gattggtgca agcggtggaa tctcaaagtt
aacccgctaa aaacattgaa cccttgcttc actctaaaaa ggctggcaat gcataccccc
ccaatccaga tgtcgggagt aaccctacag caacctgctc aggtgaaata tcttggcatc
acactggaca agcgcctcac cttcggtcca cacctcaaag ctacggtgaa aaaatgtcgt
cacagactgc aacaattaag atggctcacc aatagaaaga gcaccttacc gctgagatgc
aaaagagctg tctatgtgca ctgcatccta ccaatatggc tctatggagt gcaaatctgg
gggatcgctg caaaatcgaa ttataaaaga gtgcaggtct gtccctggta catacgcggc
tcaacactgc atagagacct caagctgtct actgtcgaag aacaaataaa taaacacaca
agcagatact cagacagact tgtacgacat caaagcctac tcgcaagagg tctaacacct
gccagacccc t
>Dvirilis
---------- ------tgaa aaataagagg aagaaaccat tatctcccaa tactagctct
aagaggccga ataaakattt gtcgccctca gcatcaacaa gtatggcaat ctctggcaac
aggtttgcct tgctagacat ggamatggac atggatgtaa cccatgttga tccaaatagc
gcttctgagg ctgatcataa cgtcagtatt gatgtaagtg acacagccaa tmacccacag
cataacgcaa tcaataccaa cgaccagcta aagccaccgc aaatagttgt gtgtgttact
gaccttaatg atctttttga actcattatg gaggtcacta gtctagataa cgtggcagtg
aaagtcaacc aaggagaaac ggccagaata tttcccaaag acagtgacac ttacagagca
attgtcaaac attttgatgg tattggtatt gagttccata cctatcaatc gaaggaagag
aaaccataca ggatagtagt taaggggctt caccatagca ctcttaacaa cgaaattatt
gacaawttaa aaaaaatggc tttgatgctc tgcaggtaca taacccaaga ccaaggagta
acaaagatgg aaaactcaat atatttttca ttaatataag gccatgcgca aaaattaatg
atatatgtga tatcaaaac- -tctgccgac aaaaagtgag gatagaaagg atgcgcaaaa
catctgaaat cgcccaatgc acacgatgtc aggaatacgg acacacagcc aaatactgcc
gtcgtcaccc caattgtgca cgatgcggtg aaaatcatct cacaagctat gcacacgtcc
ccatgatgaa cagccttcct gtttgcactg tggagggaat catatggcaa gctacaaagg
ttgccaatgg taccaggact atctacgacg atcaatgggc tcttcaatga caggaggaat
caacaaacca gaaacaacca caaaggaaac agcagcagca gccgacagca aaaataccta
acattcctag aggcaagtcc tatgcctcaa tagcaaggaa cggcaacgaa cctgcccagc
gccgtctaca tagtttgcaa gcgcaacaac agctacttaa gcgaatagaa aatatcaggc
agcagcatca tccagctgac gttcaatcat tattggaaca acagcaacag caattttt---
-aatgcaaca acagctgcaa ctacagcagc agcagcaatt cctcttgtgg cttcaggagc
agcaacggga acaacaacaa caaaacaagc aaaatagtga acggctagaa cggctggaaa
aaatggtcca tgaaatggcc agcatgataa agcaatggac akgggatgga tcgactcccc
agcttcatag caacgcctca gcctcccaat gaatccactc aaggtcctta tctggaatgt
taatggcatc gcaggaaaag ccagtgaagt tgagctcttc gcgcacaaca acaatgtcg-
---------- --aagtgaaa tcagactaaa ccgaggagaa acagtcaaaa tacacgggta
caccttctat ccagcttaca agccatcacg tcacagtcac ggtgtcggag gagcggcggt
attcgtgagg agtactctcc gccattttcc acaaagagta attgaaacga gcaccataca
aatgtcttca ataaaaattg ccacaggttt gggagagatg gaatatagcg ccatatactg
cccaccaaga aatagaattg aagaaaggca tttcagtgac atactcgctt cttgcggcca
aaggtatctg gttggtggag actggaatgc gcgacattgg ctgtggggag atacctacaa
ttcacccagg ggccgagaac tagccgaagc cattacagcc agaggagcta atattcccgc
aacaggatca ccaactagat atccatatgt gtctagtcac acacctagtt gcattgattt
cgcattatac catgggatac cggattctca aacaaatatc ggccaaagct gggatctaga
ctctgaccat atagccct-g ttgtaaattt gcaaacagat ggttcttatg tcaggccaaa
ccctcgatta attaccaacc gtactgacat catggctttc agacaacatc ttgaatgctc
gattcaatta aatccgacct tggactctgg agtagacatt gagaatgcag tgattttcta
actgaaaata tacatagcgc tgctactgcc acaactccac acatcaccga gtcacgtccc
acgttatggc attgttttca cgagggaggc aaagagctga tcagaacaaa gaggtgcctt
cgaagaagag caattcgatc acaagatcct tgggaccgaa ttttgtggag ccgagcagca
aaacagctac gtaacctcct aagggaactc agaagtgaat ttctt---cg aaaactggct
tccatggact acaccgttga tgcgaactac tctctacgga aatgtacaaa atcactcaaa
cgacaaccct ttagacaggt acctgtccga tgtcctggtg gcgaacttgc gaaca-----

---------- ---------- ---------- ---------- ---------- ----------
---------- ---------- ---------- ---------- ---------- ----------
---------- ---------- ---------- ---------- ---------- ----------
---------- ---------- ---------- ---------- ---------- ----------
---attatga tgattcacaa gccaggcaaa ccagaagctg atcctgagtc gtatcggcca
ataagtctct taccctccct gtct-aacta tgggaaagga ctattgccaa tcggattact
gccattacaa gccaatgcaa tatcttgccg gatcaccaat tcggatttcg agagggacac
ggcactgtgg a--caggtca cagattggtg aagcacatca tgcaggcttt tgacgaccta
gagttctcaa acgctgtctt cattgacatg cagcaagcgt tcgataaggt gtggcacgat
ggattactgt gcaaaataaa aaacctttta cctgcaccgt actatggcct tctaaggtca
tatctggaag tacgagagtt caaggtcaag gtaagggaca catactcgtc cacctatact
atgagagcag gagtgccaca gggcagtgtt cttggtccgc tgctctactc gttgtatacc
tcagatatac ccagccctac ctctcaacat atggacgatc cttctaaggc tattattgcg
acatatgcag atgacatcgc agtcatttat aactccaaaa gttgcgtaga agctagcaat
ggacttcagg ggtatctgga tactctcgca gcttggtgca aacggtggaa tttgaaggtt
aacccgttaa aaactattaa cccatgcttc accctcaaaa tgcag---at gcatatccct
ccaattcagc ttgaaggagt taccctggag cagccgccac aagctaagta tctcggcatc
accttagaca agcgccttac ctttgggcca cacctcaaag ctacggtaaa aaaatgtcgc
cacagactgc aacaactgcg gtggctcaac aataaaagga gcaccttgcc gctgagatgc
aaaagagctg tatacgtgca ctgtattttg ccgatatggc tctatggagt gcagatttgg
gggattgcag ccaaatcaaa ttataaacgc atacaggtct gcccctggta cgtacgcggt
tctacactcc ataaggacct caaagtgcat acagtcgaag aacagattgg aaggcacaca
agcagataca gcgacaaatt gctgagacac cgcagcctgc tcgcaagagg actactccct
gcccaacctc t
>Dsimulans
gatgcgcaaa aaacgaaaga gaggaagagg aagaccaatt taacacccaa ccatagctct
aaacgagctg tcagagacgc gccatatcca gccataaata gtaattcaaa ctcaaacaat
aggtttgcca tgctagacat ggaattggac gaaaccagtg atggcataga ttgtcatacc
tcgtgtgcat cggctgctaa tgatgactgt gtaccaaatg tgacatccaa tagcccacaa
cagtatac-- ----tgataa gcagaattca aaaccaccgc aaatagtact gagccttacc
aatcttaatg atctctatga gctcattacg gaggtcacta gcctagataa tttaacagtt
aaagtcaatc aaggggaaac agtgagaata ttacccaaag actctgatac ttacagagct
attgttaatc tttttgataa ttcgggaatt gaattccata cgtaccaaat gaaggaagag
aagcctcaca gaatagttgt taagggactc caccatagca ccctaaccta cgaaattatc
gacaacttta aaaatatggc tttgatgttc tacaagtaca caacccaaga tccaggagaa
atagagaaga aaaacttaat atattcttca ttaatataaa gacctgtgca aaaattaatg
acatatacga tattaacaca atatgccgac agaaagtgcg gatagaaaga atgcgtaaat
catctgaaat tgcacaatgc atacgttgtc aggaattcgg ccacacagct aaatactgtc
gtcgtcatcc caactgtgct cgatgtggtg aaaatcactt aacaagctat gcgtacttcc
caatgatcaa cagcctatct gtatacactg tggaggaaat cacacggcaa gttacaaggg
ttgccagttt taccaggagt atcttcgacg atcaatgggc actgtaaaga caagtttgat
aaacaaacca acaacaaccc cagcaaaaac ---------- ---agcagca tcaatagcta
gcactcccaa aggcttgtcc tacgcagata ttgcaagaaa tggcaataca acagcccagc
ctcgtctaca taatgtac-- ---------- ---------a attaaaggga actaatatta
agcagcaaca cccgcttgac gttcaatcaa tattggcaca gcaacaggaa caatttatga
agtggcagca acagcttcaa cagcaacaac agcagcaatt cctatcgtgg ctacagcagc
agcaacagga gcaacaacaa caaaacaagt tgaatagtca acgactcgaa aggctggaaa
atattgtttt tgaaatggcc aatatgctga agcaacggac tggggataca tcggctcccc
aactccatag taacgcttta ccatcgcaat gaaccctctg aagattctta tctggaatgt
aaatggtatt tcaggtaaag ccagagaagt agagctcttc gcacacaaca acggcattga
cattcttctc ctaaacgaga tcagactcaa cagagggaac acagttaaga tatatggata
cagcttttat cccgcataca aaccttcaag ccataatcac ggaatgggag gagcagcagt
actggtgaga agttctcttc gtcatttccc gcaaagagtt attgaaacga gaactattca
gatgtcttca gtcaaggtct ccaccgggct gggagatatg gaatttagcg cgatttactg
tccaccaaga aatagaattg aggaaaggca cttcagtgac atacttgtct cttgtggaca
aaggtatttc gttggtgggg actggaacgc ccgacattgg ctatggggtg acacgtacaa
ttcacccaga ggtcgagaac tagcagaagc catttcagcc agaggggctt atatccttgc
aacaggttca ccaactagat acccacatgt gcccagtcac agacctacct gcattgattt
tgctgtgtac catgggataa acttagacag aactagtatt tctgaaaatt gggatctaga
ctccgatcat gtagcccttg tggctactct acaaacagaa ggtgcctatg ttagaccatg
ctctcggtta ataaacagca gaactgatct ccttgttttc agacaacatc tggaaaactc
tctccaatta aatacggttc tgagctctaa ggaagacatc gagaacgcag tgacagtcta
acgcaaaata tacatagagc cgcttctgct tctacgccgt ctgagcccga gatacgcccc
aagttatggt attgttctaa caagagaggc agagaactta tcagaactaa gagacgcctt

```
cgaagaagag caattcgaac tcaggatcca tgggacagaa tcttgtggaa ccgagcagca
aagcaactca aagtcgtctt aagggaactc agaagtgatt tctttgagca aaaattatcc
tccatggact acaccgttga tgcaaactat tcgctgtgga agtgcacaaa agcccttaaa
cgacaaccac ttcgatgggt acccgtacgc tgtccaggtg gggaatttgc aaaaagaagt
ggaacaggct aatgcattcg gcttccacct agaggatcgc ttcactcctt acgacttcgc
cacgacagaa caaataagag agactcacca gtacctacaa atgccattgc agatgtcttg
gcctattaag ccaataagga tagaagaaat ccttgaaata attaaattac tgccgaagca
taaagcatct ttaatgctat tttaaggatc caagtgttcc caagacagtg gaaaatggct
gctattttga tgatccacaa gcctggaaag ccggaagatg atcctgagtc gtatcggcct
ataagcctct taccctcact ttctaaatta tgggagagac ttattgccaa tcggataaac
gacattataa gacaaggcaa tatcttgccg gatcatcaat ttggatttcg aaagggacac
ggaactattg aacaggtcca cagactggtg aaacacatac tacaggcttt tgacgactgc
gagtactcca acgccgtctt tatagatatg caacaagcct tcgacaaagt atggcatgtt
ggattattat gcaagataaa gacccttcta cctgcgccct acttctgtat tttaaagtca
tatctagaag aaagacaatt taaaatcacg gtgagaaata gctactcctc tatataccca
atgagagctg gagtccctca gggcagtgtt ctcggaccgc tactgtattc cttgtacact
gctgatatcc cttgcccgag tttcgaacac atggcagcac cgaacaggac tcttattgca
acctatgcag atgacatcgc agttgtatat aactctaggg acagcagaga ggcagctaac
ggactacaag aatatattaa tgctctggca gcctggtgta aacggtggaa cctaaaaata
aacccactga aaacaacaaa cccgtgcttc acgttaaaaa cgcttatccc aaacacccct
ccaattcggc tagaaggagt taccctgaat cagcccctgc aagcaacata tctaggtatc
accctggata aacggctcac ctttgggccg catctaaaaa acacagtaaa aaaatgtggc
cacagatcac aacagctgag atggctcatg aatagaagga gcactctttc gatgaggtgc
aaaagagctg tgtatgcgca ctgtatcgta ccgatatggt tatacgggat ccagatttgg
ggaattgcag ccaaatcgaa ttataatcgt atccaggtct gcccctggta tgtacgtaac
tctacactcc ataaagacct caatattcac acagttgagt cacaaattgg gagacataca
agtcgatata gtgacagatt actgagccat agcagtcttc ttgcaagacg tctcatcccc
gctcgacctc t
>Dmelano
---------- ---------- ---------- ---------- ---------- ----------
---------- ---------- ---------- ---------- ---------- ----------
---------- ---------- ---------- ---------- ---------- ----------
---------- ---------- ---------- ---------- ---------- ----------
---------- ---------- ---------- ---------- ---------- ----------
---------- ---------- ---------- ---------- ---------- ----------
---------- ---------- ---------- ---------- ---------- ----------
---------- ---------- ---------- ---------- ---------- ----------
---------- ---------- ---------- ---------- ---------- ----------
---------- ---------- ---------- ---------- ---------- ----------
---------- ---------- ---------- ---------- ---------- ----------
---------- ---------- ---------- ---------- ---------- ----------
---------- ---------- ---------- ---------- ---------- ----------
---------- ---------- ---------- ---------- ---------- ----------
---------- ---------- ---------- ---------- ---------- ----------
---------- ---------- ---------- ---------- ---------- ----------
---------- ---------- ---------- ---------- ---------- ----------
---------- ---------- ---------- ---------- ---------- ----------
---------- ---------- ---------- ---------- ---------- ----------
---------- ---------- ---------- ---------- ---------- ----------
---------- ---------- ---------- ---------- ---------- ----------
---------- ---------- ---------- ---------- ---------- ----------
---------- ---------- ---------- ---------- ---------- ----------
---------- ---------- ---------- ---------- ---------- ----------
---------- ---------- ---------- ---------- ---------- ----------
---------- ---------- ---------- ---------- ---------- ----------
---------- ---------- ---------- ---------- ---------- ----------
---------- ---------- ---------- ---------- ---------- ----------
---------- ---------- ---------- ---------- ---------- ----------
---------- ---------- ---------- ---------- ---------- ----------
---------- ---------- ---------- ---------- ---------- ----------
---------- ---------- ---------- ---------- ---------- ----------
---------- ---------- ---------- ---------- ---------- ----------
---------- ---------- ---------- ---------- ---------- ----------
---------- ---------- ---------- ---------- ---------- ----------
```

```
---------- ---------- ---------- ---------- ---------- ----------
---------- ---------- ---------- ---------- ---------- ----------
---------- ---------- ---------- ---------- ---------- ----------
---------- ---------- ---------- ---------- ---------- ----------
---------- ---------- ---------- ---------- ---------- ----------
---------- ---------- ---------- ---------- ---------- ----------
---------- ---------- ---------- ---------- ---------- ----------
---------- ---------- ---------- ---------- ---------- ----------
---------- ---------- ---------- ---------- ---------- ----------
---------- ---------- ---------- ---------- ---------- ----------
---------- ---------- ---------- ---------- ---------- ----------
------atct ttaatgctat tttaaggatt caagtgttcc caagacagtg gaaaatggct
gttattttga tgatccacaa gcctggaaaa ccagaagttg atcctgagtc gtatcggcct
ataagcctct taccctccct ttctaaacta tgggagagac ttatttccaa ttggattaac
gacattataa cagaaggcaa tatcttgccg gatcatcaat ttggattttg aaagggacac
ggaactattg aacaggtc-- cagactggtg aaacacatat tacaggcttt tgacgactac
gagtactcaa acgctgtctt tatagatatg caacaagcct tcgacaaagt atggcatgtt
ggattattgt gcaagataaa gacccttcta cctgcgctcg act----cat tttaaagtca
tatctggaag gacgacaatt taaaatctta gtgagaacta actactcctc tatataccca
atgagagctg gagtcccaca gggcagtgtt ctcggaccgc tactatattc cttgtacact
gctgatatcc cttgcccgag tttcgaacac agggaagcac cgaacagggc tcttattgca
acctatgcag atgacatcgc agttgtatat aactctaggg acagcagaga ggcagctaac
ggactgcaat aatatattaa tgctctggca gcctggtgta aacggtggaa cctaaaaata
aacccactga aacaacaaa tccatgcttc acattaaaaa cgcttgtcca aaacacccct
ccaatccggc tagaaggagt taccctgaat caaccgctgc aagcaacata tctaggtatc
accgtcgata aaaggctcac cttttggttg catctcaaaa acacagtaaa gaaatgtggt
cacagatcac aacagctgag atggctgatg aatagaagga tcactctttc gctgaggtgc
aaaagagctg tgtatgcgca ctgtatcgta ccgatgtagt tatacgggat ccagatttgg
ggaattgcag ccgaatctaa ttataaacgt atccaggtct gttcctggta tgtacgtaac
tccacactct ataaagacct caatattcac acagttgaga cacaaattgg gagacataca
agtcgataca gtggcagatt actgagccat agcagtcttc ttgcaagacg tcttatcccc
gctcgacctc t
>Dbuzzatii
gcagcccaga aaatgcttaa aaataaaagg aagaaaccta tatctcccta taaaaataat
aaaagagcaa gcagagaagt gtcccactcc gcttcaacta atatggctaa atcaagtaat
gcctttgctt tactggatat ggacatggac gtaaccagtg atgtcgagaa tattgataat
catgtgattg ctgatcatag taatgctgct gatgcaatcg acacaggcaa caaccaccta
aatcaagaac ccgacaacaa caaaaagccg aagccaccgc aaatcgttgt gagcatcact
gatcttaatg acctatttga aatcatcagt gaggtcacta gcgttgacaa cgtttcagtc
aaagtcaacc aaggagtaac ggctagaata tttgccaaag atagcgtcac ctacagagct
atagtaagcc attttgatac aataggtatt gagtttcaca catacaaat gaaggaagaa
aaaccttaca gaatcgtagt aaaagggctt caccatagta ctctgaatca cgaaataatt
gccaatttta aaaatatggc tttgatgctc tgcaagtgca caacccaaga tcaaggtcaa
acagagagga aaaattaaat atattctttg taaatataaa accttgcaaa aaaattaacg
atatttacag tataaaaact ctttgtcggc aaatggtgag agtagaaagg atgcgtaaag
cttccgaaat tgttatatgc acacgatgcc aggaatatgg tcattcagcc aaatactgcc
gtcgccatcc aaactgtgca cggtgtggcg aggaccatcc cacattctgt gcacacgatc
acaagatgaa ccgcctacct gcattcactg tggaggtaac cacatggcaa gctataaagg
ctgtcagtgg taccaagact tttatcgacg ttcaactggt ccttctacga caagaagaac
caacaaggcc gcaacagcta caaggtaagc aacagcatca accaaccaca aagttaccaa
atacatctgg aggtcgatcc tatgccgcaa tagcaagaaa cggatacgta tctgctcaga
gccgcataca caatatccaa gcgcaagccc agctagctac acctatagga aatattccac
agcagcagca acaaattgat gtccaatcgc tattggaaca acaacaacat caatttctga
aatggcaaaa agagctgcaa gttcagcagc agcaacaatt tctttcatgg cttcaggcgc
aacaacgcga acaacaacaa caaaataaga gaaatagtga tcgtctagaa cggctcgaaa
aaatggtcca cgaaatggcc agcatgctca agcaatggac tgggggccca atgactcgcc
agcttcctaa caacgcctca gcctcacaat gagctcacta aagattctca tttggaatgt
caatggcatt gcaggaaaag ccagagatgt agagctattt gcgcacaaca acaacgtcga
cattctactt ttaaatgaaa ttagactgaa tcgtggagat acagccaaaa tctatggcta
taccttctat ccggcttaca aaccatcaag tcataaccat ggtatgggag gagcggctat
attcgtgaga aattctctgc gccacttccc acaaagagtt atcgaaactc aacatataca
aatgtctgca attaaagttg ccacaggatt gggagacgtt gaattctgcg ccatatactg
cccaccaagg aacagaattg atgaaaggca attcagcgac atactcgctt cctgtggcca
aaggtatttt attggtggcg actggaatgc gcgacattgg ctgtggggag actcatacaa
```

```
ctctcccagg ggcagagaac ttgcagaagc cattacagcc agaagcgcta atattcttgc
tacaggatct cctactagat acccgtatat atccaatcac actccctctt gcattgattt
tgcattatac catgggatac agcattacca agtaaatata caccaaaact gggacttgga
ttctgaccac ctagccctca ttgccgagtt gcacattgat ggttctaata tcagcccaag
cccaaggcta ataaccaacc gtactgacat tgcggctttc agacaacagc tagatgactc
catccaatta aatccgtgtc tgaactcagg tgaagatatc gagaatgcgg tgatcttctc
gcggagaaca tatatagagc tgctgctgct acaacgccga taaacaccgc gtttcgtccc
aaattatggc attgttctaa caagagaagc aaagaactta tcagaacaaa aaggttcctt
cgtagaagag caattcgatc ccaagatcct tgggaccgaa ttctgtggaa ccgtgcagca
agacagctac gcaatctctt aagagaaatc agaggtgact ttttcgagca aaaactggct
tccatggact acactacaga tgcaaactac tcactatgga aatgcacaaa atcgcttaaa
agacaaccat ttagacaggt acctgttcga tgccctagtg gcgaacttgc taaaagaatt
ggaacaggct aacgcattcg gaagccatct tgaggatcgt tttactccac ataattacgc
tacagcggag cagaccatgg aaacatatag aagtttgcag accccattgc agatgtccct
gcctattcag cctatacgga ttgatgaaat aactgaagcg atccaaatat tgccgaaaaa
taaagcatct tcaatgccat catcaggctc caaaagttcc caaggcaatg gaaacttgcg
gctatcttga tgattcataa accgggcaaa ccggaacagg atccagactc atatcggcct
ataagcctac tgccctccct gtctaaacta tgggagagaa ccatcgctaa ccgaatcaat
gcaataatag cgcaatccaa tatcctaccg gatcaccaat tcggttttcg ggcgggatac
agcacagtgg aacaagtgca cagattggta aaacacatcc tgcaggcctt tgatgactta
gaatactcca acgctgtctt cattgacttg cagcaagcgt ttgacaaggt gtggcacgat
ggattactgt gcaaaataaa aaaccttctg cctgctccgt actatagcct cttaaaatcc
tatctagaag accgtgagtt taaagtcaag gtaaaggata cactctcgtc cacatatcct
atgagagcag gagttccgca gggcagtgtt cttggtccgc tgctgttctc cttgtataca
tcggacattc ctagcccgtc ctcccaacat atgaatgctc cctctaaagc cgtcattgcc
acatatgctg atgacatagc aatcatatac agctcaaaaa accttgcaga atcaagtatt
ggactacaga gataccttga tactttcgct ggttggtgca agcggtggaa cctaaaagtt
aacccgctaa aaacactaaa cccctgcttc accctaaaaa ggtcggcaat acataccccc
ccaatccaga tgtccggagt aaatcttcag caacttgcac aggtgaaata tcttggcatc
acactggaca agcgtctcac ctttgggcca cacctcaaag ccacggtaaa aaaatgtcgt
cacagactgc aacaactaag atggctcaac aataaaaaga gcaccttacc gctgag----
---------- ---------- ---------- ---------- ---------- ----------
---------- ---------- ---------- ---------- ---------- ----------
---------- ---------- ---------- ---------- ---------- ----------
---------- ---------- ---------- ---------- ---------- ----------
---------- -
>Dkoep28
gcagcccaga aaatgcttaa aaataaaagg aagaaacctc tatctcccta taaaaataat
aaaagagcaa gcagagaagt gtcccactcc gcttcaacta atatggctaa atcaagtaat
gcctttgcat tactggatat ggacatggac gtaaccagtg atgtcgagaa tattgataat
catgtgattg ctgatcatag taatgctgct gatgcaattg acacaggcaa caaccaccta
aatcaagaac ccgacaacaa caaaaagcca aagccaccgc aaatcgttgt gagcatcact
gatcttaatg acctatttga aatcatcagt gaggtcacta gcgttgacaa cgtttcagtc
gaagtcaacc aaggagtaac ggctagaata tttgccaaag atagcgacac ctacagagct
atagtaagcc attttgatac aataggtatt gagtttcaca cataccaaat gaaggaagaa
aaaccttaca gaatcgtagt aaaagggctt caccatagta ctctgaatca agaaataatt
gccaatttta aaaatatggc tttgatgctc tgcaagtgca caacccaaga tcaaggtcaa
acagagagga aaaattaaat atattcttcg taaatataaa accttgcaaa aaaattaacg
atatttacag tataaaaact ctttgtcggc aaatggtgag agtagaaagg atgcgtaaag
cttccgaaat tgttatatgc acacgatgcc aggaatatgg tcattcagcc aaatactgcc
gtcgccatcc aaactgtgca cggtgtggcg aggaccatcc cacattctgt gcacacgatc
acaagatgaa ccgcctacct gcattcactg tggaggtaac cacatgacaa gctataaagg
ctgtcagtgg taccaagact tttatcgacg atcaactggt ccttctacga caagaagaac
caacaaggcc gcaacagcta caacgtaagc aacagcatca accaaccaca aagttaccaa
atacatctgg aggtcgatcc tatgccgcaa tagcaagaaa cggatacgta tctgctcaga
gtcgcataca caatatccaa gcgcaagccc agctagctac acctatagga aatattccac
agcagcagca acaagttgat gtccaatctc tattggaaca acaacaacaa caatttctga
aatggcaaaa agagctgcaa gttcagcagc agcagcaatt tctttcatgg cttcaggcgc
aacaacgcga acaacaacaa caaaataaga gaaatagtga tcgactagaa cggctcgaaa
aaatggtcca cgaaatggcc agcatgctca aacaatggac tgggggccca acgactcgcc
agcttcttaa caacgcctca gcctcacaat gagctcacta aagatcctca tttggaatgc
caatggcatt gcaggaaaag ccagagatgt agagctattc gcgcacaaca acaacgtcga
cattctactt ttaaatgaaa ttagactgaa tcgaggagat acagtcaaaa tctatggcta
taccttctat ccggcttaca aaccatcaag tcataaccat ggtatgggag gggcggctat
```

```
attcgtgaga aattctctgc gccacttccc acaaagagtt atcgaaactc aacacataca
aatgtctgca attaaagctg ccacaggatt gggagacgtt gaattctgcg ccatatactg
cccaccaagg aacagaattg aagaaaggca attcagcgac atactcgctt cttgtggcca
aaggtatttt attggtggcg actggaatgc gcgacattgg ctgtggggag actcatacaa
ctctcccagg ggcagagaac ttgcagaagc cattacagcc agaggcgcta atattcttgc
tacaggatct cctactagat acccgtatat atccagtcac actccctctt gcattgattt
tgcattatac catgggatac agcattacca agtaaatata caccaaaact gggacttgga
ttctgatcac ctagccctca ttgctgagtt gcacattgat ggttctaata tcagcccaag
cccaaggcta ataactaacc gtactgacat tgcggctttc agacaacagc tagatgactc
tatccaatta aatccgtgtc tgaactcagg tgaagatatt gagaatgcgg tgatcttctc
tcggagaaca tatatagagc tgctgctgct acaacgccga ttaacaccgc gtttcgtccc
aaattatggc attattctaa caagagaagc aaagaactta tcagaacaaa aaggttcctt
cgtagaagag caattcgatc ccaagatcct tgggaccgaa ttctgtggaa ccgtgcagca
agacagctac gcaatctctt aagagaaatc agaggtgact ttttcgagca aaaactggct
tccatggact acactacaga tgcaaactac tcactatgga aatgcacaaa atcgcttaaa
cgacaaccat ttagacaggt gcctgttcga tgccctaatg gcgaacttgc taaaagaatt
ggaacaggct aacgcattcg gaagccatct agaggatcgt tttactccat ataattacgc
tacagcggag cagaccatgg agacatatag aagcttgcag accccattgc agatgtccct
gcctattcag cctatacgga ttgatgaaat aactgaagcg atccaaatat tgccaa----
---------- ---------- ---------- ---------- ---------- ----------
---------- ---------- ---------- ---------- ---------- ----------
---------- ---------- ---------- ---------- ---------- ----------
---------- ---------- ---------- ---------- ---------- ----------
---------- ---------- ---------- ---------- ---------- ----------
---------- ---------- ---------- ---------- ---------- ----------
---------- ---------- ---------- ---------- ---------- ----------
---------- ---------- ---------- ---------- ---------- ----------
---------- ---------- ---------- ---------- ---------- ----------
---------- ---------- ---------- ---------- ---------- ----------
---------- ---------- ---------- ---------- ---------- ----------
---------- ---------- ---------- ---------- ---------- ----------
---------- ---------- ---------- ---------- ---------- ----------
---------- ---------- ---------- ---------- ---------- ----------
---------- ---------- ---------- ----tcgaa- ---------- ----------
---------- ---------- ---------- ---------- ---------- ----------
---------- ---------- ---------- ---------- ---------- ----------
---------- ---------- ---------- ---------- ---------- ----------
---------- ---------- ---------- ---------- ---------- ----------
---------- ---------- ---------- ---------- ---------- ----------
---------- -
>Dkoep351
gcagcccaga aaatgcttaa aactaaaagg aagaaacctc tatctcccta taacaataat
aaaagtgcaa gcagagatga gtcacactac gctccaacta atataacaaa atccagtaat
agctttgctt tactagacac agacatggac gtaaccaacg atgatgtgaa cacaaatact
gcttctattg ctgaacatag aaacactgct gatgcaattg atacagtcga cagccatccg
aataatgaac ccagcaacaa caaacagcta aagccaccac aaatcgttgt gagtatcact
gatcttaatg atctatttga tatcattagt gaaatcacta gtattgacaa cgtatcagtc
aaagtcaacc aaggagtaac ggctagaata tttcctaatg atagcgaaac ttacagagct
attgtaagac attttgatgc aataggtatt gaatttcaca catatcagat gaaggaagaa
aaaccttaca gaattgtagt aaaggggctt caccatagta ctctgaataa tgaaataatt
gccaatttta aaaatatggc tttgatgctc tgcaaattca caacccaaga tcaaggtcaa
acagggatga aaaattgaat atattctttg taaatataaa accttgcaaa aacattaacg
aagtttacaa tataaaaatg ctctgccgcc aaatggtgag agttgaaagg atgcgtaaaa
cttctgaaat tgtcatatgc acacgatgtc aagaatatgg ccatacagcc aaatactgtc
gccgccatcc aaactgtgca cgttgtggcg aggaccatcc cacaatctgt gcacacgatc
acaagatgaa ccgcctacct gcattcactg tggaggtaac cacatggcaa gttacaaagg
ctgccagtgg taccaagact tctattgacg ttcaactggc ccttctacga caataagaac
caacaaggcc gcaacagcta caacgtaagc aacagcatca accaaccaca aagttacc--
---------- ---------- ---------- --------aa cgggtacgca tctgctcaga
gtcgcataca caatatgcaa gcgcaagccc agctaactac acctatagga aatattccac
agcagcagca acaagttgat gtccaatctc tattggaaca acaacaacaa caatttctga
aatggcaaaa agagctgcaa gttcagcagc agcagcaatt tctctcgtgg cttcaggcgc
aacaacgcga gcaacaacaa caaaataaga aaaatagtga gcgactagaa cggctcgaaa
aaatggtcaa cgaaatggcc agcatgctca agcagtggac tgggggccca tcgactcacc
```

agcttcttaa caacgcctca gccacacaat gaactcacta aagatcctca tctggaatgc
caatggcatt gcaggaaaag ccagagatgt agagctattc gcgcacaaca acaacgtcga
tattcttcta ttaaatgaaa tcagactgaa tcgatgagat acagtaaaaa tatatggtta
taccttctat ccggcttaca aaccatcaag tcataaccat ggcatgggcg gagcggctat
attcatcaga agttctctgc gccacttccc acaaagagtt atcgaaactc aacacataca
aatgtctgca attaaagttg ccacaagatt gggagacgtt gaattttgcg ccatatattg
cccaccaagg aacagaattg aagaaaggca attcagtgac ataatcgctt cttgtggcca
aagatatttt attggtggcg actggaattc gcgacattgg ctgtggggag actcatacaa
ctctcccaga ggcagagaac ttgcagaagc cattacagcc agaggc---- --------gc
aacaggatct cctactagat acccgtatat atccagtcac actccctctt gcattgattt
tgcattatac catggggtac agcattacca agtaaatata taccaaaact gggacttgga
ttctgaccat ctagccctca ttgctgagtt gcacattgat ggctctaata tcagcccaag
cccaaggcta ataactaacc gtactgacat tgcggctttc agacaacagc tagatgactc
catccaatta aatccgtgtc tgaactcagg cgaagacatt gagaatgcgg tgatcttctc
tcggagaaaa tatatagagc tgctgctgct acaacgccga caaacaccgc gtttcgtccc
aaattatggc attgttctaa ctagagaagc ---------- ------caaa aaggttcctt
cgtagaagag caattcgatc ccaagatcct tgggaccgaa ttttgtggaa ccgtgcagca
agacagctac gcaatctcct aagagaaatc agagatgact tcttcgagca aaaactggct
tccatggact acactacgga tgcaaactac tcactatgga aatgcacaaa atcgcttaaa
cgacaaccat ttaggtgg-- ---------- ---------- ---------- ----------
---------- ---------- ---------- ---------- ---------- ----------
---------- ---------- ---------- ---------- ---------- ----------
---------- ---------- ---------- ---------- ---------- ----------
---------- ---------- ---------- ---------- ---------- ----------
---------- ---------- ---------- ---------- ---------- ----------
---------- ---------- ---------- ---------- ---------- ----------
---------- ---------- ---------- ---------- ---------- ----------
---------- ---------- --------tg aaacacatct tgcaggcttt tgatgactca
gaatactcca acgctgtctt catcgacacg caacaagcgt ttgataaggt gtggcacgat
ggattactgt gcaaaataaa aaaccttcta cctgctccgt actatggcct cttaaaatgt
tatctagaag accgtatgtt ccaagtcaag ataaaggata ctctctcgtc cacataccct
atgagagcag gtgtgccgca gggcagtgtc cttggtccgc tgctgttctc cctatatact
gcagacatac ctagcccgtc ctctcaacat atggatgctc cctcgaaagc tgtcatggcc
acatatgctg atgtcatagc aatcatatac aactcaaaaa accttgcaga atcaagcact
ggactgcaga ggtatctggg cactctcaat gattggtgta agcggtggaa tctcaaaatt
aacccgctaa aaacaatgaa cacttgcttc actctaaaaa gactgccaat gcataccccc
ccaatccaga tgtcgggagt aaatcttcag caacctgcac aagtgaaata tcttggcatc
acactggaca agcgtctcac ctttgggcca cacctcaaag ccacggtaaa aaaatgtcgt
cacagactgc aacaactaag atggctcaac aataaaaaga gcaccttacc gctgaga---
---------- ---------- ---------- ---------- ---------- ----------
---------- ---------- ---------- ---------- ---------- ----------
---------- ---------- ---------- ---------- ---------- ----------
---------- ---------- ---------- ---------- ---------- ----------
---------- -
>Dkoep352
gcagcccaga aaatgcttaa aaataaaagg aagaaacctc tatctcccta taaaaataat
aaaagagcaa gcagagaagt gtcccactcc gcttcaacta ctatggctaa atcaagtaat
gcctttgcat tactggatat ggacatggac gtaaccagtg atgtcgagaa tattgataat
catgtgattg ctgatcatag taatgctgct gatgcaattg acacaggcaa caaccaccta
aatcaagaac ccgacaacaa caaaaagcca aagccaccgc aaatcgttgt gagcatcact
gatcttaatg acctatttga aatcatcagt gaggtcacta gcgttgacaa cgtttcagtc
aaagtcaacc aaggagtaac ggctagaata tttgccaaag atagcgacac ctacagagct
atagtaagcc attttgatac aataggtatt gagtctcaca cataccaaat gaaggaagaa
aaaccttaca gaatcgtagt aaaagggctt caccatagta ctctgaatca agaaataatt
gccaatttta aaaatatggc tttgatgctc tgcaagtgca caacccaaga tcaaggtcaa
acagagagga aaaattaaat atattcttcg taaatataaa accttgcaaa aaaattaacg
atatttacag tataaaaact ctttgtcggc aaatggtgag agtagaaagg atgcgtaaag
cttccgaaat tgttatatgc acacgatgcc aggaatatgg tcattcagcc aaatactgcc
gtcgccatcc aaactgtgca cggtgtggcg aggaccatcc cacattctgt gcacacgatc
acaagatgaa ccgcctacct gcattcactg tggaggtaac cacatgacaa gctataaagg
ctgtcagtgg taccaagact tttatcgacg atcaactggt ccttctacga caagaagaac
caacaaggcc gcaacagcta caacgtaagc aacagcatca accaaccaca aagttaccaa
atacatctgg aggtcgatcc tatgccgcaa tagcaagaaa cggatacgta tctgctcaga
gtcgcataca caatatccaa gcgcaagccc agctagctac acctatagga aatattccac

```
agcagcagca acaagttgat gtccaatctc tattggaaca acaacaacaa caatttctga
aatggcaaaa agagctgcaa gttcagcagc agcagcaatt tctttcatgg cttcaggcgc
aacaacgcga acaacaacaa caaaataaga gaaatagtga tcgactagaa cggctcgaaa
gaatggtcca cgaaatggcc agcatgctca aacaatggac tgggggccca acgactcgcc
agcttcttaa caacgcctca gcctcacaat gagctcacta aagatcctca tttggaatgc
caatggcatt gcaggaaaag ccagagatgt agagctattc gcgcacaaca acaacgtcga
cattctactt ttaaatgaaa ttagactgaa tcgaggagat acagtcaaaa tctatggcta
taccttctat ccggcttaca aaccatcaag tcataaccat ggtatgggag gggcggctat
attcgtgaga aattctctgc gccacttccc acaaagagtt atcgaaactc aacacataca
aatgtctgca attaaagctg ccacaggatt gggagacgtt gaattctgcg ccatatactg
cccaccaagg gacagaattg aagaaaggca attcagcgac atactcgctt cttgtggcca
aaggtatttt attggtggcg actggaatgc gcgacattgg ctgtggggag actcatacaa
ctctcccagg ggcagagaac ttgcagaagc cattacagcc agaggc---- --------gc
aacaggatct cctactagat acccgtatat atccagtcac actccctctt gcattgattt
tgcattatac catgggatac agcattacca agtaaatata taccaaaact gggacttgga
ttctgaccat ctagccctca ttgctgagtt gcacattgat ggctctaata tcagcccaag
cccaaagcta ataactaacc gtactgacat tgcggctttc agacaacagc tagatgactc
catccaatta aatccgtgtc tgaactcagg cgaagacatt gagaatgcgg tgatcttctc
tcggagagaa tatatagagc tgctgctgct acaacgccga caaacaccgc gtttcgtccc
aaattatggc attgttctaa ctagagaagc ---------- ------caaa aaggttcctt
cgtagaagag caattcgatc ccaagatcct tgggaccgaa ttttgtggaa ccgtgcagca
agacagctac gcaatctcct aagagaaatc agacatgact tcttcgagca aaaactggct
tccatggact acactacgga tgcaaactac tcactatgga aatgcacaaa atcgcttaaa
cgacaaccat ttaggtgg-- ---------- ---------- ---------- ----------
---------- ---------- ---------- ---------- ---------- ----------
---------- ---------- ---------- ---------- ---------- ----------
---------- ---------- ---------- ---------- ---------- ----------
---------- ---------- ---------- ---------- ---------- ----------
---------- ---------- ---------- ---------- ---------- ----------
---------- ---------- ---------- ---------- ---------- ----------
---------- ---------- ---------- ---------- ---------- ----------
---------- ---------- --------tg aaacacatct tgcaggcttt tgatgactca
gaatactcca acgctgtctt catcgacacg caacaagcgt ttgataaggt gtggcacgat
ggattactgt gcaaaataaa aaaccttcta cctgctccgt actatggcct cttaaaatgt
tatctagaag accgtatgtt ccaagtcaag ataaaggata ctctctcgtc cacataccct
atgagagcag gtgtgccgca gggcagtgtc cttggtccgc tgctgttctc cctatatact
gcagacatac ctagcccgtc ctctcaacat atggatgctc cctcgaaagc tgtcatggcc
acatatgctg atgacatagc aatcatatac aactcaaaaa accttgcaga atcaagcact
ggactgcaga ggtatctgga cactctcaat gattggtgta agcggtggaa tctcaaaatt
aacccgctaa aaacaatgaa cacttgcttc actctaaaaa gactgccaat gcataccccc
ccaatccaga tgtcgggagt aaatcttcag caacctgcac aagtgaaata tcttggcatc
accctggaca agcgtctcac ctttgggtcg cacctcaaag ctacagttaa aaaatgtcgt
cacagactgc aacaattgag atggctcaac aataaaaaga gcaccttacc gctgagaaat
cgaa------ ---------- ---------- ---------- ---------- ----------
---------- ---------- ---------- ---------- ---------- ----------
---------- ---------- ---------- ---------- ---------- ----------
---------- ---------- ---------- ---------- ---------- ----------
---------- -
```
