# Supplementary material for: Expression of the Retrotransposon Helena Reveals a Complex Pattern of TE Deregulation in Drosophila Hybrids
Source: PLoS One. 2016 Jan 26;11(1):e0147903. doi: 10.1371/journal.pone.0147903 (PMC4728067; doi:10.1371/journal.pone.0147903)
Supplement: S2 Text — Highly conserved residues (similarity score per position > 0.5) are framed in blue and used to build the consensus sequence. Each nitrogenous base in a conserved position is represented in a different colour. (PDF) [file pone.0147903.s009.pdf]

```
Dsechellia      1  GATGCGCAAAAAACGAAAGAGAGGAAGAGGAAGACCAATTTAACACCCAACCATAGCTCTAAACGAGCTGTCAGAGACGCGCCATAT
Dyakuba            .......................................................................................
Derecta            .......................................................................................
Dananassae      1  ..................................................TATGAACTCTAAAAAGGTGCATAGAGAACTGTCACCC
Dmojavensi      1  GCAGCCCAGAAAATGCTTAAAACTAAAGGAAGATACCTCTATCTCCCAATAACAATAACAAAAGAGCAAACAGAGTTGAGTCACAC
Dvirilis        1  ................TGAAAAATAAGAGGAAGAAACCATTATCTCCCAATACTAGCTCTAAGAGGCCGAATAAAKATTTGTCGCCC
Dsimulans       1  GATGCGCAAAAAACGAAAGAGAGGAAGAGGAAGACCAATTTAACACCCAACCATAGCTCTAAACGAGCTGTCAGAGACGCGCCATAT
Dmelano            .......................................................................................
Dbuzzatii       1  GCAGCCCAGAAAATGCTTAAAAATAAAGGAAGAAACCTATATCTCCCTATAAAAATAATAAAAGAGCAAGCAGAGAAGTGTCCCAC
Dkoep28         1  GCAGCCCAGAAAATGCTTAAAAATAAAGGAAGAAACCTCTATCTCCCTATAAAAATAATAAAAGAGCAAGCAGAGAAGTGTCCCAC
Dkoep351        1  GCAGCCCAGAAAATGCTTAAAACTAAAGGAAGAAACCTCTATCTCCCTATAACAATAATAAAAGTGCAAGCAGAGATGAGTCACAC
Dkoep352        1  GCAGCCCAGAAAATGCTTAAAAATAAAGGAAGAAACCTCTATCTCCCTATAAAAATAATAAAAGAGCAAGCAGAGAAGTGTCCCAC
consensus>50       g..gc.ca.aaaa.g.t.aaaa.taa.aggaaga.acct.tatctccc.ataa.aa...taaaagagc.a.cagaga.g.gtc.cac

Dsechellia     88  CCAGCCATAAATAGTAATTCAAACTCAAACAATAGGTTTGCCATGCTAGACATGGAATTGGACGAAACCAGTGATGGCATAGATTGT
Dyakuba            .......................................................................................
Derecta            .......................................................................................
Dananassae     38  TCAGTATCAATCAGCCAGGAAAGCTCCAGCAATAGATTTGCTTTACTAGACATGGAAGTGGACCTAAACAGTATTGCTGAGGATGTA
Dmojavensi     88  TCCGCTTCAACTAATATGGCAAAATCCAGTAACAGCTTTGCTTTACTGGACATGGATATGGACGCAACCAGTGATGATGTGAAC...
Dvirilis       72  TCAGCATCAACAAGTATGGCAATCTCTGGCAACAGGTTTGCCTTGCTAGACATGGAMATGGACATGGATGTAACCCATGTTGATCCA
Dsimulans      88  CCAGCCATAAATAGTAATTCAAACTCAAACAATAGGTTTGCCATGCTAGACATGGAATTGGACGAAACCAGTGATGGCATAGATTGT
Dmelano            .......................................................................................
Dbuzzatii      88  TCCGCTTCAACTAATATGGCTAAATCAAGTAATGCCTTTGCTTTACTGGATATGGACATGGACGTAACCAGTGATGTCGAGAATATT
Dkoep28        88  TCCGCTTCAACTAATATGGCTAAATCAAGTAATGCCTTTGCATTACTGGATATGGACATGGACGTAACCAGTGATGTCGAGAATATT
Dkoep351       88  TACGCTCCAACTAATATAACAAAATCCAGTAATAGCTTTGCTTTACTAGACACAGACATGGACGTAACCAACGATGATGTGAACACA
Dkoep352       88  TCCGCTTCAACTACTATGGCTAAATCAAGTAATGCCTTTGCATTACTGGATATGGACATGGACGTAACCAGTGATGTCGAGAATATT
consensus>50       tc.gc.tcaacta.tatggcaaa.tc.ag.aatag.tttgc.ttact.gacatgga.atggacgtaaccagtgatg..g.g.at...

Dsechellia    175  CATACCTCGTGTGCATCGGCTGCTAATGATGACTGTGTACCAAATGTGACATCCAATAGCCCACAACAGTATAC......TGATAAG
Dyakuba            .......................................................................................
Derecta            .......................................................................................
Dananassae    125  GAGAGATCTCCTATGGCTGTTCATGACGATAATGTAGACACAGATGATTTAGGCAATAGATCCCAACAGAATGATTCTAATAGAAAT
Dmojavensi    172  ...ACTGCTTCTATTGTTGATCATAGCAACACTGCTGATGCAATTGATACAGTCAACAGCCATCAGAATAAAGAACCCAGCAACAAC
Dvirilis      159  AATAGCGCTTCTGAGGCTGATCATAACGTCAGTATTGATGTAAGTGACACAGCCAATMACCCACAGCATAACGCAATCAATACCAAC
Dsimulans     175  CATACCTCGTGTGCATCGGCTGCTAATGATGACTGTGTACCAAATGTGACATCCAATAGCCCACAACAGTATAC......TGATAAG
Dmelano            .......................................................................................
Dbuzzatii     175  GATAATCATGTGATTGCTGATCATAGTAATGCTGCTGATGCAATCGACACAGGCAACAACCACCTAAATCAAGAACCCGACAACAAC
Dkoep28       175  GATAATCATGTGATTGCTGATCATAGTAATGCTGCTGATGCAATTGACACAGGCAACAACCACCTAAATCAAGAACCCGACAACAAC
Dkoep351      175  AATACTGCTTCTATTGCTGAACATAGAAACACTGCTGATGCAATTGATACAGTCGACAGCCATCCGAATAATGAACCCAGCAACAAC
Dkoep352      175  GATAATCATGTGATTGCTGATCATAGTAATGCTGCTGATGCAATTGACACAGGCAACAACCACCTAAATCAAGAACCCGACAACAAC
consensus>50       .ata...ct..tat.gctgatcata...at..tg.tgatgcaa.tga.acag.caa.a.cc..c.a.at.a.gaa.cc...aacaac

Dsechellia    256  CAGAATTCAAAACCACCGCAAATAGTACTGAGCCTTACCAATCTTAATGATCTCTATGAGCTCATTACGGAGGTCACTAGCCTAGAT
Dyakuba            .......................................................................................
Derecta            .......................................................................................
Dananassae    212  TATTATCCAAAACCACCGCAAATAGTTTTAAGCATTGCTAACCTAAATGATCTATTTGACCTCATTTCGGAGGTCACTAGCTTGGAT
Dmojavensi    256  CAACAGACTAAGCCACCGCAAATCGTTGTGAGTATCACTGATCTTAATGATCTATTTGAAATCATTAGTGAAGTCACCAGTATTGAT
Dvirilis      246  GACCAGCTAAAGCCACCGCAAATAGTTGTGTGTGTTACTGACCTTAATGATCTTTTTGAACTCATTATGGAGGTCACTAGTCTAGAT
Dsimulans     256  CAGAATTCAAAACCACCGCAAATAGTACTGAGCCTTACCAATCTTAATGATCTCTATGAGCTCATTACGGAGGTCACTAGCCTAGAT
Dmelano            .......................................................................................
Dbuzzatii     262  AAAAAGCCGAAGCCACCGCAAATCGTTGTGAGCATCACTGATCTTAATGACCTATTTGAAATCATCAGTGAGGTCACTAGCGTTGAC
Dkoep28       262  AAAAAGCCAAAGCCACCGCAAATCGTTGTGAGCATCACTGATCTTAATGACCTATTTGAAATCATCAGTGAGGTCACTAGCGTTGAC
Dkoep351      262  AAACAGCTAAAGCCACCACAAATCGTTGTGAGTATCACTGATCTTAATGATCTATTTGATATCATTAGTGAAATCACTAGTATTGAC
Dkoep352      262  AAAAAGCCAAAGCCACCGCAAATCGTTGTGAGCATCACTGATCTTAATGACCTATTTGAAATCATCAGTGAGGTCACTAGCGTTGAC
consensus>50       .a..agccaaagccaccgcaaat.gttgtgagcat.actgatcttaatgatctatttga..tcatta..gaggtcactagc.t.ga.

Dsechellia    343  AATTTAACAGTTAAAGTCAATCAAGGGGAAACAGTGAGAATATTACCCAAAGACTCTGATACTTACAGAGCCATTATTAATATTTTT
Dyakuba            .......................................................................................
Derecta            .......................................................................................
Dananassae    299  AACGTAACAGTTAAAGTCAATCAAGGAGTTACGGCTAGAATATTTCCCAAGGACTCTACAACTTATAGAGCTATTGTCGACCATTTT
Dmojavensi    343  AACGTATCAGTCAAGATCAACCAAGGAGTGACGGCTAGAATATTTCCCAAAGATAGCGACACTTACAGAGCTATCGTAAGCCATTTT
Dvirilis      333  AACGTGGCAGTGAAAGTCAACCAAGGAGAAACGGCCAGAATATTTCCCAAAGACAGTGACACTTACAGAGCAATTGTCAAACATTTT
Dsimulans     343  AATTTAACAGTTAAAGTCAATCAAGGGGAAACAGTGAGAATATTACCCAAAGACTCTGATACTTACAGAGCTATTGTTAATCTTTTT
Dmelano            .......................................................................................
Dbuzzatii     349  AACGTTTCAGTCAAAGTCAACCAAGGAGTAACGGCTAGAATATTTGCCAAAGATAGCGTCACCTACAGAGCTATAGTAAGCCATTTT
Dkoep28       349  AACGTTTCAGTCGAAGTCAACCAAGGAGTAACGGCTAGAATATTTGCCAAAGATAGCGACACCTACAGAGCTATAGTAAGCCATTTT
Dkoep351      349  AACGTATCAGTCAAAGTCAACCAAGGAGTAACGGCTAGAATATTTCCTAATGATAGCGAAACTTACAGAGCTATTGTAAGACATTTT
Dkoep352      349  AACGTTTCAGTCAAAGTCAACCAAGGAGTAACGGCTAGAATATTTGCCAAAGATAGCGACACCTACAGAGCTATAGTAAGCCATTTT
consensus>50       aacgt..cagt.aaagtcaaccaaggagtaacggctagaatatttcccaaaga.ag.ga.acttacagagctat.gt.a..catttt
```

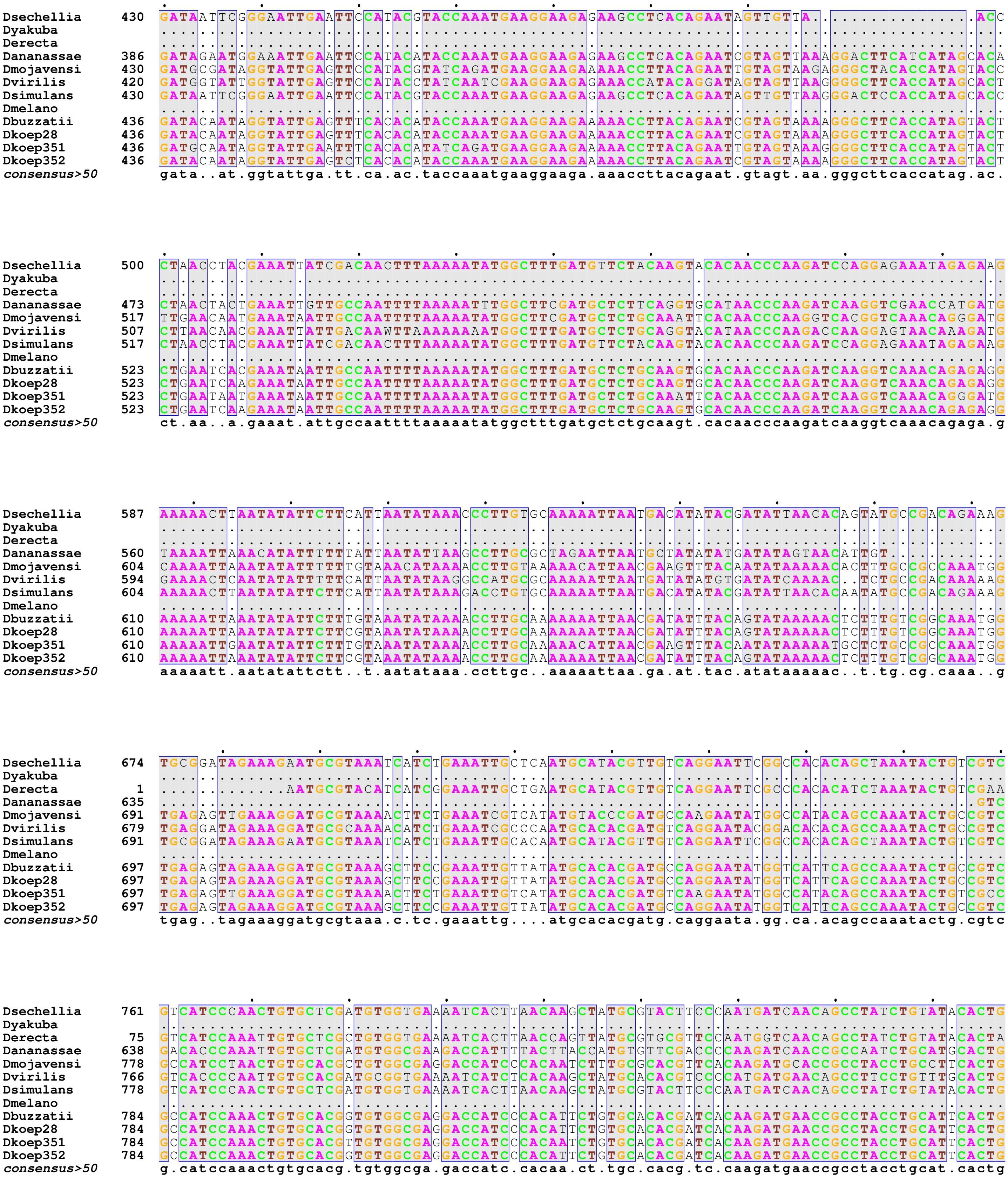

```
Dsechellia      848  TGGAGGAAATCACACGGCAAGTTACAAGGGTTGCCAGTTTTACCAGGAGTATCTTCGACGATCATTGGGCACTGTAAAGACAGGTTT
Dyakuba              .......................................................................................
Derecta         162  TGGAGGAAATCACACGGCAAGTTACAACGGTTTCCAGTGGTAACAGGAGTATCTTCGACGATCAATGGGCACTACAAAAACAGGTTA
Dananassae      725  TGGAGGCAATCATTCGGCAAGCTACAAGGGTTGTCAGTCCTATCAAAATTACCTCAGGCGATCTATGGGTTCTGCAAAGA.......
Dmojavensi      865  TGGAGGTAACCACATGGCAAGCTACAAAGGCTGCCCGTGGTACCAGGAGTTCTATCGACGTTCACTGGGCTCCTCTACGACAAGAAG
Dvirilis        853  TGGAGGGAATCATATGGCAAGCTACAAAGGTTGCCAATGGTACCAGGACTATCTACGACGATCAATGGGCTCTTCAATGACAGGAGG
Dsimulans       865  TGGAGGAAATCACACGGCAAGTTACAAGGGTTGCCAGTTTTACCAGGAGTATCTTCGACGATCAATGGGCACTGTAAAGACAAGTTT
Dmelano              .......................................................................................
Dbuzzatii       871  TGGAGGTAACCACATGGCAAGCTATAAAGGCTGTCAGTGGTACCAAGACTTTATCGACGTTCAACTGGTCCTTCTACGACAAGAAG
Dkoep28         871  TGGAGGTAACCACATGACAAGCTATAAAGGCTGTCAGTGGTACCAAGACTTTATCGACGATCAACTGGTCCTTCTACGACAAGAAG
Dkoep351        871  TGGAGGTAACCACATGGCAAGTTACAAAGGCTGCCAGTGGTACCAAGACTTCTATTGACGTTCAACTGGCCCTTCTACGACAATAAG
Dkoep352        871  TGGAGGTAACCACATGACAAGCTATAAAGGCTGTCAGTGGTACCAAGACTTTATCGACGATCAACTGGTCCTTCTACGACAAGAAG
consensus>50         tggagg.aa.cacatggcaagctacaaagg.tgccagtggtacca.ga.t.t..tcgacgatcaatgggc.cttc.a.gacaaga.g

Dsechellia      935  GATAAACAAACCAACAACAACCCCAGCAAAAAC.............AGCAGCATCAATAGCTAGCACTCCCAAAGGCTTGTCCTACG
Dyakuba              .......................................................................................
Derecta         249  GATTAACAAACCAGCAATAACCTCAGCAAAAACAGCATCATGTA.AAGCAGCATCAATAGCAAGCACTCCCAAAGGTTTTTCCTACG
Dananassae      805  ...CAACAACGCGACAACAACCACAACAACGTCATCAGCAACAACCGACAACTAATACTCCACATATTTCTGCAAGTTTATCTTATG
Dmojavensi      952  AATCAACAAGACCACAACAGCCACAACGGAAGCACCAGCATCAACCAACCACAAAGCTATCAAATACTTCTGGAGGAATATCCTATG
Dvirilis        940  AATCAACAAACCAGAAACAACCACAAAGGAAACAGCAGCAGCAGCCGACAGCAAAAATACCTAACATTCCTAGAGGCAAGTCCTATG
Dsimulans       952  GATAAACAAACCAACAACAACCCCAGCAAAAAC.............AGCAGCATCAATAGCTAGCACTCCCAAAGGCTTGTCCTACG
Dmelano              .......................................................................................
Dbuzzatii       958  AACCAACAAGGCCGCAACAGCTACAAGGTAAGCAACAGCATCAACCAACCACAAAGTTACCAAATACATCTGGAGGTCGATCCTATG
Dkoep28         958  AACCAACAAGGCCGCAACAGCTACAACGTAAGCAACAGCATCAACCAACCACAAAGTTACCAAATACATCTGGAGGTCGATCCTATG
Dkoep351        958  AACCAACAAGGCCGCAACAGCTACAACGTAAGCAACAGCATCAACCAACCACAAAGTTACC..........................
Dkoep352        958  AACCAACAAGGCCGCAACAGCTACAACGTAAGCAACAGCATCAACCAACCACAAAGTTACCAAATACATCTGGAGGTCGATCCTATG
consensus>50         aa.caacaa..c.gcaaca.ccacaacg.aa.ca.cagcatcaaccaac.acaaa..taccaaa.act.ct..agg....tcctatg

Dsechellia     1009  CAGATATTGCAAAAAATGGCAATACAACAGCCCAGCCTCGTCTACATAATGTAC.....................AATTAAAGGGAA
Dyakuba              .......................................................................................
Derecta         335  CAGCTATTGCAAGAAATGGCAATACAACAGACAAGCCTCGTCTACATAACGTACATGCCC...............AATTAAAGGAAT
Dananassae      889  CTTCAATAGTAAGAAATGGAAATGAACCAGCTCAACGCCGTCTACATGATCTCCAAGT.....CACAAGTACACAAATCGAAGGAAA
Dmojavensi     1039  CCGCGGTAGCAAGAAATGGAAGCGCATCTGCTCAAAGCCGCATACATAATATTCAAGCGCAAGCCCAGTTAGCTAAACCTATAGGAC
Dvirilis       1027  CCTCAATAGCAAGGAACGGCAACGAACCTGCCCAGCGCCGTCTACATAGTTTGCAAGCGCAACAACAGCTACTTAAGCGAATAGAAA
Dsimulans      1026  CAGATATTGCAAGAAATGGCAATACAACAGCCCAGCCTCGTCTACATAATGTAC.....................AATTAAAGGGAA
Dmelano              .......................................................................................
Dbuzzatii      1045  CCGCAATAGCAAGAAACGGATACGTATCTGCTCAGAGCCGCATACACAATATCCAAGCGCAAGCCCAGCTAGCTACACCTATAGGAA
Dkoep28        1045  CCGCAATAGCAAGAAACGGATACGTATCTGCTCAGAGTCGCATACACAATATCCAAGCGCAAGCCCAGCTAGCTACACCTATAGGAA
Dkoep351       1019  ..............AACGGGTACGCATCTGCTCAGAGTCGCATACACAATATGCAAGCGCAAGCCCAGCTAACTACACCTATAGGAA
Dkoep352       1045  CCGCAATAGCAAGAAACGGATACGTATCTGCTCAGAGTCGCATACACAATATCCAAGCGCAAGCCCAGCTAGCTACACCTATAGGAA
consensus>50         c.gc.atagcaagaaa.gg.aacg.a.ctgctcag.gtcg..tacataat.t.caagcgcaa.c.cag.ta..taaacc.ataggaa

Dsechellia     1075  CTAATATTAAGCAGCAACACCCGCTTGACGTTCAGTCAATATTGGCACAGCAACAGGAACAATTTATGAAGTGGCAGCAACAGCTTC
Dyakuba              .......................................................................................
Derecta         407  ATAATATTAAACAGCAACGCCCGATTAACGTTCAGTCAATATTGGCACAGCAGCAGGAACAATTTATGAATTGGC............
Dananassae      971  ATGT...GCAACAACAATATGCGACTGATGTTCAGGCAATTTTAGAGCAACAGCAACAGCTATTTATGAAATGGCAACAACAACTCC
Dmojavensi     1126  ATATCGCACAGCAGCAGCATCAAGTTGATGTCCAATCTTTACTGGAACAACAACAACAACAATTTTTGAAATGGCAAAAAGACTTGC
Dvirilis       1114  ATATCAGGCAGCAGCATCATCCAGCTGACGTTCAATCATTATTGGAACAACAGCAACAGCAATTTT....AATGCAACAACAGCTGC
Dsimulans      1092  CTAATATTAAGCAGCAACACCCGCTTGACGTTCAATCAATATTGGCACAGCAACAGGAACAATTTATGAAGTGGCAGCAACAGCTTC
Dmelano              .......................................................................................
Dbuzzatii      1132  ATATTCCACAGCAGCAGCAACAAATTGATGTCCAATCGCTATTGGAACAACAACAACATCAATTTCTGAAATGGCAAAAAGAGCTGC
Dkoep28        1132  ATATTCCACAGCAGCAGCAACAAGTTGATGTCCAATCTCTATTGGAACAACAACAACAACAATTTCTGAAATGGCAAAAAGAGCTGC
Dkoep351       1092  ATATTCCACAGCAGCAGCAACAAGTTGATGTCCAATCTCTATTGGAACAACAACAACAACAATTTCTGAAATGGCAAAAAGAGCTGC
Dkoep352       1132  ATATTCCACAGCAGCAGCAACAAGTTGATGTCCAATCTCTATTGGAACAACAACAACAACAATTTCTGAAATGGCAAAAAGAGCTGC
consensus>50         atatt...cagcagca.ca.c.a.ttgatgt.caatc..tattggaacaacaacaacaacaattt.tgaaatggcaa.aa.agctgc

Dsechellia     1162  AACAGCAACAACAGCAGCAATTCCTATCGTGGCTACTACAGCAGCAACAGGAGCAACAACAACACAACAAGTTGAATAGTCAACGAC
Dyakuba              .......................................................................................
Derecta         482  ......AGCAACAGCAGCAATTCCTATCGTGACT............ACAGGAGCAACAACAACAAAACAAGCTGAATAGCCAACGAC
Dananassae     1055  AAGAAAAGCAGCAGCAGCAATTCCTTATGTGGCTAAGGCAGCAGCATCAAGAACAACAAATGCAAAACAAGCTGAACAGTCAACGAC
Dmojavensi     1213  AAGTGCAGCAACAGCAGCAATTTCTTTCATGGCTTCAAGCACAACAACGCGAACAGCAACAACAAAACAAGAGAAACAGTGAACGAC
Dvirilis       1197  AACTACAGCAGCAGCAGCAATTCCTCTTGTGGCTTCAGGAGCAGCAACGGGAACAACAACAACAAAACAAGCAAAATAGTGAACGGC
Dsimulans      1179  AACAGCAACAACAGCAGCAATTCCTATCGTGGCTACAGCAGCAGCAACAGGAGCAACAACAACAAAACAAGTTGAATAGTCAACGAC
Dmelano              .......................................................................................
Dbuzzatii      1219  AAGTTCAGCAGCAGCAACAATTTCTTTCATGGCTTCAGGCGCAACAACGCGAACAACAACAACAAAATAAGAGAAATAGTGATCGTC
Dkoep28        1219  AAGTTCAGCAGCAGCAGCAATTTCTTTCATGGCTTCAGGCGCAACAACGCGAACAACAACAACAAAATAAGAGAAATAGTGATCGAC
Dkoep351       1179  AAGTTCAGCAGCAGCAGCAATTTCTCTCGTGGCTTCAGGCGCAACAACGCGAGCAACAACAACAAAATAAGAAAAATAGTGAGCGAC
Dkoep352       1219  AAGTTCAGCAGCAGCAGCAATTTCTTTCATGGCTTCAGGCGCAACAACGCGAACAACAACAACAAAATAAGAGAAATAGTGATCGAC
consensus>50         aagt.cagcagcagcagcaatt.ct.tcgtggcttcagg.gca.caacg.gaacaacaacaacaaaacaag..aaatagtgaacgac
```

```
Dsechellia     1249  TCGAAAGGCTGGAAAAAATTGTTTTTGAAATGGCCAATATGCTGAAGCAACGGACTGGGGATACATCGGCTCCCCAACTCCATAGTA
Dyakuba              .......................................................................................
Derecta         551  TCGAACGGTTGGAAAAACTGGTTTTTGAAATGGCCAATATGCTGAAGCAATGGACTGGGGATGCATCGGCTCTCCAACTCCATTGTA
Dananassae     1142  TTGAACGTCTTTAAAATATGGTTTTTGAAATGGCAAACTCGATCAAGCAATGGACTGGAGATAAATCACTTCTTCAGCTTCCCAACA
Dmojavensi     1300  TAGAACGTCTAGAAAAAATGGTCCACGAAATGGCCAGCATGCTCAAGCAATGGACTGGGGATTCATCGACTCACCAGCTTCTTAACA
Dvirilis       1284  TAGAACGGCTGGAAAAAATGGTCCATGAAATGGCCAGCATGATAAAGCAATGGACAKGGGATGGATCGACTCCCCAGCTTCATAGCA
Dsimulans      1266  TCGAAAGGCTGGAAAATATTGTTTTTGAAATGGCCAATATGCTGAAGCAACGGACTGGGGATACATCGGCTCCCCAACTCCATAGTA
Dmelano              .......................................................................................
Dbuzzatii      1306  TAGAACGGCTCGAAAAAATGGTCCACGAAATGGCCAGCATGCTCAAGCAATGGACTGGGGGCCCAATGACTCGCCAGCTTCCTAACA
Dkoep28        1306  TAGAACGGCTCGAAAAAATGGTCCACGAAATGGCCAGCATGCTCAAACAATGGACTGGGGGCCCAACGACTCGCCAGCTTCTTAACA
Dkoep351       1266  TAGAACGGCTCGAAAAAATGGTCAACGAAATGGCCAGCATGCTCAAGCAGTGGACTGGGGGCCCATCGACTCACCAGCTTCTTAACA
Dkoep352       1306  TAGAACGGCTCGAAAGAATGGTCCACGAAATGGCCAGCATGCTCAAACAATGGACTGGGGGCCCAACGACTCGCCAGCTTCTTAACA
consensus>50         tagaacggct.gaaaaaatggtc.a.gaaatggccagcatgctcaagcaatggactggggat.catcgactc.ccagcttc.taaca
```

```
Dsechellia     1336  ACGATTCACCATCGCAATTAACCCTCTGAAGATTCTTATCTGGAATGTAAATGGTTTTTCAGGTAAAGCCAGAGAAGTCGAGCTCTT
Dyakuba              .......................................................................................
Derecta         638  ACGCTTTACCATCGCAATGAACCCTCTGAAGATACTTATCTGGAATGTAAATAGTATTTCAGGTAAAGCCAGAGAAGTTGAGCTCTT
Dananassae     1229  ACGCTTCAGCCTCACAATGAACCCACTCAAAATTCTTATTTGGAACGTCAATGGCATTTTAGGTAAAGCCAGAGAAATTGAATTCTT
Dmojavensi     1387  ACGCCTCAGCCTCACAATGAACCCACTCAAGATTCTCATCTGGAATGTCAATGGCATTGCAGGAAAAGCCAGAGATGTGGAGCTATT
Dvirilis       1371  ACGCCTCAGCCTCCCAATGAATCCACTCAAGGTCCTTATCTGGAATGTTAATGGCATCGCAGGAAAAGCCAGTGAAGTTGAGCTCTT
Dsimulans      1353  ACGCTTTACCATCGCAATGAACCCTCTGAAGATTCTTATCTGGAATGTAAATGGTATTTCAGGTAAAGCCAGAGAAGTAGAGCTCTT
Dmelano              .......................................................................................
Dbuzzatii      1393  ACGCCTCAGCCTCACAATGAGCTCACTAAAGATTCTCATTTGGAATGTCAATGGCATTGCAGGAAAAGCCAGAGATGTAGAGCTATT
Dkoep28        1393  ACGCCTCAGCCTCACAATGAGCTCACTAAAGATCCTCATTTGGAATGCCAATGGCATTGCAGGAAAAGCCAGAGATGTAGAGCTATT
Dkoep351       1353  ACGCCTCAGCCACACAATGAACTCACTAAAGATCCTCATCTGGAATGCCAATGGCATTGCAGGAAAAGCCAGAGATGTAGAGCTATT
Dkoep352       1393  ACGCCTCAGCCTCACAATGAGCTCACTAAAGATCCTCATTTGGAATGCCAATGGCATTGCAGGAAAAGCCAGAGATGTAGAGCTATT
consensus>50         acgcctcagcctcacaatgaacccact.aagat.ct.atctggaatgtcaatggcattgcaggaaaagccagaga.gt.gagct.tt
```

```
Dsechellia     1423  CGCACACAACAACGGCATTGACATTCTTCTCCTAAACGAGATCAGACTCAACAGAGGGAACACAGTTAAGATATATGGGTATAGCTT
Dyakuba              .......................................................................................
Derecta         725  CACACACAACAACTGCGTTGACATTATTCTCCTAAACGAGATCAGACTCAACAGAGGGAACACCGTTAAGATACATGGATATAGCTT
Dananassae     1316  CGCGCACACCAACGAAGTTGATATCCTACTCCTAAGTGAATTAAGGCTCAATCGAGGAGAAATTGTCAAAATTTATGGATATTCCTT
Dmojavensi     1474  CGCGCACAACAACAACATCGACATTCTACTATTAAATGAAATCAGACTGAATCGAGGAGATACAGTCAAAATATATGGCTACACCTT
Dvirilis       1458  CGCGCACAACAACAATGTCG.............AAGTGAAATCAGACTAAACCGAGGAGAAACAGTCAAAATACACGGGTACACCTT
Dsimulans      1440  CGCACACAACAACGGCATTGACATTCTTCTCCTAAACGAGATCAGACTCAACAGAGGGAACACAGTTAAGATATATGGATACAGCTT
Dmelano              .......................................................................................
Dbuzzatii      1480  TGCGCACAACAACAACGTCGACATTCTACTTTTAAATGAAATTAGACTGAATCGTGGAGATACAGCCAAAATCTATGGCTATACCTT
Dkoep28        1480  CGCGCACAACAACAACGTCGACATTCTACTTTTAAATGAAATTAGACTGAATCGAGGAGATACAGTCAAAATCTATGGCTATACCTT
Dkoep351       1440  CGCGCACAACAACAACGTCGATATTCTTCTATTAAATGAAATCAGACTGAATCGATGAGATACAGTAAAAATATATGGTTATACCTT
Dkoep352       1480  CGCGCACAACAACAACGTCGACATTCTACTTTTAAATGAAATTAGACTGAATCGAGGAGATACAGTCAAAATCTATGGCTATACCTT
consensus>50         cgcgcacaacaacaacgtcgacattct.ct..taaatgaaatcagact.aatcgaggaga.acagtcaaaatatatgg.tatacctt
```

```
Dsechellia     1510  TTATCCCGCATACAAACCTTCAAGCCATAATCACGGAATGGGAGGAGCAGCAGTACTGGTGAGAAGCTCTCTTCGTCATTTCCCGCA
Dyakuba              .......................................................................................
Derecta         812  TTATCCCGCATACAAACCATCAAGCCATAATCATGGAATGGGGGAGCAGCAGTACTGGTGAGGAGCTCTCTTCGTCATTTCCCGCA
Dananassae     1403  TTATCCAGCATACAAACCGGCAAGACACAATCACGGCACCGGAGGAGCGGCGGTATTGGTAAGGAGCTCTCTTAGTCATTTTCCACA
Dmojavensi     1561  CTATCCAGCTTACAAGCCATCAAGCCACAACCATGGTATGGGAGGAGCGGCTATATTCGTTAGAAGTTCACTGCGCCACTTCCCACA
Dvirilis       1532  CTATCCAGCTTACAAGCCATCACGTCACAGTCACGGTGTCGGAGGAGCGGCGGTATTCGTGAGGAGTACTCTCCGCCATTTTCCACA
Dsimulans      1527  TTATCCCGCATACAAACCTTCAAGCCATAATCACGGAATGGGAGGAGCAGCAGTACTGGTGAGAAGTTCTCTTCGTCATTTCCCGCA
Dmelano              .......................................................................................
Dbuzzatii      1567  CTATCCGGCTTACAAACCATCAAGTCATAACCATGGTATGGGAGGAGCGGCTATATTCGTGAGAAATTCTCTGCGCCACTTCCCACA
Dkoep28        1567  CTATCCGGCTTACAAACCATCAAGTCATAACCATGGTATGGGAGGGGCGGCTATATTCGTGAGAAATTCTCTGCGCCACTTCCCACA
Dkoep351       1527  CTATCCGGCTTACAAACCATCAAGTCATAACCATGGCATGGGCGGAGCGGCTATATTCATCAGAAGTTCTCTGCGCCACTTCCCACA
Dkoep352       1567  CTATCCGGCTTACAAACCATCAAGTCATAACCATGGTATGGGAGGGGCGGCTATATTCGTGAGAAATTCTCTGCGCCACTTCCCACA
consensus>50         ctatcc.gcttacaaaccatcaag.cataa.catgg.atgggaggagcggc..tattcgtgagaagttctct.cgcca.ttcccaca
```

```
Dsechellia     1597  AAGAGTTATTGAAACGAGAACTATTCAGATGTCTTCAGTCAAGGTCTCCACCGGGCTGGGAGATATGGAATTTAGCGCGATTTACTG
Dyakuba              .......................................................................................
Derecta         899  AAGAGTTATTGAAACGAGAACTATTCAGATGTCTTCAGTTAAGGTTTCCACCGGGCTGGGAGATATGGAATTTAGCGCGATATACTG
Dananassae     1490  AAGTGTTATTGAAACACAAACCATTCAAATGTCCTCTATCAAAGTTGACACAGGTTTGGGTATCATGGTAGTATGCGCAATATACTG
Dmojavensi     1648  AAGGGTTATTGAAACTCAAAATATACAAATGTCTGCAATCAAAGTTGCCACTGGATTGGGAGACATCGAATTTTGCGCCATATACTG
Dvirilis       1619  AAGAGTAATTGAAACGAGCACCATACAAATGTCTTCAATAAAAATTGCCACAGGTTTGGGAGAGATGGAATATAGCGCCATATACTG
Dsimulans      1614  AAGAGTTATTGAAACGAGAACTATTCAGATGTCTTCAGTCAAGGTCTCCACCGGGCTGGGAGATATGGAATTTAGCGCGATTTACTG
Dmelano              .......................................................................................
Dbuzzatii      1654  AAGAGTTATCGAAACTCAACATATACAAATGTCTGCAATTAAAGTTGCCACAGGATTGGGAGACGTTGAATTCTGCGCCATATACTG
Dkoep28        1654  AAGAGTTATCGAAACTCAACACATACAAATGTCTGCAATTAAAGCTGCCACAGGATTGGGAGACGTTGAATTCTGCGCCATATACTG
Dkoep351       1614  AAGAGTTATCGAAACTCAACACATACAAATGTCTGCAATTAAAGTTGCCACAAGATTGGGAGACGTTGAATTTTGCGCCATATATTG
Dkoep352       1654  AAGAGTTATCGAAACTCAACACATACAAATGTCTGCAATTAAAGCTGCCACAGGATTGGGAGACGTTGAATTCTGCGCCATATACTG
consensus>50         aagagttattgaaac.caaa..atacaaatgtct.caat.aaagttgccacagg.ttgggagacat.gaattttgcgccatatactg
```

```
Dsechellia     1684  TCCACCAACAAATAGAATTGAGGAAAGGCACTTCAGTGACATACTTGTCTCTTGTGGACAAAGGTATTTCGTTGGTGGGGACTGAAA
Dyakuba              .......................................................................................
Derecta         986  TCCACCAACAAATAGAATTGAAGAAAGGCACTTCAGTGACATACTTGTCTCTTGTGGCCAAAGGTATTTAGTTGGTAAGGACTGGAA
Dananassae     1577  TCCTCCAAGAAACAGAATTGAAGAAAGGCATTTCACTGACATACTCGCTTCTTGTGGTCAGAGGTATCTGGTCGGTGGTGACTGGAA
Dmojavensi     1735  CCCACCAAGGAACAGAATAGAAGAAAGACAATTCAGCGACATACTCGCTTCTTGCGGCCAAAGGTATCTTATTGGTGGTGACTGGAA
Dvirilis       1706  CCCACCAAGAAATAGAATTGAAGAAAGGCATTTCAGTGACATACTCGCTTCTTGCGGCCAAAGGTATCTGGTTGGTGGAGACTGGAA
Dsimulans      1701  TCCACCAAGAAATAGAATTGAGGAAAGGCACTTCAGTGACATACTTGTCTCTTGTGGACAAAGGTATTTCGTTGGTGGGGACTGGAA
Dmelano              .......................................................................................
Dbuzzatii      1741  CCCACCAAGGAACAGAATTGATGAAAGGCAATTCAGCGACATACTCGCTTCCTGTGGCCAAAGGTATTTTATTGGTGGCGACTGGAA
Dkoep28        1741  CCCACCAAGGAACAGAATTGAAGAAAGGCAATTCAGCGACATACTCGCTTCTTGTGGCCAAAGGTATTTTATTGGTGGCGACTGGAA
Dkoep351       1701  CCCACCAAGGAACAGAATTGAAGAAAGGCAATTCAGTGACATAATCGCTTCTTGTGGCCAAAGATATTTTATTGGTGGCGACTGGAA
Dkoep352       1741  CCCACCAAGGGACAGAATTGAAGAAAGGCAATTCAGCGACATACTCGCTTCTTGTGGCCAAAGGTATTTTATTGGTGGCGACTGGAA
consensus>50         cccaccaag.aacagaattgaagaaaggca.ttcagtgacatactcgcttcttgtggccaaaggtattt..ttggtgg.gactggaa

Dsechellia     1771  CGCCCGATATTGGCTATGGGGTGATACGTACAATTAACCCAGAGGTCGAGAACTAGCAGAAGCCATTTCAGCCAGAGGTGCTTATAT
Dyakuba              .......................................................................................
Derecta        1073  CATTAGCTATGATGTGACGGGTGATACGTACAAATCACCCACGGGTCCAGAACTAGCAGAAGCCATTTCAGCCAGAGGTGCTTATAT
Dananassae     1664  TGCTAGACATTGGATGTGGGGCGACACTTACAATTCACCAAGAGGACGCGAGCTAGCTGAATCCACTGTAGTAACAGGGGCTAAAAT
Dmojavensi     1822  TGCACGACACTGGCTGTGGGGAGATTCATACAATTCACCCAGGGGAAGAGAACTAGCAGAAGCCATCACGGCCAGAGGAGCTAATAT
Dvirilis       1793  TGCGCGACATTGGCTGTGGGGAGATACCTACAATTCACCCAGGGGCCGAGAACTAGCCGAAGCCATTACAGCCAGAGGAGCTAATAT
Dsimulans      1788  CGCCCGACATTGGCTATGGGGTGACACGTACAATTCACCCAGAGGTCGAGAACTAGCAGAAGCCATTTCAGCCAGAGGGGCTTATAT
Dmelano              .......................................................................................
Dbuzzatii      1828  TGCGCGACATTGGCTGTGGGGAGACTCATACAACTCTCCCAGGGGCAGAGAACTTGCAGAAGCCATTACAGCCAGAAGCGCTAATAT
Dkoep28        1828  TGCGCGACATTGGCTGTGGGGAGACTCATACAACTCTCCCAGGGGCAGAGAACTTGCAGAAGCCATTACAGCCAGAGGCGCTAATAT
Dkoep351       1788  TTCGCGACATTGGCTGTGGGGAGACTCATACAACTCTCCCAGAGGCAGAGAACTTGCAGAAGCCATTACAGCCAGAGGC........
Dkoep352       1828  TGCGCGACATTGGCTGTGGGGAGACTCATACAACTCTCCCAGGGGCAGAGAACTTGCAGAAGCCATTACAGCCAGAGGC........
consensus>50         tgc.cgacattggctgtggggagac.c.tacaa.tcacccagggg..gagaactagcagaagccattacagccagagg.gct.atat

Dsechellia     1858  CCTTGCAACAGGTTCACCAACTAGATACCCACATGTGCCCAGTCACAGACCTACCTGCATTGATTTTGCTGTGTACCATGGGATAAA
Dyakuba              .......................................................................................
Derecta        1160  CCTTGCAACAAGTTCACCAACTTGATACCCACATGTGCCCAGTCATAGACCTACCTGCATTGATTTTGCTGTGTACCATGGGATAAA
Dananassae     1751  TCTTGCCACTGGTTCACCAACTAGATACCCTTATGTACCTGGCCACACAGCTTCTTGCATAGACTTTGCATTATACCATGGGATACT
Dmojavensi     1909  TCTTGCAACAGGTTCTCCTACTAGGTACCCATATATATCCAGTCATACTCCCTCTTGCATTGATTTTGCATTATACCATGGGATACA
Dvirilis       1880  TCCCGCAACAGGATCACCAACTAGATATCCATATGTGTCTAGTCACACACCTAGTTGCATTGATTTCGCATTATACCATGGGATACC
Dsimulans      1875  CCTTGCAACAGGTTCACCAACTAGATACCCACATGTGCCCAGTCACAGACCTACCTGCATTGATTTTGCTGTGTACCATGGGATAAA
Dmelano              .......................................................................................
Dbuzzatii      1915  TCTTGCTACAGGATCTCCTACTAGATACCCGTATATATCCAATCACACTCCCTCTTGCATTGATTTTGCATTATACCATGGGATACA
Dkoep28        1915  TCTTGCTACAGGATCTCCTACTAGATACCCGTATATATCCAGTCACACTCCCTCTTGCATTGATTTTGCATTATACCATGGGATACA
Dkoep351       1867  ....GCAACAGGATCTCCTACTAGATACCCGTATATATCCAGTCACACTCCCTCTTGCATTGATTTTGCATTATACCATGGGGTACA
Dkoep352       1907  ....GCAACAGGATCTCCTACTAGATACCCGTATATATCCAGTCACACTCCCTCTTGCATTGATTTTGCATTATACCATGGGATACA
consensus>50         .cttgcaacagg.tc.cc.actagataccc.tat.tatccagtcacac.cc.tcttgcattgattttgcattataccatgggataca

Dsechellia     1945  CTTAGACAGAACTAGTATTTCTGAAAATTGGGATCTAGACTCCGATCATGTAGCCCTTGTGGCTACTGTACAAACAGAAGGTGCCTA
Dyakuba              .......................................................................................
Derecta        1247  CGTAGACAGAACAAGTATTTGCGAAATTTGGGATCTAAACTCCGATCATGTAGCCCGTGTTGCTACTCTACAAACAGAAGGTGTCTA
Dananassae     1838  GGATTCTCAAATAAGTATAGGCCAAAACTGGGATTTGGACTCGGATCACATAGCTCTCGTTGTTAATCTGCAAACAAATGGTGTACA
Dmojavensi     1996  GCATTATCAAGCAAATATTTACCAAAGCTGGGACTTAGACTCTGACCATTTAGCCCTTATTGCCGAGTTACACATCGATGGTCATTA
Dvirilis       1967  GGATTCTCAAACAAATATCGGCCAAAGCTGGGATCTAGACTCTGACCATATAGCCCT.GTTGTAAATTTGCAAACAGATGGTTCTTA
Dsimulans      1962  CTTAGACAGAACTAGTATTTCTGAAAATTGGGATCTAGACTCCGATCATGTAGCCCTTGTGGCTACTCTACAAACAGAAGGTGCCTA
Dmelano              .......................................................................................
Dbuzzatii      2002  GCATTACCAAGTAAATATACACCAAAACTGGGACTTGGATTCTGACCACCTAGCCCTCATTGCCGAGTTGCACATTGATGGTTCTAA
Dkoep28        2002  GCATTACCAAGTAAATATACACCAAAACTGGGACTTGGATTCTGATCACCTAGCCCTCATTGCTGAGTTGCACATTGATGGTTCTAA
Dkoep351       1950  GCATTACCAAGTAAATATATACCAAAACTGGGACTTGGATTCTGACCATCTAGCCCTCATTGCTGAGTTGCACATTGATGGCTCTAA
Dkoep352       1990  GCATTACCAAGTAAATATATACCAAAACTGGGACTTGGATTCTGACCATCTAGCCCTCATTGCTGAGTTGCACATTGATGGCTCTAA
consensus>50         g.attaccaa..aaatat.t.ccaaaactggga.tt.gactctga.cat.tagccct..ttgct.a.ttgca.a..gatggt.ct.a

Dsechellia     2032  TGTTAGACCATGCTCTCGGTTAATAAACAGCCGAACTGATCTCCTTGTTTCAGACAACATCTGGAAAACTCTCTCCAATTAAATAC
Dyakuba              .......................................................................................
Derecta        1334  TGTTACGCCATGTTCTCGGTTAAAAAA.....AAACCGATTTTTTTGTTTCAG...ACATCTTGAAAACTCTTTTCAATTAAATAC
Dananassae     1925  GATCAGACCAAGCCCTCGTTTAATTACAAGCCGAACTGATCTCAATACTTTCAAACAACATCTTGAGACCTCCTTTCAACTAAATTC
Dmojavensi     2083  TATCAGGCCAAGCCCAAGGCTAATAACCAGCCGTACTGACATCGAGGCTTTCAGACAACAGCTAGATGACTCCATTCATTTGAATTT
Dvirilis       2053  TGTCAGGCCAAACCCTCGATTAATTACCAACCGTACTGACATCATGGCTTTCAGACAACATCTTGAATGCTCGATTCAATTAAATCC
Dsimulans      2049  TGTTAGACCATGCTCTCGGTTAATAAACAGCAGAACTGATCTCCTTGTTTCAGACAACATCTGGAAAACTCTCTCCAATTAAATAC
Dmelano              .......................................................................................
Dbuzzatii      2089  TATCAGCCCAAGCCCAAGGCTAATAACCAACCGTACTGACATTGCGGCTTTCAGACAACAGCTAGATGACTCCATCCAATTAAATCC
Dkoep28        2089  TATCAGCCCAAGCCCAAGGCTAATAACTAACCGTACTGACATTGCGGCTTTCAGACAACAGCTAGATGACTCTATCCAATTAAATCC
Dkoep351       2037  TATCAGCCCAAGCCCAAGGCTAATAACTAACCGTACTGACATTGCGGCTTTCAGACAACAGCTAGATGACTCCATCCAATTAAATCC
Dkoep352       2077  TATCAGCCCAAGCCCAAAGCTAATAACTAACCGTACTGACATTGCGGCTTTCAGACAACAGCTAGATGACTCCATCCAATTAAATCC
consensus>50         tatcag.ccaagccc..gg.taataac.a.ccgtactgacat...ggctttcagacaaca.ct.ga..actc.atccaattaaat.c
```

```
Dsechellia     2119  GGTTCTGAGCTCTAAGGAAGATATCGAGAACGCAGTCGCAGTCTAACGCAAAATATATATATAGCCGCTTCTGCTTCTAC.......
Dyakuba              .......................................................................................
Derecta        1413  GGTTCTCAGCTCTAAGGAAGAGATCAAGAACCCAGTGACATCCGAACGCAAAATATACATAGAGCCGCTTCTGTTTCCACGCCGTCT
Dananassae     2012  TGTTCTGAACTCTAGAGAAGACATCGAGAATGCAGTGACATTCTTACTGATAATATATATAGAGCTGCTACTGTTTCAACGCCACCA
Dmojavensi     2170  GTGTCTGAACTCCGGCGAAGACATCGAGAATGCGGTGATAATCTCTCGGAGAACATATATAGAGCTGCTGCTGCTACAACGCCGCTT
Dvirilis       2140  GACCTTGGACTCTGGAGTAGACATTGAGAATGCAGTGATTTTCTAACTGAAAATATACATAGCGCTGCTACTGCCACAACTCCACAC
Dsimulans      2136  GGTTCTGAGCTCTAAGGAAGACATCGAGAACGCAGTGACAGTCTAACGCAAAATATACATAGAGCCGCTTCTGCTTCTACGCCGTCT
Dmelano              .......................................................................................
Dbuzzatii      2176  GTGTCTGAACTCAGGTGAAGATATCGAGAATGCGGTGATCTTCTCGCGGAGAACATATATAGAGCTGCTGCTGCTACAACGCCGATA
Dkoep28        2176  GTGTCTGAACTCAGGTGAAGATATTGAGAATGCGGTGATCTTCTCTCGGAGAACATATATAGAGCTGCTGCTGCTACAACGCCGATT
Dkoep351       2124  GTGTCTGAACTCAGGCGAAGACATTGAGAATGCGGTGATCTTCTCTCGGAGAAAATATATAGAGCTGCTGCTGCTACAACGCCGACA
Dkoep352       2164  GTGTCTGAACTCAGGCGAAGACATTGAGAATGCGGTGATCTTCTCTCGGAGAGAATATATAGAGCTGCTGCTGCTACAACGCCGACA
consensus>50         g..tctgaactc.gg.gaagacatcgagaatgc.gtgat.ttct..cgga.aa.atatatagagctgct.ctgctacaacgccg...

Dsechellia     2199  ..GCCCGAGATACGCCCCAAGTTATGGTATTGTTCTAACAAGAGAGGCAGATAACTTATCAGAACTAAGAGACGCCTTCGAAGAAGA
Dyakuba              .......................................................................................
Derecta        1500  GAGCCTGAGACATGCCCCATGTTATGGCATTGTACTAACAAGAGAGGCAGAGAACTAATCAGAACTAAAAGACGTCTTCGAAGAAGA
Dananassae     2099  GAACCTGTTCTTCGTCCTAAGCTATGGCATTGTTCTTACAAGGGAGGCAGAGAGTTAATCAAGAGAAAAAGGCGCCTTCGAAGAAGA
Dmojavensi     2257  AATCCTGCGCCTCGTCCCAAATTATGGCATTGTTCTAACGAGAGAAGCAAAGAACTCATCAGAACAAAAAGGCGCCTTCGTAGAAGA
Dvirilis       2227  ATCACCGAGTCACGTCCCACGTTATGGCATTGTTTTCACGAGGGAGGCAAAGAGCTGATCAGAACAAAGAGGTGCCTTCGAAGAAGA
Dsimulans      2223  GAGCCCGAGATACGCCCCAAGTTATGGTATTGTTCTAACAAGAGAGGCAGAGAACTTATCAGAACTAAGAGACGCCTTCGAAGAAGA
Dmelano              .......................................................................................
Dbuzzatii      2263  AACACCGCGTTTCGTCCCAAATTATGGCATTGTTCTAACAAGAGAAGCAAAGAACTTATCAGAACAAAAAGGTTCCTTCGTAGAAGA
Dkoep28        2263  AACACCGCGTTTCGTCCCAAATTATGGCATTATTCTAACAAGAGAAGCAAAGAACTTATCAGAACAAAAAGGTTCCTTCGTAGAAGA
Dkoep351       2211  AACACCGCGTTTCGTCCCAAATTATGGCATTGTTCTAACTAGAGAAGC................CAAAAAGGTTCCTTCGTAGAAGA
Dkoep352       2251  AACACCGCGTTTCGTCCCAAATTATGGCATTGTTCTAACTAGAGAAGC................CAAAAAGGTTCCTTCGTAGAAGA
consensus>50         aa..ccg.g.ttcgtcccaa.ttatggcattgttctaacaagaga.gca.agaact.atcagaacaaaaagg.gccttcg.agaaga

Dsechellia     2284  GCAATTCGAACTCAGGATCCATGGGACAGAATCTTGTGGAACCGAGCAGCAAAGCAACTCAAAGTCATCTTAAGGGAACTCAGAAGT
Dyakuba           1  ...................CCATGGGACAGAATCTTGTGGAACCGAGCAGCAAAGCAACTGAAAGGCATTCTAAGGGAGCTCAGAAAT
Derecta        1587  GCAATTCGAACTCGGGATCCATGGGACAGAATCTTGTGGAACCGAGCAGCAAAGCAACTCAAAACCATCTTAAGGGAACTCAGAAGT
Dananassae     2186  GCAATTCGTTCTCAAGATCCTTTGGACCGAAATAGATGGCACCAGGCTGAGAGGCAACTACGAACTGTTTTAGATGAACTCCGAAGC
Dmojavensi     2344  GCAATTCGATCCCAAGATCCTTGGGACCGCCTTTTGTGGAACCGGGTAGCAAGACAGCTACGTAACCTTTTAAGGGAACTCAGAAGC
Dvirilis       2314  GCAATTCGATCACAAGATCCTTGGGACCGAATTTTGTGGAGCCGAGCAGCAAAACAGCTACGTAACCTCCTAAGGGAACTCAGAAGT
Dsimulans      2310  GCAATTCGAACTCAGGATCCATGGGACAGAATCTTGTGGAACCGAGCAGCAAAGCAACTCAAAGTCGTCTTAAGGGAACTCAGAAGT
Dmelano              .......................................................................................
Dbuzzatii      2350  GCAATTCGATCCCAAGATCCTTGGGACCGAATTCTGTGGAACCGTGCAGCAAGACAGCTACGCAATCTCTTAAGAGAAATCAGAGGT
Dkoep28        2350  GCAATTCGATCCCAAGATCCTTGGGACCGAATTCTGTGGAACCGTGCAGCAAGACAGCTACGCAATCTCTTAAGAGAAATCAGAGGT
Dkoep351       2282  GCAATTCGATCCCAAGATCCTTGGGACCGAATTTTGTGGAACCGTGCAGCAAGACAGCTACGCAATCTCCTAAGAGAAATCAGAGAT
Dkoep352       2322  GCAATTCGATCCCAAGATCCTTGGGACCGAATTTTGTGGAACCGTGCAGCAAGACAGCTACGCAATCTCCTAAGAGAAATCAGACAT
consensus>50         gcaattcgatc.caagatccttgggaccgaattttgtggaaccg.gcagcaagacagctacg.aacctcttaagggaactcagaagt

Dsechellia     2371  GATTTCTTTGAGCAAAAATTATCCTCCATGGACTACACCGTTGATGCAAACTATTCGCTGTGGAAGTGCACAAAAGCGCTTAAGCGA
Dyakuba          70  GATTTCTTTGAGCAAAAATTATCTTCCATGGACTACACAGTTGATGCAAACTATTCGCTGTGGAAGTGCACAAAAGCGCTCAAGAGA
Derecta        1674  TATTTCTTTGAGAAAAAAGTATCTTCCATGGACTACACTGTTGATGCAAACTATTCGCTGTGGAAGTGCACAAAAGCGCTTAAACAT
Dananassae     2273  GATTGTTTCGAGCAAAAGTTATCCTCTATGGACAATACCGTTGACGCAAACTACTCACTATGGAAGTGCACAAAATCGCTTAAACGA
Dmojavensi     2431  GATTTCTTCGAGCAAAAACTGGCTTCCATGGACTACACTATTGATGCAAACTATTCGTTATGGAAATGCACAAAATCACTCAAACGA
Dvirilis       2401  GAATTTCTT...CGAAAACTGGCTTCCATGGACTACACCGTTGATGCGAACTACTCTCTACGGAAATGTACAAAATCACTCAAACGA
Dsimulans      2397  GATTTCTTTGAGCAAAAATTATCCTCCATGGACTACACCGTTGATGCAAACTATTCGCTGTGGAAGTGCACAAAAGCCCTTAAACGA
Dmelano              .......................................................................................
Dbuzzatii      2437  GACTTTTTCGAGCAAAAACTGGCTTCCATGGACTACACTACAGATGCAAACTACTCACTATGGAAATGCACAAAATCGCTTAAAAGA
Dkoep28        2437  GACTTTTTCGAGCAAAAACTGGCTTCCATGGACTACACTACAGATGCAAACTACTCACTATGGAAATGCACAAAATCGCTTAAACGA
Dkoep351       2369  GACTTCTTCGAGCAAAAACTGGCTTCCATGGACTACACTACGGATGCAAACTACTCACTATGGAAATGCACAAAATCGCTTAAACGA
Dkoep352       2409  GACTTCTTCGAGCAAAAACTGGCTTCCATGGACTACACTACGGATGCAAACTACTCACTATGGAAATGCACAAAATCGCTTAAACGA
consensus>50         gatttcttcgagcaaaaactggcttccatggactacactgttgatgcaaactactc.ctatggaaatgcacaaaatcgcttaaacga

Dsechellia     2458  CAACCACTTCGATGGGTACCCGTACGCTGTCCAGGTGGGGAATTTGCAAAAAGAAGTGGAACAGGCTAATGCATTCGGCTTCCACCT
Dyakuba         157  CAACCATTTCGATGGGTACCAGTACGATGTCCTGGCGGGCAAATTGCAAAAAGAAGTGGAACAGGCTAATGCATTCGGCTACCACCT
Derecta        1761  CAACCACTTAGATGGGTACCCG....................................................TCGGCTACCACCT
Dananassae     2360  CAACCTTTTAGACAAGTTCCAGTCCGATGTCCTGATGGTGAACTGGCAAAAAGAATTGGAGCAGGCTAATGCTTTTGGCCAGCATCT
Dmojavensi     2518  CAACCTTTTAGACAGGTACCTGTCCGATGCCCTAACGGCGAAGTTGCTAAATGAATTGGAACAGGCTAACGCATTCGGATGTCATCT
Dvirilis       2485  CAACCCTTTAGACAGGTACCTGTCCGATGTCCTGGTGGCGAACTTGCGAACA...................................
Dsimulans      2484  CAACCACTTCGATGGGTACCCGTACGCTGTCCAGGTGGGGAATTTGCAAAAAGAAGTGGAACAGGCTAATGCATTCGGCTTCCACCT
Dmelano              .......................................................................................
Dbuzzatii      2524  CAACCATTTAGACAGGTACCTGTTCGATGCCCTAGTGGCGAACTTGCTAAAAGAATTGGAACAGGCTAACGCATTCGGAAGCCATCT
Dkoep28        2524  CAACCATTTAGACAGGTGCCTGTTCGATGCCCTAATGGCGAACTTGCTAAAAGAATTGGAACAGGCTAACGCATTCGGAAGCCATCT
Dkoep351       2456  CAACCATTTAGGTGG........................................................................
Dkoep352       2496  CAACCATTTAGGTGG........................................................................
consensus>50         caaccatttagatgggtacc.gt.cgatg.cct..tgg.gaa.ttgc.aaaagaa.tggaacaggctaa.gcattcgg...cca.ct
```

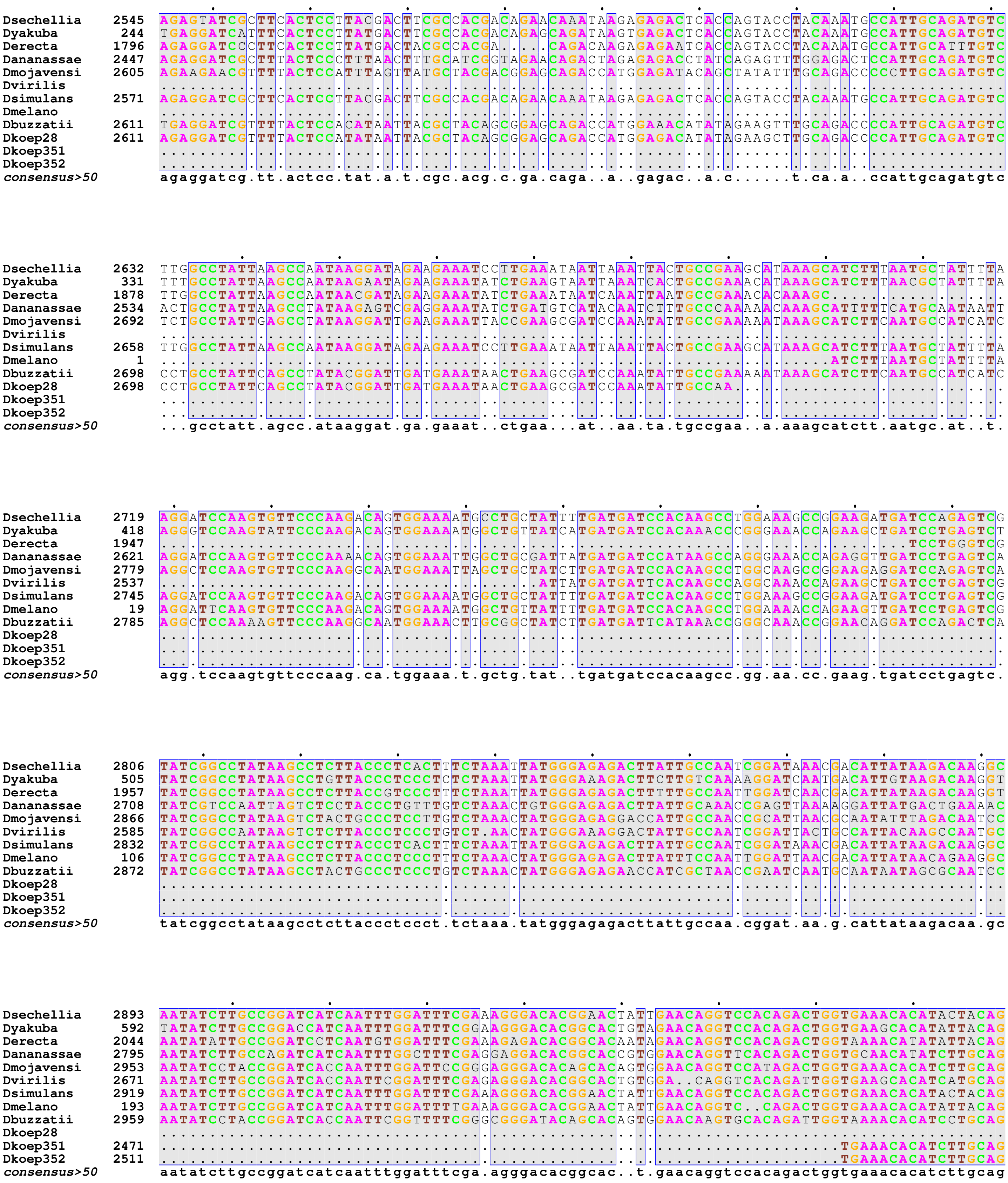
Dsechellia
Dyakuba
Derecta
Dananassae
Dmojavensi
Dvirilis
Dsimulans
Dmelano
Dbuzzatii
Dkoep28
Dkoep351
Dkoep352
consensus>50

```
Dsechellia     2980  GCTTTTGACGACTGCGAGTACTCCAACGCCGTCTTTATAGATATGCAACAAGCCTTCGACAAAGTATGGCATGTTGGATTATTATGC
Dyakuba         679  GCTTTTGACGACTGCGAATACGCAAACGCTGTCTTTATAGATATGCAACAAGCCTTCGACAAAGTATGGCATGTTGGATTATTATGC
Derecta        2131  GCTTTTGACGACTGCGAGT..........CGTCTTTATAGACATGCAACAAGCCTTCGACAATGTATGGCATGTTTGATTATTATGC
Dananassae     2882  GCCTTTGACGATCAGGAGTATTCCAACGCAGTTTTTATCGATATGCAGCAAGCTTTTGATAAGGTGTGGCATGATGGCTTATTGCTC
Dmojavensi     3040  GCTTTTGATGACTCTGAATACTCCAATGCTGTCTTTATCGACATGCAGCAAGCGTTTGATAAGGTGTGGCACGATGGATTACTGTAC
Dvirilis       2756  GCTTTTGACGACCTAGAGTTCTCAAACGCTGTCTTCATTGACATGCAGCAAGCGTTCGATAAGGTGTGGCACGATGGATTACTGTGC
Dsimulans      3006  GCTTTTGACGACTGCGAGTACTCCAACGCCGTCTTTATAGATATGCAACAAGCCTTCGACAAAGTATGGCATGTTGGATTATTATGC
Dmelano         278  GCTTTTGACGACTACGAGTACTCAAACGCTGTCTTTATAGATATGCAACAAGCCTTCGACAAAGTATGGCATGTTGGATTATTGTGC
Dbuzzatii      3046  GCCTTTGATGACTTAGAATACTCCAACGCTGTCTTCATTGACTTGCAGCAAGCGTTTGACAAGGTGTGGCACGATGGATTACTGTGC
Dkoep28              .......................................................................................
Dkoep351       2488  GCTTTTGATGACTCAGAATACTCCAACGCTGTCTTCATCGACACGCAACAAGCGTTTGATAAGGTGTGGCACGATGGATTACTGTGC
Dkoep352       2528  GCTTTTGATGACTCAGAATACTCCAACGCTGTCTTCATCGACACGCAACAAGCGTTTGATAAGGTGTGGCACGATGGATTACTGTGC
consensus>50         gcttttgacgact..gagtactccaacgctgtctttat.gacatgcaacaagc.ttcgacaaggtgtggcatgatggattattgtgc

Dsechellia     3067  AAGATAAAGACCCTTCTACCTGCGCCCTACTTCTGTATTTTAAAGTCATATCTAGAAGAAAGAGAATTTAAAATCACGGTGAGAAAT
Dyakuba         766  AAGATAAAGACCATGCTTCCTGCGCCCTACTTCTGTATTTTAAAGTCATATCTAGAGGGACGAGAATTTAAAATCACAGTTAGGAAT
Derecta        2208  TAGATGAAGACCCTCCTACCTGCGCCCTACTTCTGTATTTTGAAGTCATATCT..........GAATTTAAAATCACGGTGAGAAAT
Dananassae     2969  AAAATTAAAAATCTTTTACCTGCGCCGTACTATGGTCTGTTTAGATCATATTTAGAAGTACGAGAATTCAAGGTGAAAGTAAAAGAT
Dmojavensi     3127  AAAATAAAAAACCTTCTACCTGCTCCGTACTATGGCCTCTTAAGATCTTATCTAGAAGACCGTGTGTTCCAAGTCAAGGTAAAAGAT
Dvirilis       2843  AAAATAAAAAACCTTTTACCTGCACCGTACTATGGCCTTCTAAGGTCATATCTGGAAGTACGAGAGTTCAAGGTCAAGGTAAGGGAC
Dsimulans      3093  AAGATAAAGACCCTTCTACCTGCGCCCTACTTCTGTATTTTAAAGTCATATCTAGAAGAAAGACAATTTAAAATCACGGTGAGAAAT
Dmelano         365  AAGATAAAGACCCTTCTACCTGCGCTCGACT....CATTTTAAAGTCATATCTGGAAGGACGACAATTTAAAATCTTAGTGAGAACT
Dbuzzatii      3133  AAAATAAAAAACCTTCTGCCTGCTCCGTACTATAGCCTCTTAAAATCCTATCTAGAAGACCGTGAGTTTAAAGTCAAGGTAAAGGAT
Dkoep28              .......................................................................................
Dkoep351       2575  AAAATAAAAAACCTTCTACCTGCTCCGTACTATGGCCTCTTAAAATGTTATCTAGAAGACCGTATGTTCCAAGTCAAGATAAAGGAT
Dkoep352       2615  AAAATAAAAAACCTTCTACCTGCTCCGTACTATGGCCTCTTAAAATGTTATCTAGAAGACCGTATGTTCCAAGTCAAGATAAAGGAT
consensus>50         aaaataaaaaaccttctacctgcgccgtactat.gcctttaaagtcatatctagaagaacgagaatttaaagtcaaggtaagagat

Dsechellia     3154  AGCTACTCCTCTATATACCCAATGAGAGCTGGAGTCCCTCAGGGCAGTGTTCTCGGACCGCTACTGTATTCCTTGTACACTGCTGAT
Dyakuba         853  AACTACTCTACCGTATATCCAATGAGAGCTGGAGTCCCACAGGGCAGCGTCCTTGGACCACTACTATACTCCTTGTACACTGCAGAT
Derecta        2285  AACTACTCTTCTATATATCCAATGAGAACTGGAGTCCCACAGGGCAGTGTTCTCGAACCGTTACTGTATTCCTTGTACACTGCTGAT
Dananassae     3056  TCATACTCGGACAACTTTTTGATGAGAGCAGGAGTTCCACAGGGAAGTGTGCTTGGACCGTTGTTGTACTCACTGTATACGGCAGAT
Dmojavensi     3214  ACACTCTCGTCCACATACCCTATGAGAGCAGGAGTGCCGCAGGGCAGTGTTCTTGGTCCGCTGCTGTTCTCCTTGTACACCTCTGAT
Dvirilis       2930  ACATACTCGTCCACCTATACTATGAGAGCAGGAGTGCCACAGGGCAGTGTTCTTGGTCCGCTGCTCTACTCGTTGTATACCTCAGAT
Dsimulans      3180  AGCTACTCCTCTATATACCCAATGAGAGCTGGAGTCCCTCAGGGCAGTGTTCTCGGACCGCTACTGTATTCCTTGTACACTGCTGAT
Dmelano         448  AACTACTCCTCTATATACCCAATGAGAGCTGGAGTCCCACAGGGCAGTGTTCTCGGACCGCTACTATATTCCTTGTACACTGCTGAT
Dbuzzatii      3220  ACACTCTCGTCCACATATCCTATGAGAGCAGGAGTTCCGCAGGGCAGTGTTCTTGGTCCGCTGCTGTTCTCCTTGTATACATCGGAC
Dkoep28              .......................................................................................
Dkoep351       2662  ACTCTCTCGTCCACATACCCTATGAGAGCAGGTGTGCCGCAGGGCAGTGTCCTTGGTCCGCTGCTGTTCTCCCTATATACTGCAGAC
Dkoep352       2702  ACTCTCTCGTCCACATACCCTATGAGAGCAGGTGTGCCGCAGGGCAGTGTCCTTGGTCCGCTGCTGTTCTCCCTATATACTGCAGAC
consensus>50         ac.tactcgtcca.ataccc.atgagagcaggagt.cc.cagggcagtgttcttggaccgctgctgtactccttgtacactgc.gat

Dsechellia     3241  ATCCCTTGCCCGAGTTTCGAACACATGGCAGCACCGAACAGGACTCTTATTGCAACCTATGCAGATGACATCGCAGTTGTATATAAC
Dyakuba         940  ATCCCTTGCCCGAACTTCGATCACATGGAAGCACCCTACAAGGCTCTTATGGCAACCTATGCAGATGACATCGCAGTGGTGTATAAC
Derecta        2372  ATCGCTTGCCTGAATTTCGAACACATGGTAGCACCCAACAAGGCTCTTATGGCAACCTATGCAGATGACATTGCAGTTGTGTACAAC
Dananassae     3143  ATACCTATCCCGAGCAGCCAACATATGGTAGCCCCCTCTAAAGCACTTATTGCGACCTATGCAGATGACATTGCAGTCATTTACAAC
Dmojavensi     3301  ATACCTAGCCCGTCCTCTCAACATATGGATGCTCCCTCGAAAGCTGTCATTGCCACATATGCTGATGATATTGCCATCATCTATAAC
Dvirilis       3017  ATACCCAGCCCTACCTCTCAACATATGGACGATCCTTCTAAGGCTATTATTGCGACATATGCAGATGACATCGCAGTCATTTATAAC
Dsimulans      3267  ATCCCTTGCCCGAGTTTCGAACACATGGCAGCACCGAACAGGACTCTTATTGCAACCTATGCAGATGACATCGCAGTTGTATATAAC
Dmelano         535  ATCCCTTGCCCGAGTTTCGAACACAGGGAAGCACCGAACAGGGCTCTTATTGCAACCTATGCAGATGACATCGCAGTTGTATATAAC
Dbuzzatii      3307  ATTCCTAGCCCGTCCTCCCAACATATGAATGCTCCCTCTAAAGCCGTCATTGCCACATATGCTGATGACATAGCAATCATATACAGC
Dkoep28              .......................................................................................
Dkoep351       2749  ATACCTAGCCCGTCCTCTCAACATATGGATGCTCCCTCGAAAGCTGTCATGGCCACATATGCTGATGTCATAGCAATCATATACAAC
Dkoep352       2789  ATACCTAGCCCGTCCTCTCAACATATGGATGCTCCCTCGAAAGCTGTCATGGCCACATATGCTGATGACATAGCAATCATATACAAC
consensus>50         at.cctagcccga.ct.ccaacatatggaagc.ccctc.aaggctcttattgc.acctatgcagatgacat.gcagtcatatataac

Dsechellia     3328  TCTAGGGACAGCAGAGAGACAGCTAACGGACTACAAGAATATATTAATGATCTGGCAGCCTGGTGTAAACGGTGGAACCTAAAAATA
Dyakuba        1027  TCTGGGGACAGCGGAGAGGCAGCGAAAGGACTGCAAGAATACATTAATGCTCTAGCAGCCTGGTGTAAACGGTGGAATCTAAAAATA
Derecta        2459  TCTAGGGACAGTAGAGAGACAGCTACAAGGATACATTAA................................................
Dananassae     3230  TACAGATGTCACAGAGAAGCTTCCAAAGGATTACAGGAGTACCTATTCACTCTTGCAGCTTGGTGCAAAAGATGGAACCTGAAGATC
Dmojavensi     3388  TCAAAAAACGTGGTTGAAGCAAGTACAGGACTACAGAGATATCTGGATACTCTCGCTGATTGGTGCAAGCGGTGGAATCTCAAAGTT
Dvirilis       3104  TCCAAAAGTTGCGTAGAAGCTAGCAATGGACTTCAGGGGTATCTGGATACTCTCGCAGCTTGGTGCAAACGGTGGAATTTGAAGGTT
Dsimulans      3354  TCTAGGGACAGCAGAGAGGCAGCTAACGGACTACAAGAATATATTAATGCTCTGGCAGCCTGGTGTAAACGGTGGAACCTAAAAATA
Dmelano         622  TCTAGGGACAGCAGAGAGGCAGCTAACGGACTGCAATAATATATTAATGCTCTGGCAGCCTGGTGTAAACGGTGGAACCTAAAAATA
Dbuzzatii      3394  TCAAAAAACCTTGCAGAATCAAGTATTGGACTACAGAGATACCTTGATACTTTCGCTGGTTGGTGCAAGCGGTGGAACCTAAAAGTT
Dkoep28              .......................................................................................
Dkoep351       2836  TCAAAAAACCTTGCAGAATCAAGCACTGGACTGCAGAGGTATCTGGGCACTCTCAATGATTGGTGTAAGCGGTGGAATCTCAAAATT
Dkoep352       2876  TCAAAAAACCTTGCAGAATCAAGCACTGGACTGCAGAGGTATCTGGACACTCTCAATGATTGGTGTAAGCGGTGGAATCTCAAAATT
consensus>50         tc.aga.ac.gcggagaagca.ctaa.ggactacag.aatatct..atactct.gcagcttggtgtaaacggtggaa.ct.aaaat.
```

```
Dsechellia     3415 AACCCACTGAAAACAACAAACCCGTGCTTCACGTTAAAAACGCTTATCCCGAACACCCCTCCAATTCGGCTAGAAGGAGTTACCCTG
Dyakuba        1114 AATGCAACGAAAACCACAAATCTATGTTTCACATTGAAAACGCTCATCAAAAAACACCCCCACTCCAGCTAGAAGGAGTTACACTA
Derecta             .......................................................................................
Dananassae     3317 AATCCGTAGAAAACAACGAACGTCTGCTTTACACTGAAGAGGCTCATAATTAATACCCCTCAAATCCAACTTGAGGGAGTTACCCTA
Dmojavensi     3475 AACCCGCTAAAAACATTGAACCCTTGCTTCACTCTAAAAAGGCTGGCAATGCATACCCCCCCAATCCAGATGTCGGGAGTAACCCTA
Dvirilis       3191 AACCCGTTAAAAACTATTAACCCATGCTTCACCCTCAAAATGCAG...ATGCATATCCCTCCAATTCAGCTTGAAGGAGTTACCCTG
Dsimulans      3441 AACCCACTGAAAACAACAAACCCGTGCTTCACGTTAAAAACGCTTATCCAAAACACCCCTCCAATTCGGCTAGAAGGAGTTACCCTG
Dmelano         709 AACCCACTGAAAACAACAAATCCATGCTTCACATTAAAAACGCTTGTCCAAAACACCCCTCCAATCCGGCTAGAAGGAGTTACCCTG
Dbuzzatii      3481 AACCCGCTAAAAACACTAAACCCCTGCTTCACCCTAAAAAGGTCGGCAATACATACCCCCCCAATCCAGATGTCCGGAGTAAATCTT
Dkoep28             .......................................................................................
Dkoep351       2923 AACCCGCTAAAAACAATGAACACTTGCTTCACTCTAAAAAGACTGCCAATGCATACCCCCCCAATCCAGATGTCGGGAGTAAATCTT
Dkoep352       2963 AACCCGCTAAAAACAATGAACACTTGCTTCACTCTAAAAAGACTGCCAATGCATACCCCCCCAATCCAGATGTCGGGAGTAAATCTT
consensus>50        aacccgct.aaaacaa..aaccc.tgcttcac.ctaaaaa.gct....at..atacccc.ccaatccagct.ga.ggagttaccct.

Dsechellia     3502 AATCAGACCCTGCAAGCAACACATCTAGGTATCACCCTGGATTAACGGCTCACCTTTGGGCCGCATCTAAAAAAAACAGTAAAAAAA
Dyakuba        1201 GACCAGCCGCTGCAAGCAACATATCTAGGTATAACACTGGATAAACGCCTCACCTTTGGGCCGCATCTCAAAAATGCAGTAAAGAAA
Derecta             .......................................................................................
Dananassae     3404 GAACAGCAAAACGAAGCAAAATATCTTGGTATTACTCTGGACAAGCGACTTACTTTCGGGCCACATCTGAAGTCAACAACTAAAAAA
Dmojavensi     3562 CAGCAACCTGCTCAGGTGAAATATCTTGGCATCACACTGGACAAGCGCCTCACCTTCGGTCCACACCTCAAAGCTACGGTGAAAAAA
Dvirilis       3275 GAGCAGCCGCCACAAGCTAAGTATCTCGGCATCACCTTAGACAAGCGCCTTACCTTTGGGCCACACCTCAAAGCTACGGTAAAAAAA
Dsimulans      3528 AATCAGCCCCTGCAAGCAACATATCTAGGTATCACCCTGGATAAACGGCTCACCTTTGGGCCGCATCTAAAAAACACAGTAAAAAAA
Dmelano         796 AATCAACCGCTGCAAGCAACATATCTAGGTATCACCGTCGATAAAAGGCTCACCTTTTGGTTGCATCTCAAAAACACAGTAAAGAAA
Dbuzzatii      3568 CAGCAACTTGCACAGGTGAAATATCTTGGCATCACACTGGACAAGCGTCTCACCTTTGGGCCACACCTCAAAGCCACGGTAAAAAAA
Dkoep28        2757 ...................................................................TCGAA...............
Dkoep351       3010 CAGCAACCTGCACAAGTGAAATATCTTGGCATCACACTGGACAAGCGTCTCACCTTTGGGCCACACCTCAAAGCCACGGTAAAAAAA
Dkoep352       3050 CAGCAACCTGCACAAGTGAAATATCTTGGCATCACCCTGGACAAGCGTCTCACCTTTGGGTCGCACCTCAAAGCTACAGTTAAAAAA
consensus>50        .a.ca.cc....caagc.aaatatct.gg.atcac.ctggacaagcg.ctcacctttgggcc.ca.ctcaaa.c.acagtaaaaaaa

Dsechellia     3589 TGTGGCCACAGATTACAACAGCTGAGATGGCACATGAATAGAAGGAGCACTCTTTCGATGAGGTGCAAAAGAGCTGTGTATGCGCAC
Dyakuba        1288 TGTGGTCGCAGATCACAACAACTGAGATGGCTCATGAACAGAAGGAACACCCTCTCGCTGAGATGCAAAAGAGCTGTGTATGCGCAC
Derecta             .......................................................................................
Dananassae     3491 TGTAATATGAGGGTTCAGCAGCTGCGTTGGATGATAAGCAAAAAGAAGTACCATGCCGCTTAGGTGCAAAAGGGCAGTATATGTTCAT
Dmojavensi     3649 TGTCGTCACAGACTGCAACAATTAAGATGGCTCACCAATAGAAAGAGCACCTTACCGCTGAGATGCAAAAGAGCTGTCTATGTGCAC
Dvirilis       3362 TGTCGCCACAGACTGCAACAACTGCGGTGGCTCAACAATAAAAGGAGCACCTTGCCGCTGAGATGCAAAAGAGCTGTATACGTGCAC
Dsimulans      3615 TGTGGCCACAGATCACAACAGCTGAGATGGCTCATGAATAGAAGGAGCACTCTTTCGATGAGGTGCAAAAGAGCTGTGTATGCGCAC
Dmelano         883 TGTGGTCACAGATCACAACAGCTGAGATGGCTGATGAATAGAAGGATCACTCTTTCGCTGAGGTGCAAAAGAGCTGTGTATGCGCAC
Dbuzzatii      3655 TGTCGTCACAGACTGCAACAACTAAGATGGCTCAACAATAAAAAGAGCACCTTACCGCTGAG.........................
Dkoep28             .......................................................................................
Dkoep351       3097 TGTCGTCACAGACTGCAACAACTAAGATGGCTCAACAATAAAAAGAGCACCTTACCGCTGAGA........................
Dkoep352       3137 TGTCGTCACAGACTGCAACAATTGAGATGGCTCAACAATAAAAAGAGCACCTTACCGCTGAGAAATCGAA.................
consensus>50        tgt.gtcacaga.t.caacaactgagatggctca..aata.aaggagcacc.t.ccgctgag.tgcaaaagagctgt.tatg.gcac

Dsechellia     3676 TGTATCGTACCGATATGGTTATACGGGATCCAGATTTGAGGAATTGCAGCCAAATCGAATTATAATCGTATCCAGGTCTGCCCCTGG
Dyakuba        1375 TGTATCCTACCGATCTGGTTATACGGGATCCAGATTTGGGGAATCGCAGCCAAATCAAATTACAAACGAATCCAGGTCTGCCCCTGG
Derecta             .......................................................................................
Dananassae     3578 TGCATCCTGCCAATGTGGCTTTATGGTGTACAAATTTGGGGGATCGCCGCCAAATCGAACTATAAGCGAATCCAGGTCTGTCCCTGG
Dmojavensi     3736 TGCATCCTACCAATATGGCTCTATGGAGTGCAAATCTGGGGGATCGCTGCAAAATCGAATTATAAAAGAGTGCAGGTCTGTCCCTGG
Dvirilis       3449 TGTATTTTGCCGATATGGCTCTATGGAGTGCAGATTTGGGGGATTGCAGCCAAATCAAATTATAAACGCATACAGGTCTGCCCCTGG
Dsimulans      3702 TGTATCGTACCGATATGGTTATACGGGATCCAGATTTGGGGAATTGCAGCCAAATCGAATTATAATCGTATCCAGGTCTGCCCCTGG
Dmelano         970 TGTATCGTACCGATGTAGTTATACGGGATCCAGATTTGGGGAATTGCAGCCGAATCTAATTATAAACGTATCCAGGTCTGTTCCTGG
Dbuzzatii           .......................................................................................
Dkoep28             .......................................................................................
Dkoep351            .......................................................................................
Dkoep352            .......................................................................................
consensus>50        tg.atc.t.cc.at.tgg.t.ta.gg..t.ca.atttgggg.at.gc.gccaaatc.aattataa.cg.at.caggtctg.ccctgg

Dsechellia     3763 TATGTACCTAACTCTACACTCCATAAAGACCTCAATATTCACACAGTTGAGTCACAAATTGGGAGACATACATGTCGATATAGTGAC
Dyakuba        1462 TACGTACGGAACTCTACACTCCATAAGGACCTCAATCTCTACACAGTTGAGGAACAAATTGGGAGACACACAAGTCGATACAGTGAC
Derecta             .......................................................................................
Dananassae     3665 TACGTGCGTGGCTCCACTCTTCATAGAGACCTTAACATACATACTGTGGAAGAACAGATAAATAGACACACAAGCCGTTACAGCGAC
Dmojavensi     3823 TACATACGCGGCTCAACACTGCATAGAGACCTCAAGCTGTCTACTGTCGAAGAACAAATAAATAAACACACAAGCAGATACTCAGAC
Dvirilis       3536 TACGTACGCGGTTCTACACTCCATAAGGACCTCAAAGTGCATACAGTCGAAGAACAGATTGGAAGGCACACAAGCAGATACAGCGAC
Dsimulans      3789 TATGTACGTAACTCTACACTCCATAAAGACCTCAATATTCACACAGTTGAGTCACAAATTGGGAGACATACAAGTCGATATAGTGAC
Dmelano        1057 TATGTACGTAACTCCACACTCTATAAAGACCTCAATATTCACACAGTTGAGACACAAATTGGGAGACATACAAGTCGATACAGTGGC
Dbuzzatii           .......................................................................................
Dkoep28             .......................................................................................
Dkoep351            .......................................................................................
Dkoep352            .......................................................................................
consensus>50        ta.gtacg...ctc.acact.cata..gacctcaa..t..a.ac.gt.ga...aca.at....agaca.acaag..gata.ag.gac
```

```
                        .         .         .         .         .         .
Dsechellia     3850 AGATTACTGAGCCATAGCAGTCTTCTTGCAAGACGTCTCATCCCCGCTCGACCTCT
Dyakuba        1549 AGACTACAGAGTCATAGTAGCCTTCTTGCAAGACGTCTTATCCCCGCTCGACCTCT
Derecta             ........................................................
Dananassae     3752 AGACTTCTAAGACATCGCAGCCTACTGGCTAGAAATTTACTTCCAGCTAGACCATT
Dmojavensi     3910 AGACTTGTACGACATCAAAGCCTACTCGCAAGAGGTCTAACACCTGCCAGACCCCT
Dvirilis       3623 AAATTGCTGAGACACCGCAGCCTGCTCGCAAGAGGACTACTCCCTGCCCAACCTCT
Dsimulans      3876 AGATTACTGAGCCATAGCAGTCTTCTTGCAAGACGTCTCATCCCCGCTCGACCTCT
Dmelano        1144 AGATTACTGAGCCATAGCAGTCTTCTTGCAAGACGTCTTATCCCCGCTCGACCTCT
Dbuzzatii           ........................................................
Dkoep28             ........................................................
Dkoep351            ........................................................
Dkoep352            ........................................................
consensus>50        aga.t.ct.ag.cat.g.ag.ct.ct.gcaaga.gtct..t.cc.gc..gacc.ct
```
