# Supplementary material for: Expression of the Retrotransposon Helena Reveals a Complex Pattern of TE Deregulation in Drosophila Hybrids
Source: PLoS One. 2016 Jan 26;11(1):e0147903. doi: 10.1371/journal.pone.0147903 (PMC4728067; doi:10.1371/journal.pone.0147903)
Supplement: S3 Text — (PDF) [file pone.0147903.s010.pdf]

sample	generation	cross	Δct
males	buzzatii	A	14,07
males	buzzatii	B	11,95
males	buzzatii	C	14,05
males	buzzatii	E	12,95
males	buzzatii	F	14,29
males	buzzatii	G	12,47
males	buzzatii	H	12,54
males	buzzatii	I	11,97
males	buzzatii	J	13,43
males	buzzatii	L	8,44
males	buzzatii	M	10,08
males	F1	A	11,51
males	F1	B	14,49
males	F1	C	11,96
males	F1	D	4,49
males	F1	EFJMN	16,55
males	F1	GHI	13,45
males	BC1	A	11,68
males	BC1	B	11,56
males	BC1	C	13,24
males	BC1	D	12,82
males	BC1	E	9,19
males	BC1	H	15,39
males	BC1	I	11,87
males	BC1	J	12,16
males	BC1	K	12,62
males	BC1	M	14,62
males	BC1	N	13,06
males	BC2	A	11,76
males	BC2	B	10,3
males	BC2	C	10,36
males	BC2	D	10,41
males	BC2	E	13,07
males	BC2	H	13,56
males	BC2	I	12,03
males	BC2	J	13,52
males	BC2	K	7,85
males	BC2	M	12,25
males	BC3	A	11,74
males	BC3	B	11,88
males	BC3	C	11,07
males	BC3	D	13,86
males	BC3	E	12,06
males	BC3	F	14,3
males	BC3	G	12,74
males	BC3	H	17,32
males	BC3	M	10,25
males	koepferae		12,42
males	koepferae		12,46
males	koepferae		13,11

sample	generation	cross	Δct
females	koepferae	A	15,3
females	koepferae	B	15,5
females	koepferae	F	15,26
females	koepferae	H	12,89
females	koepferae	J	13,84
females	koepferae	K	13,98
females	koepferae	L	13,24
females	koepferae	M	12,25
females	koepferae	N	11,29
females	F1	A	13,47
females	F1	B	13,76
females	F1	C	13,29
females	F1	D	13,5
females	F1	E	12,34
females	F1	G	12,14
females	F1	K	10,7
females	F1	M	13,58
females	F1	N	11,07
females	BC1	B	12,94
females	BC1	C	13,38
females	BC1	D	13,07
females	BC1	E	12,89
females	BC1	F	14,44
females	BC1	G	15,02
females	BC1	H	13,26
females	BC1	I	12,9
females	BC1	J	11,55
females	BC1	K	12,03
females	BC1	L	13,22
females	BC1	M	12,64
females	BC1	N	15,73
females	BC2	A	13,08
females	BC2	B	12,73
females	BC2	C	12,69
females	BC2	D	11,79
females	BC2	E	13,64
females	BC2	FG	12,84
females	BC2	I	12,87
females	BC2	J	13,26
females	BC2	K	12,9
females	BC2	L	16,12
females	BC2	M	14,18
females	BC2	N	17,41
females	BC3	A	12,47
females	BC3	B	12,47
females	BC3	C	12,63
females	BC3	D	14,76
females	BC3	E	14,21
females	BC3	F	15,28
females	BC3	G	13,97
females	BC3	H	15,02
females	BC3	J	12,21
females	BC3	L	10,49
females	buzzatii		14,8
females	buzzatii		12,07
females	buzzatii		12,24

sample	generation	cross	Δct
testes	buzzatii	A	13,38
testes	buzzatii	B	11,9
testes	buzzatii	C	10,77
testes	buzzatii	D	11,08
testes	buzzatii	E	11,87
testes	buzzatii	G	11,64
testes	buzzatii	I	10,34
testes	buzzatii	J	11,26
testes	buzzatii	L	7,33
testes	buzzatii	M	8,12
testes	buzzatii	N	9,24
testes	F1	A	12,46
testes	F1	B	11,52
testes	F1	C	12,02
testes	F1	D	12,18
testes	F1	EFJMN	13,15
testes	BC1	A	10,36
testes	BC1	B	10,23
testes	BC1	C	10,05
testes	BC1	D	9,75
testes	BC1	E	11,12
testes	BC1	H	12,03
testes	BC1	I	9,82
testes	BC1	J	9,91
testes	BC1	K	9,99
testes	BC1	L	12,72
testes	BC1	N	10,87
testes	BC2	A	11,17
testes	BC2	B	10,16
testes	BC2	C	8,81
testes	BC2	D	10,2
testes	BC2	E	13,35
testes	BC2	F	13,38
testes	BC2	G	10,33
testes	BC2	H	11,45
testes	BC2	I	11,53
testes	BC2	J	11,37
testes	BC2	K	10,42
testes	BC2	L	16,32
testes	BC2	M	11,45
testes	BC2	N	9,46
testes	BC3	A	10,32
testes	BC3	B	10,54
testes	BC3	D	13,16
testes	BC3	E	11,53
testes	BC3	F	12,69
testes	BC3	G	14,56
testes	BC3	H	10,53
testes	BC3	I	11,63
testes	BC3	K	11,17
testes	BC3	N	11,06
testes	koepferae		10,12
testes	koepferae		10,65
testes	koepferae		9,74

sample	generation	cross	Δct
ovaries	koepferae	A	19,59
ovaries	koepferae	C	16,11
ovaries	koepferae	D	16,38
ovaries	koepferae	E	18,87
ovaries	koepferae	G	17,62
ovaries	koepferae	H	16,88
ovaries	koepferae	J	18,15
ovaries	koepferae	K	17,34
ovaries	koepferae	L	17,94
ovaries	koepferae	N	16,13
ovaries	F1	A	15,24
ovaries	F1	B	14,75
ovaries	F1	C	14,4
ovaries	F1	D	15,54
ovaries	F1	E	14,53
ovaries	F1	G	12,63
ovaries	F1	I	13,94
ovaries	F1	J	13,79
ovaries	F1	K	14,91
ovaries	F1	L	13,23
ovaries	F1	M	15,68
ovaries	F1	N	15,46
ovaries	BC1	A	14
ovaries	BC1	B	13,5
ovaries	BC1	C	13,2
ovaries	BC1	D	13,5
ovaries	BC1	E	15,13
ovaries	BC1	F	14,66
ovaries	BC1	G	14,43
ovaries	BC1	H	21,54
ovaries	BC1	I	14,45
ovaries	BC1	J	14,09
ovaries	BC1	K	13,44
ovaries	BC1	M	16,03
ovaries	BC1	N	13,51
ovaries	BC2	A	13,74
ovaries	BC2	B	13,88
ovaries	BC2	C	13,07
ovaries	BC2	D	13,57
ovaries	BC2	E	14,2
ovaries	BC2	F	13,68
ovaries	BC2	H	15,09
ovaries	BC2	I	14,75
ovaries	BC2	J	13,11
ovaries	BC2	K	13,49
ovaries	BC2	L	14,32
ovaries	BC2	M	13,63
ovaries	BC2	N	14,19
ovaries	BC3	A	15,83
ovaries	BC3	B	14,6
ovaries	BC3	C	15,06
ovaries	BC3	E	15,35
ovaries	BC3	F	15,33
ovaries	BC3	G	6,88
ovaries	BC3	H	14,85
ovaries	BC3	I	16,35
ovaries	BC3	J	15,5
ovaries	BC3	K	14,37
ovaries	BC3	L	15,78
ovaries	BC3	M	14,44
ovaries	buzzatii		13,5
ovaries	buzzatii		13,44
ovaries	buzzatii		12,06
ovaries	buzzatii		14,21
ovaries	buzzatii		11,53
